# The genome sequence of *Geobacter metallireducens*: features of metabolism, physiology and regulation common and dissimilar to *Geobacter sulfurreducens*

**DOI:** 10.1186/1471-2180-9-109

**Published:** 2009-05-27

**Authors:** Muktak Aklujkar, Julia Krushkal, Genevieve DiBartolo, Alla Lapidus, Miriam L Land, Derek R Lovley

**Affiliations:** 1 Department of Microbiology, University of Massachusetts Amherst, Amherst, MA, USA; 2 Department of Preventive Medicine and Center of Genomics and Bioinformatics, University of Tennessee Health Science Center, University of Tennessee, Memphis, TN, USA; 3 Department of Energy, Joint Genome Institute, Walnut Creek, CA, USA; 4 Oak Ridge National Laboratory, Oak Ridge, TN, USA

## Abstract

**Background:**

The genome sequence of *Geobacter metallireducens *is the second to be completed from the metal-respiring genus *Geobacter*, and is compared in this report to that of *Geobacter sulfurreducens *in order to understand their metabolic, physiological and regulatory similarities and differences.

**Results:**

The experimentally observed greater metabolic versatility of *G. metallireducens versus G. sulfurreducens *is borne out by the presence of more numerous genes for metabolism of organic acids including acetate, propionate, and pyruvate. Although *G. metallireducens *lacks a dicarboxylic acid transporter, it has acquired a second putative succinate dehydrogenase/fumarate reductase complex, suggesting that respiration of fumarate was important until recently in its evolutionary history. Vestiges of the molybdate (ModE) regulon of *G. sulfurreducens *can be detected in *G. metallireducens*, which has lost the global regulatory protein ModE but retained some putative ModE-binding sites and multiplied certain genes of molybdenum cofactor biosynthesis. Several enzymes of amino acid metabolism are of different origin in the two species, but significant patterns of gene organization are conserved. Whereas most *Geobacteraceae *are predicted to obtain biosynthetic reducing equivalents from electron transfer pathways via a ferredoxin oxidoreductase, *G. metallireducens *can derive them from the oxidative pentose phosphate pathway. In addition to the evidence of greater metabolic versatility, the *G. metallireducens *genome is also remarkable for the abundance of multicopy nucleotide sequences found in intergenic regions and even within genes.

**Conclusion:**

The genomic evidence suggests that metabolism, physiology and regulation of gene expression in *G. metallireducens *may be dramatically different from other *Geobacteraceae*.

## Background

*Geobacter metallireducens *is a member of the *Geobacteraceae*, a family of Fe(III)-respiring Delta-proteobacteria that are of interest for their role in cycling of carbon and metals in aquatic sediments and subsurface environments as well as the bioremediation of organic- and metal-contaminated groundwater and the harvesting of electricity from complex organic matter [1,2]. *G. metallireducens *is of particular interest because it was the first microorganism found to be capable of a number of novel anaerobic processes including: (1) conservation of energy to support growth from the oxidation of organic compounds coupled to the reduction of Fe(III) or Mn(IV) [3,4]; (2) conversion of Fe(III) oxide to ultrafine-grained magnetite [3]; (3) anaerobic oxidation of an aromatic hydrocarbon [5,6]; (4) reduction of U(VI) [7]; (5) use of humic substances as an electron acceptor [8]; (6) chemotaxis toward metals [9]; (7) complete oxidation of organic compounds to carbon dioxide with an electrode serving as the sole electron acceptor ([10]; and (8) use of a poised electrode as a direct electron donor [11]. Although the complete genome sequence of the closely related *Geobacter sulfurreducens *is available [12] and can provide insights into some of the common metabolic features of *Geobacter *species, *G. metallireducens *and *G. sulfurreducens *are significantly different in many aspects of their physiology. *G. sulfurreducens *is known to use only four carbon sources: acetate, formate, lactate (poorly) and pyruvate (only with hydrogen as electron donor), whereas *G. metallireducens *uses acetate, benzaldehyde, benzoate, benzylalcohol, butanol, butyrate, *p*-cresol, ethanol, *p*-hydroxybenzaldehyde, *p*-hydroxybenzoate, *p*-hydroxybenzylalcohol, isobutyrate, isovalerate, phenol, propionate, propanol, pyruvate, toluene and valerate [2].

Therefore, in order to gain broader insight into the physiological diversity of *Geobacter *species, the genome of *G. metallireducens *was sequenced and compared to that of *Geobacter sulfurreducens *[12]. Both genome annotations were manually curated with the addition, removal and adjustment of hundreds of protein-coding genes and other features. Phylogenetic analyses were conducted to validate the findings, including homologs from the finished and unfinished genome sequences of more distantly related *Geobacteraceae*. This paper presents insights into the conserved and unique features of two *Geobacter *species, particularly the metabolic versatility of *G. metallireducens *and the numerous families of multicopy nucleotide sequences in its genome, which suggest that regulation of gene expression is very different in these two species.

## Results and Discussion

### Contents of the two genomes

The automated annotation of the *G. metallireducens *genome identified 3518 protein-coding genes on the chromosome of 3997420 bp and 13 genes on the plasmid (designated pMET1) of 13762 bp. Manual curation added 59 protein-coding genes plus 56 pseudogenes to the chromosome and 4 genes to the plasmid. Ten of the chromosomal genes were reannotated as pseudogenes and another 22 were removed from the annotation. In addition to the 58 RNA-coding genes in the automated annotation, manual curation identified 479 conserved nucleotide sequence features. Likewise, to the 3446 protein-coding genes in the automated annotation of the *G. sulfurreducens *genome [12], manual curation added 142 protein-coding genes and 19 pseudogenes. Five genes were reannotated as pseudogenes and 103 genes were removed from the annotation. In addition to the 55 RNA-coding genes in the automated annotation, manual curation identified 462 conserved nucleotide sequence features. Of the 3629 protein-coding genes and pseudogenes in *G. metallireducens*, 2403 (66.2%) had one or more full-length homologs in *G. sulfurreducens*.

The nucleotide composition of the 3563 intact protein-coding genes of *G. metallireducens *was determined in order to identify some of those that were very recently acquired. The average G+C content of the protein-coding genes was 59.5%, with a standard deviation of 5.9%. Only three genes had a G+C content more than two standard deviations above the mean (> 71.2%), but 146 genes had a G+C content more than two standard deviations below the mean (< 47.7%), most of which lack homologs in *G. sulfurreducens *and may be recent acquisitions (Additional file 1: Table S1). Clusters of such genes (shaded in Additional file 1: Table S1) were often interrupted or flanked by transposons with higher G+C content. The functions of most of these genes cannot be assigned at present, but 23 of them are predicted to act in cell wall biogenesis.

Plasmid pMET1 of *G. metallireducens *consists of a series of six predicted transcriptional units on one strand, tentatively attributed to the mobilization (Gmet_A3575-Gmet_A3574-Gmet_A3573-Gmet_A3572-Gmet_A3643), entry exclusion (Gmet_A3571), addiction (Gmet_A3570-Gmet_A3579-Gmet_A3642), partition (Gmet_A3568-Gmet_A3641), transposition (Gmet_A3567), and replication (Gmet_A3566-Gmet_A3565) functions of the plasmid, and one operon on the opposite strand, comprised of three genes of unknown function (Gmet_A3576-Gmet_A3577-Gmet_A3644). The predicted origin of replication, located 3' of the *repA *gene (Gmet_A3565), includes four pairs of iterons and a set of six hairpins, suggesting that pMET1 replicates by a rolling-circle mechanism, although it is significantly larger than most such plasmids [13]. Among the fifteen other nucleotide sequence features identified on the plasmid during manual curation was a palindromic putative autoregulatory site (TTTGTTATACACGTATAACAAA) located 5' of the addiction module. Other than the potential toxicity of the addiction module, the impact of pMET1 on the physiology of *G. metallireducens *is unknown.

### Metabolism of acetate and other carbon sources

**Figure 1:**
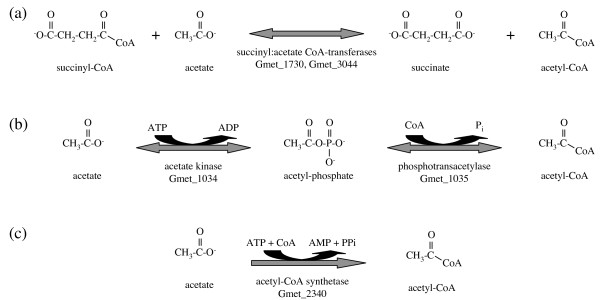
**Pathways of acetate activation in *G. metallireducens***. (a) The succinyl:acetate CoA-transferase reaction. (b) The acetate kinase and phosphotransacetylase reactions. (c) The acetyl-CoA synthetase reaction.

Acetate is expected to be the key electron donor supporting Fe(III) reduction in aquatic sediments and subsurface environments [14], and *Geobacter *species quickly become the predominant bacterial species when acetate is injected into subsurface environments to promote *in situ *bioremedation of uranium-contaminated groundwater [15,16]. Surprisingly, the initial activation of acetate by ligation with coenzyme A (CoA) in *G. sulfurreducens *occurs by two reversible pathways [17] (Figure 1), indicating that acetate may be inefficiently utilized at low concentrations. These two pathways are also present in *G. metallireducens*, along with a third, irreversible reaction that may permit efficient activation of acetate at low concentrations. The first pathway of acetate activation (Figure 1a) occurs through either of two succinyl:acetate CoA-transferases that can convert succinyl-CoA to succinate during oxidation of acetate by the tricarboxylic acid (TCA) cycle pathway, in the same capacity as succinyl-CoA synthetase but conserving energy in the form of acetyl-CoA rather than GTP or ATP [17]. Microarray data from both species suggest that expression of one succinyl:acetate CoA-transferase isoenzyme (Gmet_1730 = GSU0174) is constant and expression of the other (Gmet_3044 = GSU0490) is induced during acetate-fueled growth with electron acceptors other than soluble Fe(III), such as Fe(III) oxides, nitrate, or fumarate (D. Holmes, B. Postier, and R. Glaven, personal communications). The second pathway (Figure 1b) consists of two steps: acetate kinase (Gmet_1034 = GSU2707) converts acetate to acetyl-phosphate, which may be a global intracellular signal affecting various phosphorylation-dependent signalling systems, as in *Escherichia coli *[18]; and phosphotransacetylase (Gmet_1035 = GSU2706) converts acetyl-phosphate to acetyl-CoA [17]. *G. metallireducens *possesses orthologs of the enzymes of both pathways characterized in *G. sulfurreducens *[17], and also has an acetyl-CoA synthetase (Gmet_2340, 42% identical to the *Bacillus subtilis *enzyme [19]) for irreversible activation of acetate to acetyl-CoA at the expense of two ATP (Figure 1c). Thus, *Geobacteraceae *such as *G. metallireducens *may be better suited to metabolize acetate at the low concentrations naturally found in most soils and sediments.

Three enzymes distantly related to the succinyl:acetate CoA-transferases are encoded by Gmet_2054, Gmet_3294, and Gmet_3304, for which there are no counterparts in *G. sulfurreducens*. All three of these proteins closely match the characterized butyryl:4-hydroxybutyrate/vinylacetate CoA-transferases of *Clostridium *species [20]. However, their substrate specificities may be different because the *G. metallireducens *proteins and the *Clostridium *proteins cluster phylogenetically with different CoA-transferases of *Geobacter *strain FRC-32 and *Geobacter bemidjiensis *(data not shown). The presence of these CoA-transferases indicates that *G. metallireducens *has evolved energy-efficient activation steps for some unidentified organic acid substrates that *G. sulfurreducens *cannot utilize.

Numerous other enzymes of acyl-CoA metabolism are predicted from the genome of *G. metalllireducens *but not that of *G. sulfurreducens *(Additional file 2: Table S2), including six gene clusters, three of which have been linked to degradation of aromatic compounds that *G. metallireducens *can utilize [6,21-23] but *G. sulfurreducens *cannot [24]. All seven acyl-CoA synthetases of *G. sulfurreducens *have orthologs in *G. metallireducens*, but the latter also possesses acetyl-CoA synthetase, benzoate CoA-ligase (experimentally validated [23]), and seven other acyl-CoA synthetases of unknown substrate specificity. The *G. metallireducens *genome also includes eleven acyl-CoA dehydrogenases, three of which are specific for benzylsuccinyl-CoA (69% identical to the *Thauera aromatica *enzyme [25]), glutaryl-CoA (experimentally validated [26]) and isovaleryl-CoA (69% identical to the *Solanum tuberosum *mitochondrial enzyme [27]), whereas none can be identified in *G. sulfurreducens*. *G. metallireducens *also has nine pairs of electron transfer flavoprotein genes (seven of which are adjacent to genes encoding iron-sulfur cluster-binding proteins) that are hypothesized to connect acyl-CoA dehydrogenases to the respiratory chain, whereas *G. sulfurreducens *has only one. None of the seventeen enoyl-CoA hydratases of *G. metallireducens *is an ortholog of GSU1377, the sole enoyl-CoA hydratase of *G. sulfurreducens*. *G. metallireducens *also possesses eleven acyl-CoA thioesterases, of which *G. sulfurreducens *has orthologs of five plus the unique thioesterase GSU0196. Of the ten acyl-CoA thiolases of *G. metallireducens*, only Gmet_0144 has an ortholog (GSU3313) in *G. sulfurreducens*. BLAST searches and phylogenetic analyses demonstrated that several of these enzymes of acyl-CoA metabolism have close relatives in *G. bemidjiensis*, *Geobacter *FRC-32, *Geobacter lovleyi *and *Geobacter uraniireducens*, indicating that their absence from *G. sulfurreducens *is due to gene loss, and that this apparent metabolic versatility is largely the result of expansion of enzyme families within the genus *Geobacter *(data not shown). The ability of *G. metallireducens *and other *Geobacteraceae *to utilize carbon sources that *G. sulfurreducens *cannot utilize may be due to stepwise breakdown of multicarbon organic acids to simpler compounds by these enzymes.

Growth of *G. metallireducens *on butyrate may be attributed to reversible phosphorylation by either of two butyrate kinases (Gmet_2106 and Gmet_2128), followed by reversible CoA-ligation by phosphotransbutyrylase (Gmet_2098), a pathway not present in *G. sulfurreducens*, which cannot grow on butyrate [24]. These gene products are 42–50% identical to the enzymes characterized in *Clostridium beijerinckii *and *Clostridium acetobutylicum *[28,29].

**Figure 2:**
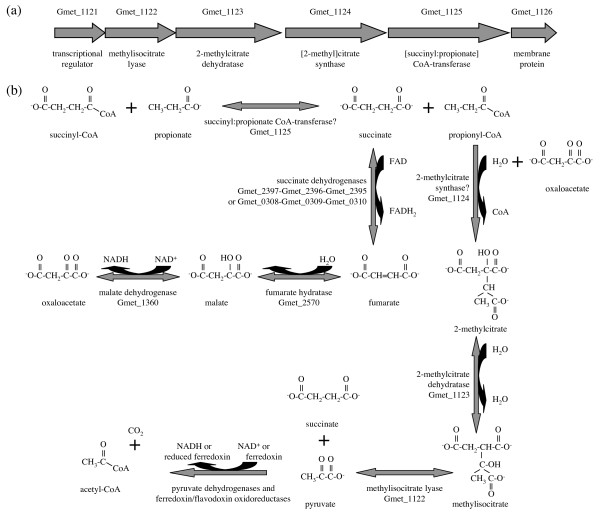
**Growth of *G. metallireducens *on propionate**. (a) The gene cluster predicted to encode enzymes of propionate metabolism. (b) The proposed pathway of propionate metabolism.

An enzyme very similar to succinyl:acetate CoA-transferase is encoded by Gmet_1125 within the same operon as methylisocitrate lyase (Gmet_1122), 2-methylcitrate dehydratase (Gmet_1123), and a citrate synthase-related protein hypothesized to be 2-methylcitrate synthase (Gmet_1124) [30] (Figure 2a), all of which are absent in *G. sulfurreducens*. This arrangement of genes, along with the ability of *G. metallireducens *to utilize propionate as an electron donor [31] whereas *G. sulfurreducens *cannot [24], suggests that the Gmet_1125 protein could be a succinyl:propionate CoA-transferase that, together with the other three products of the operon, would convert propionate (via propionyl-CoA) and oxaloacetate to pyruvate and succinate (Figure 2b). Upon oxidation of succinate to oxaloacetate through the TCA cycle and oxidative decarboxylation of pyruvate to acetyl-CoA, the pathway would be equivalent to the breakdown of propionate into six electrons, one molecule of carbon dioxide, and acetate, followed by the succinyl:acetate CoA-transferase reaction (Figure 2b). In a phylogenetic tree, the hypothetical succinyl:propionate CoA-transferase Gmet_1125 and gene Geob_0513 of *Geobacter *FRC-32, which is also capable of growth with propionate as the sole electron donor and carbon source (M. Aklujkar, unpublished), form a branch adjacent to succinyl:acetate CoA-transferases of the genus *Geobacter *(data not shown). In a similar manner, the hypothetical 2-methylcitrate synthase Gmet_1124 and gene Geob_0514 of *Geobacter *FRC-32 form a branch adjacent to citrate synthases of *Geobacter *species (data not shown), consistent with the notion that these two enzyme families could have recently evolved new members capable of converting propionate via propionyl-CoA to 2-methylcitrate.

Gmet_0149 (GSU3448) is a homolog of acetate kinase that does not contribute sufficient acetate kinase activity to sustain growth of *G. sulfurreducens *[17] and has a closer BLAST hit to propionate kinase of *E. coli *(40% identical sequence) than to acetate kinase of *E. coli*. Although it does not cluster phylogenetically with either of the *E. coli *enzymes, its divergence from acetate kinase (Gmet_1034 = GSU2707) is older than the last common ancestor of the *Geobacteraceae *(data not shown). This conserved gene product remains to be characterized as a propionate kinase or something else.

The proposed pathway for growth of *G. metallireducens *on propionate (Figure 2) is contingent upon its experimentally established ability to grow on pyruvate [31]. *G. sulfurreducens *cannot utilize pyruvate as the carbon source unless hydrogen is provided as an electron donor [17]. Oxidation of acetyl-CoA derived from pyruvate in *G. sulfurreducens *may be prevented by a strict requirement for the succinyl:acetate CoA-transferase reaction (thermodynamically inhibited when acetyl-CoA exceeds acetate) to complete the TCA cycle in the absence of detectable activity of succinyl-CoA synthetase (GSU1058-GSU1059) [17]. With three sets of succinyl-CoA synthetase genes (Gmet_0729-Gmet_0730, Gmet_2068-Gmet_2069, and Gmet_2260-Gmet_2261),* G. metallireducens *may produce enough activity to complete the TCA cycle.

*G. sulfurreducens *and *G. metallireducens *may interconvert malate and pyruvate through a malate oxidoreductase fused to a phosphotransacetylase-like putative regulatory domain (*maeB*; Gmet_1637 = GSU1700), which is 51% identical to the NADP^+^-dependent malic enzyme of *E. coli *[32]. *G. sulfurreducens *has an additional malate oxidoreductase without this fusion (*mleA*; GSU2308) that is 53% identical to an NAD^+^-dependent malic enzyme of *B. subtilis *[33], but *G. metallireducens *does not.

**Figure 3:**
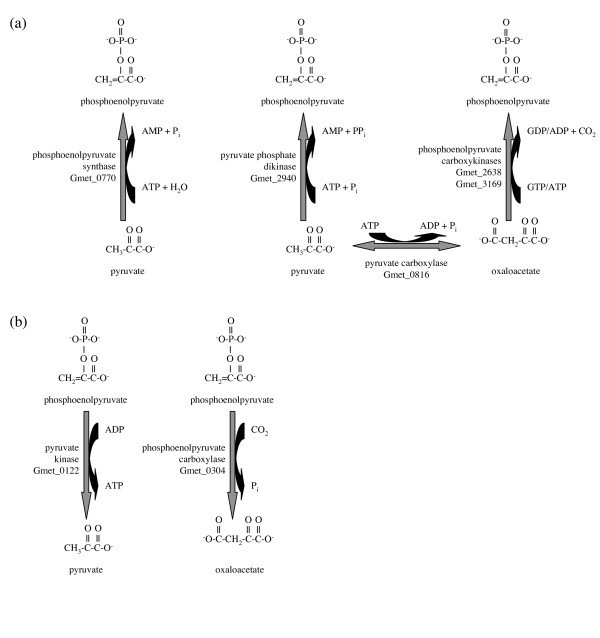
**Potential futile cycling of pyruvate/oxaloacetate and phosphoenolpyruvate in *G. metallireducens***. (a) Conversion of pyruvate to phosphoenolpyruvate. (b) Conversion of phosphoenolpyruvate to pyruvate or oxaloacetate.

*G. metallireducens *possesses orthologous genes for all three pathways that activate pyruvate or oxaloacetate to phosphoenolpyruvate in *G. sulfurreducens *(Figure 3a): phosphoenolpyruvate synthase (Gmet_0770 = GSU0803), pyruvate phosphate dikinase (Gmet_2940 = GSU0580) and GTP-dependent phosphoenolpyruvate carboxykinase Gmet_2638 = GSU3385) [17]. It also encodes a homolog of the ATP-dependent phosphoenolpyruvate carboxykinase of *E. coli *(Gmet_3169, 48% identical) that has no homolog in *G. sulfurreducens*. In the catabolic direction, in addition to pyruvate kinase (Gmet_0122 = GSU3331) that converts phosphoenolpyruvate to pyruvate plus ATP, *G. metallireducens *has a homolog of *E. coli *phosphoenolpyruvate carboxylase (Gmet_0304, 30% identical, also found in *Geobacter *FRC-32) that may convert phosphoenolpyruvate to oxaloacetate irreversibly (Figure 3b) and contribute to the observed futile cycling of pyruvate/oxaloacetate/phosphoenolpyruvate [34] if not tightly regulated. Thus, control of the fate of pyruvate appears to be more complex in *G. metallireducens *than in *G. sulfurreducens*.

### Evidence of recent fumarate respiration in *G. metallireducens*

**Figure 4:**
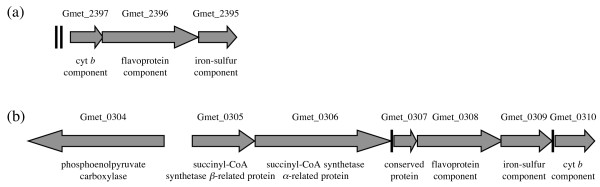
**Acquisition of a second fumarate reductase/succinate dehydrogenase by *G. metallireducens***. (a) The ancestral gene cluster. (b) The gene cluster acquired from a relative of the *Chlorobiaceae*, located near other acquired genes relevant to central metabolism: an uncharacterized enzyme related to succinyl-CoA synthetase and citrate synthase (Gmet_0305-Gmet_0306) and phosphoenolpyruvate carboxylase (Gmet_0304). Conserved nucleotide sequences (black stripes) were also identified in the two regions.

The succinate dehydrogenase complex of *G. sulfurreducens *also functions as a respiratory fumarate reductase, possibly in association with a co-transcribed *b*-type cytochrome [35]. *G. metallireducens *has homologous genes (Gmet_2397-Gmet_2395 = GSU1176-GSU1178), but is unable to grow with fumarate as the terminal electron acceptor unless transformed with a plasmid that expresses the dicarboxylic acid exchange transporter gene *dcuB *of *G. sulfurreducens *[35], which has homologues in *Geobacter *FRC-32, *G. bemidjiensis*, *G. lovleyi*, and *G. uraniireducens*. Surprisingly, *G. metallireducens *has acquired another putative succinate dehydrogenase or fumarate reductase complex (Gmet_0308-Gmet_0310), not found in other *Geobacteraceae*, by lateral gene transfer from a relative of the *Chlorobiaceae *(phylogenetic trees not shown), and evolved it into a gene cluster that includes enzymes of central metabolism acquired from other sources (Figure 4). Thus, *G. metallireducens *may have actually enhanced its ability to respire fumarate before recently losing the requisite transporter.

### Nitrate respiration and loss of the *modE *regulon from *G. metallireducens*

**Figure 5:**
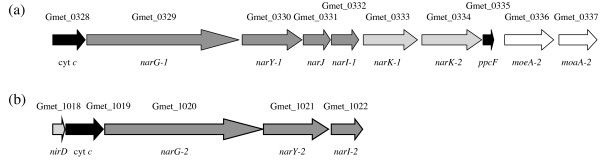
**The respiratory nitrate reductase operons**. (a) The major (expressed) operon also encodes the nitrate and nitrite transporters (*narK-1*, *narK-2*), two *c*-type cytochromes including *ppcF*, and two genes of molybdenum cofactor biosynthesis (*moeA-2*, *moaA-2*). (b) The minor operon (expression not detected) also encodes the Rieske iron-sulfur component of nitrite reductase (*nirD*) and a *c*-type cytochrome, but lacks a *narJ *gene.

*G. metallireducens *is able to respire nitrate [4], whereas *G. sulfurreducens *cannot [24]. The nitrate reductase activity of *G. metallireducens *is attributed to the *narGYJI *genes (Figure 5a; Gmet_0329-Gmet_0332), which are adjacent to the *narK-1 *and *narK-2 *genes encoding a proton/nitrate symporter and a nitrate/nitrite antiporter (Gmet_0333 and Gmet_0334, respectively) predicted according to homology with the two halves of *narK *in *Paracoccus pantotrophus *[36]. A second *narGYI *cluster (Figure 5b; Gmet_1020 to Gmet_1022) is missing a noncatalytic subunit (*narJ*), and its expression has not been detected (B. Postier, personal communication). The first gene of both operons encodes a unique diheme *c*-type cytochrome (Gmet_0328 and Gmet_1019), suggesting that the nitrate reductase may be connected to other electron transfer components besides the menaquinol pool, perhaps operating in reverse as a nitrite oxidase. The product of the *ppcF *gene (Gmet_0335) in the intact *nar *operon, which is related to a periplasmic triheme *c*-type cytochrome involved in Fe(III) reduction in *G. sulfurreducens *[37], may permit electron transfer to the nitrate reductase from extracellular electron donors such as humic substances [38] or graphite electrodes [11]. The final two genes of the intact *nar *operon (Gmet_0336-Gmet_0337), encode the MoeA and MoaA enzymes implicated in biosynthesis of *bis*-(molybdopterin guanine dinucleotide)-molybdenum, an essential cofactor of the nitrate reductase.

**Figure 6:**
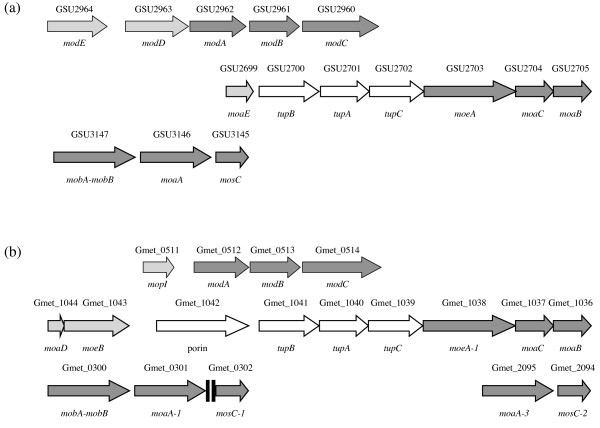
***G. sulfurreducens *and *G. metallireducens *possess different genes for molybdenum cofactor biosynthesis**. (a) *G. sulfurreducens *has the global regulator *modE*. (b) *G. metallireducens *has multiple copies of *moeA*, *moaA*, and *mosC*, and putative integration host factor binding sites (black stripes). Both genomes have conserved genes (dark grey) for molybdate transport (*modABC*) and molybdopterin biosynthesis (*moeA*, *moaCB*, *mobA-mobB*, *mosC*) alongside *tup *genes for tungstate transport (white), but neither genome has all the genes thought to be essential for *bis*-(molybdopterin guanine dinucleotide)-molybdenum biosynthesis (light grey). See also Table 1.

Phylogenetic analysis indicates that the *moeA *and *moaA *gene families have repeatedly expanded in various *Geobacteraceae *(data not shown). *G. sulfurreducens *has a single copy of each, but *G. metallireducens *has three closely related isoenzymes, of which *moeA-1 *(Gmet_1038 = GSU2703, 40% identical to the *E. coli *protein [39]) and *moaA-1 *(Gmet_0301 = GSU3146, 36% identical to the *E. coli *protein [40]) occupy a conserved location among other genes of molybdopterin biosynthesis (Table 1, Figure 6). A possible reason for the expansion in *G. metallireducens *and other *Geobacteraceae *is a need to upregulate molybdopterin biosynthesis for specific processes: *moeA-2 *and *moaA-2 *(Gmet_0336-Gmet_0337, 38% and 33% identity to the *E. coli *proteins) may support nitrate reduction; *moaA-3 *(Gmet_2095, 35% identity to *E. coli*) may function with nearby gene clusters for catabolism of benzoate [23] and *p*-cresol [22]; and *moeA-3 *(Gmet_1804, 37% identity to *E. coli*) may aid growth on benzoate, during which it is upregulated [21]. *G. metallireducens *differs from *G. sulfurreducens *in other aspects of molybdenum assimilation as well (Table 1): notably, *G. sulfurreducens *possesses a homolog of the *moaE *gene (GSU2699) encoding the large subunit of molybdopterin synthase, but lacks homologs of the small subunit gene *moaD *and the molybdopterin synthase sulfurylase gene *moeB*, whereas *G. metallireducens *lacks a *moaE *homolog but possesses homologs of *moaD *(Gmet_1043) and *moeB *(Gmet_1042). Comparison with the genomes of other *Geobacteraceae *suggests that these differences are due to loss of ancestral genes. How the nitrate reductase of *G. metallireducens *can function with the molybdopterin synthase complex being apparently incomplete is unknown.

**Table 1:** Genes of molybdenum cofactor biosynthesis in *G. sulfurreducens *and *G. metallireducens*.

Locus	Gene in *G. sulfurreducens*	Gene in *G. metallireducens*	Function
*modE*	GSU2964	Gmet_0511^1^	regulation of molybdate-responsive genes
*modD*	GSU2963	none	inner membrane protein, possible quinolinate phosphoribosyltransferase
*modA*	GSU2962	Gmet_0512	molybdate transport (periplasmic component)
*modB*	GSU2961	Gmet_0513	molybdate transport (membrane component)
*modC*	GSU2960	Gmet_0514	molybdate transport (ATP-binding component)
*moaD*	none	Gmet_1044	dithiolene addition to molybdopterin (molybdopterin synthase small subunit)
*moeB*	none	Gmet_1043	molybdopterin synthase sulfurylase
*moaE*	GSU2699	none	dithiolene addition to molybdopterin (molybdopterin synthase large subunit)
*moeA*	GSU2703	Gmet_1038; Gmet_0336; Gmet_1804	molybdenum-sulfur ligation?
*moaC*	GSU2704	Gmet_1037	molybdopterin precursor Z synthesis
*moaB*	GSU2705	Gmet_1036	molybdopterin precursor Z synthesis
*mobA*	GSU3147 N-terminal domain	Gmet_0300 N-terminal domain	attachment of molybdopterin to guanosine
*mobB*	GSU3147 C-terminal domain	Gmet_0300 C-terminal domain	attachment of molybdopterin to guanosine
*moaA*	GSU3146	Gmet_0301; Gmet_0337; Gmet_2095	molybdopterin precursor Z synthesis
*mosC*	GSU3145	Gmet_0302; Gmet_2094	molybdenum sulfurase
*pcmV*	none	Gmet_2138	possible 4-hydroxybenzoyl-CoA reductase molybdenum cofactor biosynthesis protein
*pcmW*	none	Gmet_2139	possible 4-hydroxybenzoyl-CoA reductase molybdenum cofactor biosynthesis protein
*pcmX*	none	Gmet_2140	uncharacterized protein related to MobA

^1^Gmet_0511 is missing the N-terminal ModE domain but retains the C-terminal molybdopterin-binding MopI domains.

In *G. sulfurreducens*, putative binding sites for the molybdate-sensing ModE protein (GSU2964) have been identified by the ScanACE software [41,42] in several locations, and the existence of a ModE regulon has been predicted [43]. The genes in the predicted ModE regulon (Additional file 3: Table S3) include one of the two succinyl:acetate CoA-transferases, a glycine-specific tRNA (anticodon CCC, corresponding to 26% of glycine codons), several transport systems, and some nucleases. In *G. metallireducens*, there is no full-length *modE *gene, but a gene encoding the C-terminal molybdopterin-binding (MopI) domain of ModE (Gmet_0511) is present in the same location (Figure 6). Phylogenetic analysis shows that the Gmet_0511 gene product is the closest known relative of *G. sulfurreducens *ModE, and that it has evolved out of the *Geobacteraceae/Chlorobiaceae *cluster of full-length ModE proteins by loss of the N-terminal ModE-specific domain (data not shown). The ScanACE software detected only one of the ModE-binding sites of *G. sulfurreducens *at the corresponding location in the *G. metallireducens *genome, but some vestigial sites were apparent when other syntenous locations were visually inspected (Additional file 3: Table S3), indicating that the ModE regulon once existed in *G. metallireducens*, but recent loss of the ModE N-terminal domain is allowing the regulatory sites to disappear gradually over the course of genome sequence evolution due to the absence of selective pressure for these sites to remain conserved. Thus, genes that may be controlled globally by ModE in *G. sulfurreducens *and other *Geobacteraceae *to optimize molybdenum cofactor-dependent processes have recently acquired independence in *G. metallireducens*.

### Amino acid biosynthesis and its regulation

The two genomes differ in several aspects of amino acid biosynthesis and its regulation. To make aspartate from oxaloacetate, a homolog of *Bacillus circulans *aspartate aminotransferase [44] is present in *G. metallireducens *(Gmet_2078; 65% identical), whereas a homolog of the *Sinorhizobium meliloti *enzyme [45] is found in *G. sulfurreducens *(GSU1242; 52% identical). Both species possess asparagine synthetase (Gmet_2172 = GSU1953 and Gmet_2024, 30% and 24% identical to *asnB *of *B. subtilis *[46]) and glutamine synthetase (Gmet_1352 = GSU1835, 61% identical to *glnA *of *Fremyella diplosiphon *[47]), as well as an aspartyl/glutamyl-tRNA(Asn/Gln) amidotransferase operon (Gmet_0076, Gmet_0075, Gmet_0073 = GSU3383, GSU3381, GSU3380, 36–53% identical to the homologous subunits in *B. subtilis *[48]) that includes glutamine synthetase adenylyltransferase (*glnE*; Gmet_0071 = GSU3378). The *G. sulfurreducens glnE *gene may be inactive due to a deletion of ~ 45 codons in the C-terminal domain.

For biosynthesis of lysine, threonine and methionine, *G. metallireducens *and other *Geobacteraceae *possess a linked pair of aspartate-4-semialdehyde dehydrogenase genes: *Pseudomonas aeruginosa*-type Gmet_0603 (69% identity) [49] and *Mycobacterium bovis*-type Gmet_0604 (47% identity) [50], but *G. sulfurreducens *has only the former (GSU2878). A haloacid dehalogenase family protein (Gmet_1630 = GSU1694) encoded between two genes of the threonine biosynthesis pathway could be the enzyme required to complete the pathway, a phosphoserine:homoserine phosphotransferase analogous to that of *P. aeruginosa *[51], and may overlap functionally with the unidentified phosphoserine phosphatase required to complete the biosynthetic pathway of serine.

Conserved nucleotide sequences (possible promoters and riboswitches) were identified on the 5' sides of several biosynthetic operons (Table 2). The lysine biosynthesis operon in *G. sulfurreducens *and other *Geobacteraceae *begins with a *P. aeruginosa*-type *meso*-diaminopimelate decarboxylase (GSU0158; 51% identity) [52], whereas *G. metallireducens *has two isoenzymes in other locations (Gmet_0219, 30% identical to the *E. coli *enzyme [53], with homologs in a few *Geobacteraceae*; Gmet_2019, 31% identical to the *P. aeruginosa *enzyme [52], unique to *G. metallireducens*). The recently identified L,L-diaminopimelate aminotransferase (*dapL*; Gmet_0213 = GSU0162) [54] is co-transcribed with the *dapAB *genes encoding the two preceding enzymes of lysine biosynthesis, but separated from them by a predicted short RNA element (Gmet_R1005 = GSU0160.1), also found in 23 other locations on the *G. metallireducens *chromosome (Additional file 4: Figure S1, Additional file 5: Table S4).

**Table 2:** Conserved nucleotide sequences 5' of biosynthetic operons.

Operon	Locus tag and sequence coordinates	
	
	*G. metallireducens*	*G. sulfurreducens*
aspartyl/glutamyl-tRNA(Asn/Gln) amidotransferase (*gatCAB-mtnA-glnE-nth*)	Gmet_P0076 93465..93502	GSU3383.1 3719308..3719345
lysine (*dapA*)	Gmet_P0211 244588..244640	GSU0157.1 176066..176117
aromatic amino acids (*aroG-2*)	Gmet_R0069 384337..384528	GSUR082 3450692..3450963
cobalamin (*cobUTSCB*-*thiC-2*; *cbiM-1*-*cbiQ-1*-*cbiO-1*-*cbiX*-*cobH*-*cbiD*-*cobLIM*-*cbiG*-*cobQ*-*cbiB*-*cobD*)	Gmet_R0070 513498..513761 no match	GSU3011.1 3302884..3303201 GSU3004.1 3296929..3297108
methionine (*metC-1-metC-2*; *metX*)^1^	Gmet_R0073 765279..765444 Gmet_R0129 3145553..3145656	GSUR063 1014004..1014271 GSU2461.2 2700118..2700220
leucine (*leuA*)^2^	Gmet_P1265 1425160..1425452	GSU1906.1 2085440..2085740
leucine/isoleucine (*leuCD*)	Gmet_P1268 1428650..1428793	GSU1903.1 2082057..2082203
coenzyme A (*panBC*)	Gmet_P1642 1843163..1843275	GSU1704.1 1868745..1868863
pyrimidines (*pyrRBC-carAB*)	Gmet_P1768 1983157..1983191	GSU1269.1 1384886..1384920
tryptophan (*iorAB-paaK*)	Gmet_P1827 2042198..2042288	GSU1739.1 1905464..1905561
purines, pyrimidines (*purMN*, *rimI-pyrKD*)	Gmet_P1844 2056600..2056732	GSU1757.1 1920275..1920400
guanine (*guaBA*)	Gmet_P2293 2600787..2600857	GSU2195.1 2408782..2408854
serine (*serA*)	Gmet_P2378 2689446..2689518	GSU1197.1 1301091..1301163
thiamin (*thiE/D*-*thiC-1*; *thiS-1-thiG*-*tenI*)^3^	Gmet_R0131 3292750...3292897 Gmet_R0134 3319520..3319741	GSUR060 640780..640988 GSU0589.1 622533..622801
arginine (*argBDFG*)	Gmet_P0203 3719308..3719345	GSU0149.1 167623..167663

^1^The sequence 5' of *metC-1*, *metC-2*, and *metX *is a SAM-responsive riboswitch.

^2^The sequence 5' of *leuA *is a T-box, an RNA structure that recognizes the aminoacylation state of tRNA.

^3^The sequence 5' of thiamin biosynthesis operons is a thiamin diphosphate-responsive riboswitch.

*S*-adenosylmethionine (SAM)-responsive riboswitches (Table 2) may regulate homoserine *O*-acetyltransferase (Gmet_2783 = GSU2462, 45% identical to the *Leptospira meyeri *enzyme [55]), the first dedicated enzyme of methionine biosynthesis, and also two linked cystathionine-*γ*-synthase/cystathionine-*β*-lyase genes (Gmet_0698 = GSU0944; Gmet_0699 = GSU0945, 49% and 51% identical to the *Lactococcus lactis *lyase [56,57]). Phylogenetic analysis could not distinguish the synthase from the lyase (data not shown), but their presence suggests that homocysteine can be made by transsulfuration of homoserine with cysteine, and not only by the putative *O*-acetylhomoserine sulfhydrylases (Gmet_0819 = GSU2425, Gmet_2390 = GSU1183 and Gmet_1566, 47%, 56% and 38% identical to the *Emericella nidulans *enzyme [58], respectively). In *G. metallireducens*, transsulfuration may also be controlled by a GC-rich element between Gmet_0698 and Gmet_0699, which contains four tandem repeats of the heptanucleotide GGGACCG and is found in 49 intergenic and intragenic locations in the genome (Additional file 6: Figure S2, Additional file 5: Table S4).

The leucine pathway-specific *leuA *gene (2-isopropylmalate synthase; Gmet_1265 = GSU1906, 49% identical to the *E. coli *enzyme [59]) may be controlled by feedback inhibition through a T-box [60] predicted to form an antiterminator structure in response to uncharged leucine-specific tRNA having the GAG anticodon (Gmet_R0037 = GSUR030) (Table 2), putatively the only tRNA capable of recognizing 55% of leucine codons in *G. metallireducens *and 48% in *G. sulfurreducens *(CTC and CTT).

There are three 3-deoxy-D-arabino-heptulosonate-7-phosphate (DAHP) synthase isoenzymes to catalyze the first step of aromatic amino acid biosynthesis: one similar to *aroF *of *E. coli *(Gmet_2375 = GSU2291, 55% identity [61], but with a P148T substitution incompatible with feedback inhibition by tyrosine [62]) and two *Thermotoga maritima*-type enzymes (Gmet_0024 = GSU3333; Gmet_0346 = GSU3142, 51% and 46% identity [63], respectively). As one chorismate mutase is fused to prephenate dehydratase (*pheA*; Gmet_0862 = GSU2608, 41% identical to the *Pseudomonas stutzeri *fusion protein [64]), the other (Gmet_1955 = GSU1828, 30% identical to the chorismate mutase domain of the *P. stutzeri *fusion protein) may function predominantly in tyrosine biosynthesis, possibly regulated by the adjacent gene product (Gmet_1956 = GSU1829) that resembles the phenylalanine/tyrosine-responsive domain of *T. maritima *DAHP synthase [65]. Gmet_1956 orthologs phylogenetically cluster with the regulatory domains of Gmet_0024 orthologs (data not shown), suggesting that Gmet_0024 may be a tyrosine-inhibited DAHP synthase and Gmet_0346 may be inhibited by another end product such as phenylalanine. A predicted short RNA element (Gmet_R0069 = GSUR082, Table 2), found 5' of Gmet_0346 and its orthologs in several *Geobacteraceae*, may participate in regulation of this isoenzyme's expression.

In all non-*Geobacteraceae *that possess an indole-scavenging tryptophan synthase *β*2 protein, it is encoded apart from the *trp *operon containing the *trpAB1 *genes for the *α *(indole-producing) and *β*1 (indole-consuming) subunits of tryptophan synthase [66]. In *G. metallireducens *and *G. sulfurreducens*, however, the *β*2 gene *trpB2 *(Gmet_2493 = GSU2379, 60% identical to the *T. maritima *protein [67]) is the penultimate gene of the predicted *trp *operon and the *trpB1 *(Gmet_2482 = GSU2375, 66% identical to the *Acinetobacter calcoaceticus *protein [68]) and *trpA *(Gmet_2477 = GSU2371, 47% identical to the *Azospirillum brasilense *protein [69]) genes are separated from the 3' end of the operon and from each other by three or more intervening genes, most of which are not conserved between the two genomes (not shown). Next to the *trpB2 *gene of *G. metallireducens *is one of 24 pairs of a conserved nucleotide motif (Additional file 7: Figure S3, Additional file 5: Table S4) hypothesized to bind an unidentified global regulator protein. Other, evolutionarily related paired sites where another unidentified global regulator may bind (Additional file 8: Figure S4, Additional file 5: Table S4) are found in 21 locations. Between the *proBA *genes of *G. metallireducens*, encoding the first two enzymes of proline biosynthesis (Gmet_3198-Gmet_3199 = GSU3212-GSU3211, 41% and 45% identical to the *E. coli *enzymes [70]), is one of eight pairs of predicted binding sites for yet another unidentified global regulator (Additional file 9: Figure S5, Additional file 5: Table S4). In *G. sulfurreducens*, the space between *proBA *is occupied by a different conserved nucleotide sequence (not shown), found only in four other places in the same genome. Overall, a comparison of the two genomes offers insight into unique features of amino acid biosynthesis and its regulation that deserve further study.

### Nucleotide metabolism

Differences in nucleotide metabolism were identified in the two genomes. *G. metallireducens *has acquired a possibly redundant large subunit of carbamoyl-phosphate synthetase (Gmet_0661, 50% identical to the *P. aeruginosa *protein [71]) in addition to the ancestral gene (Gmet_1774 = GSU1276, 65% identity to *P. aeruginosa*), Both genomes encode a second putative thymidylate kinase (Gmet_3250 = GSU3301) distantly related to all others, in addition to the one found in other *Geobacteraceae *(Gmet_2318 = GSU2229, 41% identical to the *E. coli *enzyme [72]). *G. sulfurreducens *has evidently lost the *purT *gene product of *G. metallireducens *and several other *Geobacteraceae *(Gmet_3193, 58% identical to the *E. coli *enzyme [73]), which incorporates formate directly into purine nucleotides instead of using the folate-dependent *purN *gene product (Gmet_1845 = GSU1759, 46% identical to the *E. coli *enzyme [74]).

### Carbohydrate metabolism

Comparative genomics indicates that, similar to most *Geobacter *species, *G. metallireducens *possesses two glyceraldehyde-3-phosphate dehydrogenase isoenzymes: Gmet_1211 and Gmet_1946 (59% and 56% identical to gluconeogenic GapB and glycolytic GapA of *Corynebacterium glutamicum *[75], respectively), but *G. sulfurreducens *has an ortholog of only the latter (GSU1629). *G. metallireducens *also has a putative fructose 6-kinase (Gmet_2805, 39% identical to the *E. coli *enzyme [76]) that is not present in *G. sulfurreducens*. Remarkably,* G. metallireducens *possesses two isoenzymes each of UDP-glucose 4-epimerase (Gmet_1486; Gmet_2329 = GSU2240, 50% and 54% identical to the *A. brasilense *enzyme [77]), glutamine:fructose-6-phosphate aminotransferase (Gmet_1487; Gmet_0104 = GSU0270, 55% and 53% identical to the *Thermus thermophilus *enzyme [78]), GDP-mannose 4,6-dehydratase (Gmet_1488 = GSU0626; Gmet_1311, 61% and 72% identical to the *E. coli *enzyme [79]) and UDP-*N*-acetylglucosamine 2-epimerase (Gmet_1489 = GSU2243, 61% identical to the *E. coli *enzyme [80]; Gmet_1504, 39% identical to the *Methanococcus maripaludis *enzyme [81]). *G. metallireducens *has evolved a gene cluster of the four enzyme activities (Gmet_1486-Gmet_1489) from both ancestral gene duplication and lateral gene transfer (data not shown). The reason for this emphasis on interconversion of hexoses in *G. metallireducens versus G. sulfurreducens *is unknown.

Unlike the genomes of *G. sulfurreducens *and most other *Geobacteraceae*, which encode the enzymes of only the non-oxidative branch of the pentose phosphate pathway, the *G. metallireducens *genome includes a cluster of oxidative pentose phosphate pathway enzyme genes: 6-phosphogluconolactonase (Gmet_2618, 30% identical to the *Pseudomonas putida *enzyme [82]), glucose-6-phosphate dehydrogenase (Gmet_2619, 50% identical to the *Nostoc punctiforme *enzyme [83]), and 6-phosphogluconate dehydrogenase (Gmet_2620, 36% identical to YqeC of *B. subtilis *[84]), along with two ribose-5-phosphate isomerase isoenzymes (Gmet_2621 and Gmet_1604 = GSU1606, 39% and 44% identical to RpiB of *E. coli *[85]). Thus, *G. metallireducens *apparently generates biosynthetic reducing equivalents in the form of NADPH from carbohydrates. The NADPH supply of *G. sulfurreducens*, in contrast, may derive from the electron transfer chain via a ferredoxin:NADP^+ ^reductase (GSU3058-GSU3057, each 52% identical to its *Pyrococcus furiosus *homolog [86]) that is found in other *Geobacteraceae*, but not in *G. metallireducens*.

Both *G. sulfurreducens *and *G. metallireducens *may protect themselves from desiccation by making trehalose from glucose storage polymers via maltooligose in three steps catalyzed by an alpha-amylase domain protein (Gmet_3469 = GSU2361), maltooligosyltrehalose synthase (Gmet_3468 = GSU2360, 35% identical to the *Rhizobium leguminosarum *enzyme [87]), and maltooligosyltrehalose trehalohydrolase (Gmet_3467 = GSU2358, 44% identical to the *Arthrobacter *strain Q36 enzyme [88]). *G. sulfurreducens*, *P. propionicus *and *G. lovleyi *may also make trehalose from glucose-6-phosphate by the sequential action of trehalose-6-phosphate synthase (GSU2337, containing a domain 37% identical to the *Mycobacterium tuberculosis *enzyme [89]) and trehalose-6-phosphatase (GSU2336, 29% identical to the *E. coli *enzyme [90]), which are missing in *G. metallireducens*. Thus, *G. sulfurreducens *is capable of achieving osmotolerance without consuming carbohydrate storage polymers, but *G. metallireducens *is not.

### Biogenesis of *c*-type cytochromes and pili

The genome of *G. metallireducens *encodes 91 putative *c*-type cytochromes, of which 65 have homologs among the 103 *c*-type cytochromes of *G. sulfurreducens*. Of the *c*-type cytochrome genes implicated in Fe(III) and U(VI) reduction in *G. sulfurreducens*, those conserved in *G. metallireducens *are *macA *(Gmet_3091 = GSU0466) [91-93] and *ppcA *(Gmet_2902 = GSU0612) [37], whereas different *c*-type cytochrome sequences are found in syntenous locations where one would expect *omcB *and *omcC *(Gmet_0910 ≠ GSU2737; Gmet_0913 ≠ GSU2731) [94], and *omcE *(Gmet_2896 ≠ GSU0618) [95]. The *G. metallireducens *genome contains no genes homologous to *omcS *(GSU2504) and *omcT *(GSU2503) [95], and only a paralog (Gmet_0155 = GSU2743) of *omcF *(GSU2432) [96]. This lack of conservation is being investigated further (J. Butler, personal communication).

Notable differences between *G. metallireducens *and *G. sulfurreducens *are apparent in the biogenesis of *c*-type cytochromes, in biosynthesis of the heme group, and in reduction of disulfide bonds to allow covalent linkage to heme. In addition to the membrane-peripheral protoporphyrinogen IX oxidase of *G. sulfurreducens *and other *Geobacteraceae*, encoded by the *hemY *gene (Gmet_3551 = GSU0012, 38% identical to the *Myxococcus xanthus *enzyme [97]), *G. metallireducens *has a membrane-integral isoenzyme encoded by *hemG *(Gmet_2953, 43% identical to the *E. coli *enzyme [98]), with a homolog in *Geobacter *FRC-32. These two species also possess a putative disulfide bond reduction system not found in *G. sulfurreducens *and other *Geobacteraceae*, comprised of DsbA, DsbB, DsbE and DsbD homologs (Gmet_1380, Gmet_1381, Gmet_1383, Gmet_1384), encoded in a cluster alongside a two-component signalling system (Gmet_1378-Gmet_1379), an arylsulfotransferase (Gmet_1382), and a conserved protein of unknown function (Gmet_1385). Transcription of *dsbA *and *dsbB *is diminished during growth on benzoate [21], and phylogenetic analysis indicates that these DsbA and DsbB proteins belong to subfamilies distinct from those that have been characterized (R. Dutton, personal communication). Located apart from this cluster, DsbC/DsbG (Gmet_2250) of *G. metallireducens *has homologs in several *Geobacteraceae*, but not in *G. sulfurreducens*. However, CcdA/DsbD (Gmet_2451 = GSU1322) is present in both. Thus, the pathways of *c*-type cytochrome biogenesis may be significantly different in the two species and somehow linked to the degradation of aromatic compounds by *G. metallireducens*.

In both *G. sulfurreducens *and *G. metallireducens*, there are four *c*-type cytochrome biogenesis genes related to ResB of *B. subtilis *[99], each predicted to be co-transcribed with a gene encoding a ResC/HemX-like protein (hypothesized to be a heme transporter with eight predicted transmembrane segments) [100] and several multiheme *c*-type cytochrome genes (Additional file 10: Table S5). One more protein of the ResC/HemX-like family (Gmet_3232 = GSU3283) is encoded among enzymes of heme biosynthesis in both genomes. These gene arrangements suggest that each pair of *c*-type cytochrome biogenesis proteins may be dedicated to the efficient expression of the cytochromes encoded nearby. Two of the pairs are orthologously conserved (Gmet_2901-Gmet_2900 = GSU0613-GSU0614; Gmet_0592..Gmet_0594 = GSU2891-GSU2890); the other two pairs (Gmet_0572-Gmet_0573; Gmet_0578-Gmet_0579; GSU0704-GSU0705; GSU2881.1-GSU2880), which appear to derive from expansion of ancestral genes, may be relevant to the diversified *c*-type cytochrome repertoire of the two species. Interestingly, three of these gene pairs in *G. metallireducens *are arranged in proximity to each other in a cluster of ten operons with the same coding DNA strand (Gmet_0571 to Gmet_0601), suggesting that their expression may be co-ordinated by transcriptional readthrough (Additional file 10: Table S5). The purposes of various pairs of *c*-type cytochrome biogenesis proteins in *Geobacteraceae *remain to be determined.

The pili of *G. sulfurreducens *have been implicated in electron transfer [101,102] and biofilm formation [103]. Most genes attributed to pilus biogenesis in *G. sulfurreducens *have orthologs in *G. metallireducens*, suggesting that these roles of pili may be conserved. However, instead of the ancestral *pilY1 *gene found in *G. sulfurreducens *(GSU2038) and other *Geobacteraceae*, which may encode a pilus tip-associated adhesive protein [104], *G. metallireducens *possesses a phylogenetically distinct *pilY1 *gene in the same location (Gmet_0967; data not shown), surrounded by different genes of unknown function within a cluster of pilus biogenesis genes. Therefore, it remains possible that structural and functional differences between the pili of the two species will be identified in future.

### Solute transport systems

Although the substrates of most solute transport systems of *G. metallireducens *and *G. sulfurreducens *are unknown, several features distinguish the two species (Additional file 11: Table S6). One of two predicted GTP-dependent Fe(II) transporters of the *Geobacteraceae *(*feoB-1 *Gmet_2444 = GSU1380), located next to the ferric uptake regulator gene (*fur *Gmet_2445 = GSU1379), is present in *G. metallireducens*; the other (*feoB-2 *GSU3268), with two *feoA *genes on its 5' side (GSU3268.1, GSU3270) potentially encoding an essential cytosolic component of the transport system [105], is not. Phylogenetic analysis showed that the FeoB-2 proteins of *Geobacteraceae *are closely related to the characterized Fe(II)-specific FeoB proteins of *Porphyromonas gingivalis *[106] and *Campylobacter jejuni *[107], whereas the FeoB-1 proteins of *Geobacteraceae *cluster apart from them (data not shown). FeoB-1 proteins are not closely related to the manganese-specific FeoB of *P. gingivalis *[106] either, and so their substrate specificity cannot be assigned at present.

In *G. metallireducens*, duplicate *kup *genes, predicted to encode low-affinity potassium/proton symporters, are found in one place (Gmet_0038 = GSU3342; Gmet_0039 = GSU2485, 29% and 31% identical to the *E. coli *protein [108]), apart from the *kdpABCDE *genes (Gmet_2433-Gmet_2437 = GSU2480-GSU2484, 38–49% identical to the homologs in *E. coli *[109,110]) encoding an osmosensitive potassium-translocating ATPase complex. In *G. sulfurreducens*, one of these *kup *genes (GSU2485) is located 3' of the *kdp *gene cluster, apparently under control of an osmosensitive riboswitch (GSU2484.1, sequence coordinates 2728254 to 2728393), and there is a third *kup *gene (GSU2350, 49% identity to *E. coli*) not found in other *Geobacteraceae*. *G. sulfurreducens *also has at least two potassium/proton antiporters (GSU1203, 34% identical to CvrA of *Vibrio parahaemolyticus *[111]; GSU2759, 31% identical to KefB of *E. coli *[112]) and a sodium/proton antiporter complex (*mrpABCDEFG *GSU2344-GSU2338, 29–48% identical to the homologs in *B. subtilis *[113]) that are not found in *G. metallireducens*. Three mechanosensitive ion channels are common to the two species (Gmet_1942 = GSU1633; Gmet_2581 = GSU2316; and Gmet_2522 = GSU2794); two more are unique to *G. sulfurreducens *(GSU1557; GSU1723). Thus, control of monovalent cation homeostasis appears to be more complex in *G. sulfurreducens*.

Several heavy metal efflux pumps are conserved between the two species, but their substrate specificity is uncertain. Transporters present in *G. sulfurreducens *but not *G. metallireducens *include that for uracil (GSU0932, 48% identical to the *Bacillus caldolyticus *protein [114]). Transporters present in *G. metallireducens *but not *G. sulfurreducens *include those for nitrate/nitrite (Gmet_0333-Gmet_0334) and chromate (Gmet_2732-Gmet_2731), which are each present as two paralogous genes rather than gene fusions such as their homologs that have been characterized in other bacteria [36,115].

### Signalling, chemotaxis and global regulation

*G. metallireducens *possesses orthologs of the six sigma factors of RNA polymerase identified in *G. sulfurreducens *(Table 3), as well as a seventh factor (Gmet_2792) not found in other *Geobacteraceae*, related to the extracytoplasmic sigma-Z factor of *B. subtilis *[116]. Intriguingly, a particular anti-anti-sigma factor gene is frameshifted in both genomes: GSU1427 has frameshifts in the phosphatase domain, resulting in an in-frame protein, whereas the homologous Gmet_1229 is shifted out of frame in the kinase domain. These differences imply that global regulatory networks may be different in the two species.

**Table 3:** Sigma factors of *G. metallireducens *and *G. sulfurreducens*.

Locus Tag	Annotation	*G. metallireducens *gene	*G. sulfurreducens *gene
*rpoH*	RNA polymerase sigma-32 factor	Gmet_2854	GSU0655
*rpoE*	RNA polymerase sigma-24 factor, putative	Gmet_2612	GSU0721
*rpoN*	RNA polymerase sigma-54 factor	Gmet_1283	GSU1887
*rpoD*	RNA polymerase sigma-70 factor RpoD	Gmet_0395	GSU3089
*rpoS*	RNA polymerase sigma-38 factor, stationary phase	Gmet_1421	GSU1525
*fliA*	RNA polymerase sigma-28 factor for flagellar operon	Gmet_0429	GSU3053
none	RNA polymerase sigma-Z factor	Gmet_2792	none

The *G. metallireducens *genome encodes 83 putative sensor histidine kinases containing HATPase_c domains (Additional file 12: Table S7), of which 45 (54%) have orthologs among the 95 such proteins of *G. sulfurreducens*. There are 94 proteins with response receiver (REC) domains in *G. metallireducens *(Additional file 12: Table S7), out of which 66 (70%) have orthologs among the 110 such proteins of *G. sulfurreducens*. Twenty-seven of the REC domain-containing proteins and another 101 genes and four pseudogenes (Additional file 12: Table S7) were predicted to be transcriptional regulators in *G. metallireducens*. There are 20 putative diguanylate cyclases containing GGDEF domains, of which 16 (80%) have orthologs among the 29 putative diguanylate cyclases of *G. sulfurreducens *(Additional file 13: Table S8). Overall, the portion of the genome dedicated to signalling and transcriptional regulation in *G. metallireducens *is slightly less than in *G. sulfurreducens*, but still considerable and significantly different in content.

Several protein factors involved in chemotaxis-type signalling pathways are conserved between the two genomes: *G. sulfurreducens *and *G. metallireducens *each possess four or five CheA sensor kinases and ten CheY response receivers, almost all of which are orthologous pairs (Additional file 14: Table S9). In contrast, 17 of the 34 methyl-accepting chemotaxis proteins (MCPs) of *G. sulfurreducens *have no full-length matches in *G. metallireducens *(Additional file 14: Table S9). Due to apparent gene family expansion in *G. sulfurreducens*, its remaining 17 MCPs correspond to only 13 MCPs of *G. metallireducens *(Additional file 14: Table S9). The other five MCPs of *G. metallireducens *lack full-length matches in other *Geobacteraceae *(Additional file 14: Table S9). Whereas *G. sulfurreducens *may use its closely related MCPs to fine-tune its chemotactic responses, *G. metallireducens *may accomplish response modulation by having twice as many MCP methyltransferases (CheR) and methylesterases (CheB) as *G. sulfurreducens *(Additional file 14: Table S9).

Integration host factors (IHF) and histone-like (HU) DNA-binding proteins are global regulators of gene expression composed of two homologous proteins that bend DNA in specific locations [117]. IHF/HU binding sites are favoured by some mobile genetic elements for insertion. The genome of *G. metallireducens *encodes orthologs of the single HU protein, both IHF beta proteins, and one of two IHF alpha proteins of *G. sulfurreducens *(Table 4). Another HU gene and two additional IHF alpha genes are present in *G. metallireducens *but not *G. sulfurreducens *(Table 4). Three sets of putative global regulatory elements unique to the *G. metallireducens *genome (Additional files 7,8,9: Figures S3, S4 and S5, Additional file 5: Table S4) may be recognized by different combinations of IHF/HU proteins. A fourth set found in *G. metallireducens *(Additional file 15: Figure S6, Additional file 5: Table S4) is similar to multicopy sequences in many other genomes. Two transposons (IS*Gme8 *and IS*Gme9*) were found inserted near putative IHF/HU-binding sites of Class 1 (Additional file 5: Table S4). No such putative global regulatory sequence elements were identified in *G. sulfurreducens*. However, pirin, a Fe(II)-binding protein that associates with DNA in eukaryotic nuclei [118,119], is present in *G. sulfurreducens *as GSU0825, but in *G. metallireducens *only as a frameshifted fragment, Gmet_3471. These genetic differences indicate that the proteins that decorate and bend the chromosome are very different in the two species.

**Table 4:** Integration host factor (IHF) and histone-like (HU) genes of *G. metallireducens *and *G. sulfurreducens*.

Locus Tag	*G. metallireducens *gene	*G. sulfurreducens *gene
*ihfA-1*	Gmet_1417	GSU1521
*ihfA-2*	none	GSU2120
*ihfA-3*	Gmet_3057	none
*ihfA-4*	Gmet_3056*	none
*ihfB-1*	Gmet_1833	GSU1746
*ihfB-2*	Gmet_0868	GSU2602
*hup-1*	Gmet_0355	GSU3132
*hup-2*	Gmet_1608	none

*Gmet_3056 is frameshifted near the N-terminus, but may be expressed from an internal start codon.

The functions and associations of the various IHF alpha (*ihfA*), IHF beta (*ihfB*), and HU (*hup*) genes are yet unknown, as is their correspondence to any of the predicted regulatory sites illustrated in Figures S3, S4, S5, and S6.

Although no quorum sensing through *N*-acylhomoserine lactones (autoinducers) has ever been demonstrated for any *Geobacteraceae*, this kind of signalling may be possible for *G. metallireducens *because it possesses a LuxR family transcriptional regulator with an autoinducer-binding domain (Gmet_1513), and two divergently transcribed genes with weak sequence similarity to autoinducer synthetases (Gmet_2037 and Gmet_2038). Both Gmet_2037 and Gmet_2038 have atypically low G+C content (Additional file 1: Table S1) and may have been recently acquired by *G. metallireducens*. The presence of a conserved nucleotide sequence on the 5' side of Gmet_2037 and in 15 other locations on the chromosome (Additional file 16: Figure S7, Additional file 5: Table S4) suggests that Gmet_2037 may be an unusual autoinducer synthetase that is regulated by a riboswitch rather than an autoinducer-binding protein. This conserved sequence is also found on the 5' side of many genes (frequently *c*-type cytochromes) in the genomes of *G. sulfurreducens*, *G. uraniireducens*, and *P. propionicus*, and overlaps with predicted cyclic diguanylate-responsive riboswitches [120].

The genomes of *G. metallireducens *and *G. sulfurreducens *differ in several other aspects of regulation. Nine pairs of potential toxins and antitoxins were identified in the *G. metallireducens *genome (Additional file 17: Table S10), which may poison vital cellular processes in response to stimuli that interfere with their autoregulation. Only one of these was similar to one of the five potential toxin/antitoxin pairs of *G. sulfurreducens*. Both the CRISPR1 and CRISPR2 (clustered regularly interspaced short palindromic repeat) loci of *G. sulfurreducens*, thought to encode 181 short RNAs that may provide immunity against infection by unidentified phage and plasmids [121,122], have no parallel in *G. metallireducens*, which has CRISPR3 (also found in *G. uraniireducens*) instead, encoding only twelve putative short RNAs of more variable length and unknown target specificity (Additional file 18: Table S11). Another difference in RNA-level regulation is that a single-stranded RNA-specific nuclease of the barnase family (Gmet_2616) and its putative cognate inhibitor of the barstar family (Gmet_2617) are present in *G. metallireducens *but not *G. sulfurreducens*.

Several conserved nucleotide sequences were identified by comparison of intergenic regions between the *G. sulfurreducens *and *G. metallireducens *genomes, and those that are found in multiple copies (Additional file 19: Figure S8, Additional file 5: Table S4) may give rise to short RNAs with various regulatory or catalytic activities.

## Conclusion

Inspection of the *G. metallireducens *genome indicates that this species has many metabolic capabilities not present in *G. sulfurreducens*, particularly with respect to the metabolism of organic acids. Many biosynthetic pathways and regulatory features are conserved, but several putative global regulator-binding sites are unique to *G. metallireducens*. The complement of signalling proteins is significantly different between the two genomes. Thus, the genome of *G. metallireducens *provides valuable information about conserved and variable aspects of metabolism, physiology and genetics of the *Geobacteraceae*.

## Methods

### Sequence analysis and annotation

The genome of *G. metallireducens *GS-15 [31] was sequenced by the Joint Genome Institute from cosmid and fosmid libraries. Two gene modeling programs – Critica (v1.05), and Glimmer (v2.13) – were run on both replicons [GenBank:NC007517, GenBank:NC007515], using default settings that permit overlapping genes and using ATG, GTG, and TTG as potential starts. The results were combined, and a BLASTP search of the translations *vs*. Genbank's non-redundant database (NR) was conducted. The alignment of the N-terminus of each gene model *vs*. the best NR match was used to pick a preferred gene model. If no BLAST match was returned, the longest model was retained. Gene models that overlapped by greater than 10% of their length were flagged for revision or deletion, giving preference to genes with a BLAST match. The revised gene/protein set was searched against the Swiss-Prot/TrEMBL, PRIAM, Pfam, TIGRFam, Interpro, KEGG, and COGs databases, in addition to BLASTP *vs*. NR. From these results, product assignments were made. Initial criteria for automated functional assignment set priority based on PRIAM, TIGRFam, Pfam, Interpro profiles, pairwise BLAST *vs*. Swiss-Prot/TrEMBL, KEGG, and COG groups. tRNAs were annotated using tRNAscan-SE (v1.23). rRNAs were annotated using a combination of BLASTN and an rRNA-specific database. The srpRNA was located using the SRPscan website. The *rnpB *and tmRNA were located using the Rfam database and Infernal. Riboswitches and other noncoding RNAs predicted in the *G. sulfurreducens *genome [GenBank:NC00293] were retrieved from the Rfam database [123] and used to annotate the corresponding sequences in *G. metallireducens*.

Operon organization was predicted using the commercial version of the FGENESB software (V. Solovyev and A. Salamov, unpublished; Softberry, Inc; 2003–2007), with sequence parameters estimated separately from the *G. sulfurreducens *and *G. metallireducens *genomes. Default parameters were used in operon prediction, including minimum ORF length of 100 bp.

Binding sites of the global regulator ModE (consensus ATCGCTATATANNNNNNTATATAACGAT) were predicted using ScanACE software [41,42] using the algorithm of Berg and von Hippel [124] and the footprinted matrix of *E. coli *ModE-regulated sites from the Regulon DB database v 4.0 [125]. Functional annotations of transport proteins were evaluated by referring to TCDB http://www.tcdb.org, and PORES http://garlic.mefos.hr/pores was used to annotate porins. Transposase families were assigned IS*Gme *numbers for inclusion in the ISFinder database http://www-is.biotoul.fr.

### Manual curation

The automated genome annotation of *G. metallireducens *was queried with the protein BLAST algorithm [126] using all predicted proteins in the automated annotation of the *G. sulfurreducens *genome [12] to identify conserved genes that aligned over their full lengths. The coordinates of numerous genes in both genomes were adjusted according to the criteria of full-length alignment, plausible ribosome-binding sites, and minimal overlap between genes on opposite DNA strands. The annotations of all other genes in *G. metallireducens *were checked by BLAST searches of NR. Discrepancies in functional annotation of conserved genes between the two genomes were also resolved by BLAST of NR and of the Swiss-Prot database. All hypothetical proteins were checked for similarity to previously identified domains, conservation among other *Geobacteraceae*, and absence from species other than *Geobacteraceae*. Genes that had no protein-level homologs in NR were checked (together with flanking intergenic sequences) by translated nucleotide BLAST in all six reading frames, and by nucleotide BLAST to ensure that conserved protein-coding or nucleotide features had not been missed. All intergenic regions of 120 bp or larger were also checked, which led to the annotation of numerous conserved nucleotide sequences numbered as follows: Gmet_R#### (for predicted RNAs and miscellaneous conserved sequences, a nonzero first digit indicating membership in a group of four or more sequences); Gmet_P#### (for conserved, putative regulatory sequences 5' of predicted operons, numbers corresponding to the first gene of the operon); Gmet_I[1-4]## [A, B] (for the four classes of putative global regulator binding sites, mostly found in pairs); Gmet_H4## (for putative global regulatory elements consisting of four tandem heptanucleotide repeats); and Gmet_C### (for the spacers of clustered regularly interspaced short palindromic repeats – CRISPR). Newly added features in the *G. sulfurreducens *genome were assigned unique numbers with decimal points (GSU####.#) in accordance with earlier corrections.

### Phylogenetic analysis

Phylogenetic analysis of selected proteins was performed on alignments generated using T-COFFEE [127], manually corrected in Mesquite [128]. Phylogenetic trees were constructed by the neighbour-joining method using Phylip software [129], with 500 bootstrap replications.

## Abbreviations

ATP: adenosine triphosphate; CoA: coenzyme A; CRISPR: clustered regularly interspaced short palindromic repeats; DAHP: 3-deoxy-D-arabino-heptulosonate-7-phosphate; DNA: deoxyribonucleic acid; GDP: guanosine diphosphate; GTP: guanosine triphosphate; HAD: haloacid dehalogenase; HU: histone-like DNA-binding proteins; IHF: integration host factors; MCP: methyl-accepting chemotaxis protein; NAD(H): nicotinamide adenine dinucleotide (reduced); NADP(H): nicotinamide adenine dinucleotide phosphate (reduced); RNA: ribonucleic acid; SAM: *S*-adenosylmethionine; TCA: tricarboxylic acid; tRNA: transfer RNA; UDP: uridine diphosphate

## Authors' contributions

AL supervised the genome sequencing, GD performed genome sequence finishing, and ML supervised the automated annotation process. JK predicted ModE binding sites. MA performed manual curation of the genome annotations, sequence alignments and phylogenetic analyses, and wrote the manuscript. DL conceived of the study and offered guidance with the writing. All authors read, assisted with editing, and approved the final manuscript.

## Supplementary Material

Additional File 1 **Table S1. Genes of *G. metallireducens *with atypical G+C content (more than two standard deviations from the mean)**. This table lists genes of *G. metallireducens *that have G+C content more than two standard deviations from the mean, and indicates by shading (alternated for contrast) those gene clusters that may be recent acquisitions. Click here for file

Additional File 2 **Table S2. Enzymes of acyl-CoA metabolism in *G. sulfurreducens *and *G. metallireducens***. This table compares the genes predicted to function in acyl-CoA metabolism in *G. sulfurreducens *and *G. metallireducens*. Click here for file

Additional File 3 **Table S3. Predicted binding sites of the global regulator ModE in the genome of *G. sulfurreducens*, which are mostly absent from the *G. metallireducens *genome**. This table lists the predicted ModE-binding sites of *G. sulfurreducens *and compares them to the corresponding sequences in *G. metallireducens*. Click here for file

Additional File 4 **Figure S1. A family of 24 predicted short RNA elements in the *G. metallireducens *genome**. This is an alignment of 24 DNA sequences that were matched by nucleotide-level BLAST. Each RNA is found in an intergenic region, e.g. the 5' regions of genes affecting lysine/arginine metabolism, and contains a central palindromic structure GRCGTAGCGCTGCTACGCC. Similar sequences were found in the genomes of *G. sulfurreducens*, *G. uraniireducens*, and *Desulfotalea psychrophila*. The sequence strand and start and stop nucleotide positions are indicated. Click here for file

Additional File 5 **Table S4. Genes found next to multicopy nucleotide sequences of unknown function in *G. metallireducens***. This table lists the genes adjacent to all of the multicopy nucleotide sequences identified in the *G. metallireducens *genome. Click here for file

Additional File 6 **Figure S2. A family of 49 predicted regulatory RNA elements in *G. metallireducens*, containing four heptanucleotide repeats (consensus GGACCGG)**. This is an alignment of 49 DNA sequences that were matched by nucleotide-level BLAST. These elements are found within genes, sometimes more than once per gene, as well as between genes. The sequence strand and start and stop nucleotide positions are indicated. Click here for file

Additional File 7 **Figure S3. Predicted global regulator binding sites (class 1)**. This is an alignment of 48 DNA sequences that were matched by nucleotide-level BLAST. Each site contains four tandem octanucleotide repeats (consensus GTTGCTYN), the outer two being poorly conserved. The distance between each pair of sites (on opposite strands) is variable. Each sequence begins at the right extremity of the top line (the 3' side of the "-" strand of the chromosome), loops on the left side (switching strands), and continues to the right extremity of the bottom line (the 3' side of the "+" strand of the chromosome); start and stop nucleotide positions are indicated. Insertion sequences of the IS*Gme8 *or IS*Gme9 *families may be found at a fixed distance from either or both sites of a pair; these occurrences are indicated on the appropriate lines. Click here for file

Additional File 8 **Figure S4. Predicted global regulator binding sites (class 2)**. This is an alignment of 47 DNA sequences that were matched by nucleotide-level BLAST. Each of 21 paired sites, four sites that also belong to class 1, and one possibly vestigial unpaired site contains three tandem repeats (consensus TCTCCGTS[Y]). The distance between each pair of sites (on opposite strands) is variable. Each sequence begins at the right extremity of the top line (the 3' side of the "-" strand of the chromosome), loops on the left side (switching strands), and continues to the right extremity of the bottom line (the 3' side of the "+" strand of the chromosome); start and stop nucleotide positions are indicated. Click here for file

Additional File 9 **Figure S5. Predicted global regulator binding sites (class 3)**. This is an alignment of 16 DNA sequences that were matched by nucleotide-level BLAST. Fifteen of the sites consist of five tandem heptanucleotide repeats (consensus MTYCTGA). Each sequence begins at the right extremity of the top line (the 3' side of the "-" strand of the chromosome), loops on the left side (switching strands), and continues to the right extremity of the bottom line (the 3' side of the "+" strand of the chromosome); start and stop nucleotide positions are indicated. Click here for file

Additional File 10 **Table S5. Cytochrome *c *biogenesis gene clusters of *G. sulfurreducens *and *G. metallireducens*, and associated *c*-type cytochromes**. This table compares the clusters of genes predicted to be involved in biogenesis of *c*-type cytochromes in *G. sulfurreducens *and *G. metallireducens*. Click here for file

Additional File 11 **Table S6. Transport systems of *G. sulfurreducens *and *G. metallireducens***. This table compares the genes predicted to be involved in transport of solutes across the cell membrane and cell wall of *G. sulfurreducens *and *G. metallireducens*. Click here for file

Additional File 12 **Table S7. Sensor histidine kinases (HATPase_c domain proteins), REC domain-containing proteins, and transcriptional regulators of *G. metallireducens***. This table compares the genes predicted to be involved in two-component signalling and transcriptional regulation in *G. sulfurreducens *and *G. metallireducens*. Click here for file

Additional File 13 **Table S8. Diguanylate cyclases (GGDEF domain proteins) of *G. sulfurreducens *and *G. metallireducens***. This table compares the genes predicted to produce the intracellular messenger cyclic diguanylate in *G. sulfurreducens *and *G. metallireducens*. Click here for file

Additional File 14 **Table S9. Chemotaxis-type signalling proteins of *G. sulfurreducens *and *G. metallireducens***. This table compares the genes predicted to participate in chemotaxis-type signalling in *G. sulfurreducens *and *G. metallireducens*. Click here for file

Additional File 15 **Figure S6. Predicted global regulator binding sites (class 4)**. This is an alignment of 20 DNA sequences that were matched by nucleotide-level BLAST. Each site appears to be based on a pentanucleotide repeat (consensus CCYTC) that occurs four times on one strand and twice on the other. The sequence strand and start and stop nucleotide positions are indicated. Click here for file

Additional File 16 **Figure S7. A predicted regulatory short RNA found in the 5' regions of *c*-type cytochromes and other proteins**. This is an alignment of 16 DNA sequences that were matched by nucleotide-level BLAST. The location of Gmet_R3013 suggests that *N*-acylhomoserine lactone signalling may be under control of this RNA element. Similar sequences were found in the genomes of *G. sulfurreducens*, *G. uraniireducens*, and *P. propionicus*. The sequence strand and start and stop nucleotide positions are indicated. Click here for file

Additional File 17 **Table S10. Toxin/antitoxin pairs of *G. metallireducens *and *G. sulfurreducens***. This table compares the genes predicted to encode toxin/antitoxin pairs in *G. sulfurreducens *and *G. metallireducens*. Click here for file

Additional File 18 **Table S11. The CRISPR3 locus of *G. metallireducens *contains spacers of variable length**. The thirteen clustered regularly interspaced short palindromic repeats (CRISPR) of *G. metallireducens *(consensus sequence GTAGCGCCCGCCTACATAGGCGGGCGAGGATTGAAAC) are far fewer than the thirty-eight of CRISPR1 and one hundred and forty-three of CRISPR2 in *G. sulfurreducens*. Click here for file

Additional File 19 **Figure S8. Miscellaneous multicopy nucleotide sequences found in the *G. metallireducens *genome**. These are alignments of 16 sets of miscellaneous DNA sequences in *G. metallireducens *that were matched by nucleotide-level BLAST. The sequence strand and start and stop nucleotide positions are indicated. (a) A palindromic sequence-containing family also found in the genomes of *G. sulfurreducens*, *G. uraniireducens*, and *P. propionicus*. (b) Sequences of this type were also found in the genomes of *G. sulfurreducens *and *G. uraniireducens*. (c) These sequences are unique to *G. metallireducens*. (d) The ends of these sequences form inverted repeats. Each sequence begins at the left extremity of the top line (the 5' side of the "+" strand of the chromosome), loops on the right side (switching strands), and continues to the left extremity of the bottom line (the 5' side of the "-" strand of the chromosome). A fragment related to Gmet_R6002 was found in the *G. sulfurreducens *genome. (e) These sequences are unique to *G. metallireducens*. (f) Sequences of this type were also found in the genomes of *G. uraniireducens *and *G. bemidjiensis*. (g) These sequences contain four octanucleotide repeats (consensus TWGTTGAY), two in tandem on each strand. (h) Sequences of this type were also found in the genome of *G. sulfurreducens*. (i) These sequences are unique to *G. metallireducens*. (j) These elements are located near each other. (k) These sequences are unique to *G. metallireducens*. (l-p) These elements are located near each other. Gmet_R0147 continues as Gmet_R0055, a tRNA-Asn gene (underlined). Click here for file
