# Supplementary material for: The genome sequence of Geobacter metallireducens: features of metabolism, physiology and regulation common and dissimilar to Geobacter sulfurreducens
Source: BMC Microbiol. 2009 May 27;9:109. doi: 10.1186/1471-2180-9-109 (PMC2700814; doi:10.1186/1471-2180-9-109)
Supplement: Additional File 1 — Table S1. Genes of G. metallireducens with atypical G+C content (more than two standard deviations from the mean). This table lists genes of G. metallireducens that have G+C content more than two standard deviations from the mean, and indicates by shading (alternated for contrast) those gene clusters that may be recent acquisitions. [file 1471-2180-9-109-S1.pdf]

Table S1. Genes of *G. metallireducens* with atypical G+C content (more than two standard deviations from the mean).

Gene	G+C content	Homolog in *G. sulfurreducens*	Functional annotation
**Genes with low G+C content[1]**			
Gmet_0079	45.6%	none	hypothetical protein
Gmet_0080	41.5%	none	conserved hypothetical protein, C-terminal fragment
Gmet_0082	40.3%	none	hypothetical protein
Gmet_3579	45.8%	none	hypothetical protein
Gmet_0284	38.2%	none	hypothetical protein
Gmet_0285	44.6%	none	putative peptidoglycan-binding domain protein
Gmet_0289	45.3%	GSU3170	conserved hypothetical protein
Gmet_0318	41.8%	none	conserved hypothetical protein
Gmet_0322	44.8%	none	hypothetical protein
Gmet_0444	47.5%	GSU3037	flagellar filament cap protein FliD
Gmet_0445	45.6%	GSU3036	flagellin export facilitator protein FliS
Gmet_0612	44.0%	GSU2870	ribosomal protein L33
Gmet_0625	46.3%	GSU2858	ribosomal protein S10
Gmet_0628	38.2%	GSU2855	ribosomal protein L23
Gmet_0630	44.0%	GSU2853	ribosomal protein S19
Gmet_0632	47.5%	GSU2851	ribosomal protein S3
Gmet_0634	40.7%	GSU2849	ribosomal protein L29
Gmet_0635	43.8%	GSU2848	ribosomal protein S17
Gmet_0636	44.7%	GSU2847	ribosomal protein L14
Gmet_0637	45.3%	GSU2846	ribosomal protein L24
Gmet_0638	45.0%	GSU2845	ribosomal protein L5
Gmet_0640	46.9%	GSU2843	ribosomal protein S8
Gmet_0641	47.6%	GSU2842	ribosomal protein L6
Gmet_0644	45.0%	GSU2839	ribosomal protein L30
Gmet_0649	39.5%	GSU2834.1	ribosomal protein L36
Gmet_3592	43.5%	none	hypothetical protein
Gmet_1000	44.0%	none	haloacid dehalogenase-like hydrolase
Gmet_1001	41.1%	none	hypothetical protein
Gmet_1003	42.4%	none	hypothetical protein
Gmet_1114	40.5%	none	conserved hypothetical protein
Gmet_1207	46.2%	none	conserved hypothetical

			protein
Gmet_1316	47.0%	none	nucleotidyltransferase-related protein
Gmet_1317	44.1%	none	protein of unknown function DUF86
Gmet_1320	42.7%	none	nucleotidyltransferase-related protein
Gmet_1321	45.5%	none	conserved hypothetical protein
Gmet_1325	46.1%	none	predicted pyridoxal phosphate-dependent aminotransferase involved in regulation of cell wall biogenesis
Gmet_1326	40.9%	none	lipopolysaccharide biosynthesis protein, putative
Gmet_1327	29.2%	none	glycosyl transferase
Gmet_1328	40.0%	none	glycosyl transferase, family 11
Gmet_1329	36.3%	none	glycosyl transferase, putative
Gmet_1330	29.3%	none	conserved hypothetical protein
Gmet_1331	32.7%	none	glycosyl transferase, putative
Gmet_1332	33.2%	none	glycosyl transferase, putative
Gmet_1334	38.1%	none	glycosyl transferase, putative
Gmet_1361	47.7%	GSU1467	2-oxoglutarate:ferredoxin oxidoreductase, ferredoxin subunit
Gmet_1399	44.8%	GSU1496	geopilin
Gmet_1400	45.9%	GSU1497	conserved hypothetical protein
Gmet_1401	47.7%	GSU1499	membrane protein, putative
Gmet_1404	42.9%	GSU1498	membrane protein, putative
Gmet_1406	38.5%	GSU1504	ABC transporter, membrane protein, ABC-2 family
Gmet_1407	43.7%	GSU1505	ABC transporter, ATP-binding protein
Gmet_1408	45.5%	none	glycosyl transferase, group 1
Gmet_1409	40.9%	none	hypothetical protein
Gmet_1417	47.5%	GSU1521	integration host factor, alpha subunit
Gmet_1418	47.3%	GSU1522	transcriptional regulator, MerR family
Gmet_1494	41.6%	none	hypothetical protein
Gmet_1495	46.0%	none	glycosyl transferase, group 1,

			putative
Gmet_1496	41.5%	none	glycosyl transferase, group 1
Gmet_1497	45.5%	none	glycosyl transferase, putative
Gmet_1498	38.8%	none	glycosyl transferase, group 1
Gmet_1540	44.8%	none	benzylsuccinate synthase gamma subunit
Gmet_1557	35.6%	none	hypothetical protein
Gmet_1702	43.0%	none	hypothetical protein
Gmet_1786	44.9%	none	hypothetical protein
Gmet_3606	44.2%	GSU1796.1	conserved hypothetical protein
Gmet_1951	47.6%	GSU1824	conserved hypothetical protein
Gmet_2010	41.4%	GSU1962	glycosyl transferase
Gmet_2011	39.5%	none	conserved hypothetical protein
Gmet_2012	39.9%	none	O-antigen/teichoic acid export membrane protein
Gmet_2014	46.7%	GSU1961	glycosyl transferase, family 2
Gmet_2015	42.4%	none	rhodanese-related domain selenoprotein, putative
Gmet_2017	46.8%	none	cellulose biosynthesis-like protein
Gmet_2033	46.8%	GSU1988	conserved hypothetical protein
Gmet_2037	45.9%	none	*N*-acylhomoserine lactone synthetase-related protein
Gmet_2038	43.7%	none	*N*-acylhomoserine lactone synthetase-related protein
Gmet_2040	46.5%	none	2-oxoacid dehydrogenase acyltransferase protein
Gmet_2041	45.7%	none	glycosyl transferase, family 28-related
Gmet_2042	46.7%	none	conserved hypothetical protein
Gmet_2089	46.4%	GSU2468	conserved hypothetical protein
Gmet_2090	39.2%	none	hypothetical protein
Gmet_2092	39.0%	none	conserved hypothetical protein
Gmet_2179	40.9%	none	glycosyl transferase, putative
Gmet_2180	35.9%	none	methyltransferase, putative
Gmet_2181	39.8%	none	polysaccharide biosynthesis integral membrane protein
Gmet_2216	43.7%	none	transcriptional regulator,

			TetR family
Gmet_2228	46.9%	none	thioesterase, putative
Gmet_2232	44.3%	none	hypothetical protein
Gmet_2237	45.8%	none	conserved hypothetical protein
Gmet_2241	36.3%	none	transcriptional regulator, TetR family
Gmet_2242	40.5%	none	conserved hypothetical protein
Gmet_2243	44.7%	none	conserved hypothetical protein
Gmet_2244	46.0%	none	predicted exporter, RND superfamily
Gmet_2246	47.2%	none	AMP-forming acyl-CoA synthetase
Gmet_2254	46.1%	none	ferritin-like domain protein
Gmet_3613	47.0%	none	hypothetical protein
Gmet_2259	47.1%	none	transcriptional regulator, IclR family
Gmet_2274	33.2%	none	conserved hypothetical protein
Gmet_2275	47.6%	none	conserved hypothetical protein
Gmet_2280	36.0%	none	hypothetical protein
Gmet_2334	33.4%	none	glycosyl transferase, family 8
Gmet_2335	34.6%	none	conserved hypothetical protein
Gmet_2449	46.5%	GSU1323.1	conserved hypothetical protein
Gmet_2486	40.8%	none	hypothetical protein
Gmet_2490	41.7%	none	conserved hypothetical protein
Gmet_2500	44.6%	none	conserved hypothetical protein
Gmet_2535	42.1%	none	hypothetical protein
Gmet_2540	40.0%	none	histidine kinase-like ATPase (HATPase_c)
Gmet_2589	38.6%	none	conserved hypothetical protein
Gmet_2590	41.9%	none	hypothetical protein
Gmet_3623	47.5%	none	hypothetical protein
Gmet_2754	37.8%	none	hypothetical protein
Gmet_2755	36.6%	none	hypothetical protein
Gmet_2795	45.3%	none	hypothetical protein
Gmet_2798	46.3%	none	hypothetical protein

Gmet_2799	46.2%	none	hypothetical protein
Gmet_2800	39.0%	none	hypothetical protein
Gmet_2801	39.2%	none	conserved hypothetical protein
Gmet_2885	39.5%	none	glycosyl transferase
Gmet_2886	47.1%	none	SAM-dependent methyltransferase, putative
Gmet_2887	45.7%	none	SAM-dependent methyltransferase, putative
Gmet_2963	47.5%	none	hypothetical protein
Gmet_2965	42.6%	none	conserved hypothetical protein
Gmet_2966	41.7%	none	hypothetical protein
Gmet_2967	43.0%	none	conserved hypothetical protein
Gmet_3002	46.7%	none	hypothetical protein
Gmet_3005	47.5%	none	hypothetical protein
Gmet_3051	43.7%	none	hypothetical protein
Gmet_3052	42.5%	none	predicted serine protease with TPR domains
Gmet_3057	47.3%	none	integration host factor subunit, alpha subunit
Gmet_3629	47.5%	none	hypothetical protein
Gmet_3135	38.9%	none	conserved hypothetical protein
Gmet_3630	38.9%	none	conserved hypothetical protein
Gmet_3631	29.8%	none	hypothetical protein
Gmet_3632	42.0%	none	hypothetical protein
Gmet_3139	35.4%	none	hypothetical protein
Gmet_3634	34.8%	none	hypothetical protein
Gmet_3142	41.2%	none	resolvase-like protein
Gmet_3143	37.2%	none	resolvase-like protein
Gmet_3252	44.8%	none	conserved hypothetical protein
Gmet_3345	42.9%	GSU0348	NADH dehydrogenase I, K subunit
Gmet_3347	45.7%	GSU0346	NADH dehydrogenase I, I subunit
Gmet_3355	47.6%	GSU0338	NADH dehydrogenase I, A subunit
Gmet_A3565	46.0%	none	RepA protein
Gmet_A3566	35.8%	none	hypothetical protein
Gmet_A3576	44.1%	none	hypothetical protein
Gmet_A3577	45.2%	none	hypothetical protein

Genes with high G+C content			
Gmet_2633	71.7%	GSU1216	cytochrome *bd* ubiquinol oxidase assembly ATP-binding protein CydC
Gmet_2634	71.1%	GSU1215	cytochrome *bd* ubiquinol oxidase assembly ATP-binding protein CydD
Gmet_2809	71.4%	none	protein of unknown function DUF490
Gmet_3397	73.4%	none	hypothetical protein

[1]Shading (alternated for clarity) indicates gene clusters that may have been acquired by *G. metallireducens* by lateral gene transfer.
