# Supplementary material for: The genome sequence of Geobacter metallireducens: features of metabolism, physiology and regulation common and dissimilar to Geobacter sulfurreducens
Source: BMC Microbiol. 2009 May 27;9:109. doi: 10.1186/1471-2180-9-109 (PMC2700814; doi:10.1186/1471-2180-9-109)
Supplement: Additional File 2 — Table S2. Enzymes of acyl-CoA metabolism in G. sulfurreducens and G. metallireducens. This table compares the genes predicted to function in acyl-CoA metabolism in G. sulfurreducens and G. metallireducens. [file 1471-2180-9-109-S2.pdf]

Table S2. Enzymes of acyl-CoA metabolism in *G. sulfurreducens* and *G. metallireducens*.

***G. metallireducens* gene(s)**	***G. sulfurreducens* gene(s)**	**Function**
**Acyl-CoA:carboxylate CoA-transferases**		
Gmet_1125	no ortholog	succinyl:propionate CoA-transferase?
Gmet_1730	GSU0174	succinyl:acetate CoA-transferase
Gmet_3044	GSU0490	succinyl:acetate CoA-transferase
Gmet_2054	no ortholog	acyl-CoA:carboxylate CoA-transferase, putative
Gmet_3294	no ortholog	acyl-CoA:carboxylate CoA-transferase, putative
Gmet_3304	no ortholog	acyl-CoA:carboxylate CoA-transferase, putative
Gmet_2142	no ortholog	acetyl-CoA hydrolase/transferase
Gmet_1521-Gmet_1522	no ortholog	benzylsuccinate CoA-transferase subunits
Gmet_3296-Gmet_3297	no ortholog	acyl-CoA:acetate/3-oxoacid CoA-transferase, subunits
Gmet_1709-Gmet_1708	no ortholog	CoA transferase, alpha and beta subunits
Gmet_1711	no ortholog	CoA transferase, family III
Gmet_2205	no ortholog	CoA transferase
Gmet_3657	no ortholog	CoA transferase, family III fragment
**Acyl-CoA synthetases**		
Gmet_0086	GSU3029	lipid biosynthesis (fusion with acyltransferase)
Gmet_1613	GSU1677	lipid biosynthesis (fusion with acyl carrier protein and acyltransferase)
Gmet_0950	GSU2051	phenylacetate CoA-ligase, putative
Gmet_1818	GSU1729	phenylacetate CoA-ligase, putative
Gmet_1825	GSU1737	phenylacetate CoA-ligase, putative
Gmet_1800	no ortholog	coenzyme F390 synthetase family protein
Gmet_2007	no ortholog	coenzyme F390 synthetase family protein
Gmet_0256	GSU0229	medium-chain fatty acyl CoA-ligase, putative
Gmet_2692	GSU1103	unknown
Gmet_0306	no ortholog	fused to unique domain
Gmet_1469	no ortholog	unknown
Gmet_2020	no ortholog	unknown
Gmet_2229	no ortholog	unknown
Gmet_2246	no ortholog	unknown
Gmet_2252	no ortholog	unknown (frameshifted)
Gmet_2143	no ortholog	benzoate CoA-ligase

Gmet_2340	no ortholog	acetyl-CoA synthetase
**Acyl-CoA dehydrogenases or reductases**		
Gmet_1523	no ortholog	benzylsuccinyl-CoA dehydrogenase
Gmet_1710	no ortholog	unknown
Gmet_1715	no ortholog	unknown
Gmet_2075	no ortholog	glutaryl-CoA dehydrogenase
Gmet_2198	no ortholog	unknown
Gmet_2199	no ortholog	unknown
Gmet_2211	no ortholog	unknown
Gmet_2270	no ortholog	unknown
Gmet_3289	no ortholog	isovaleryl-CoA dehydrogenase
Gmet_3306	no ortholog	unknown
Gmet_3307	no ortholog	unknown
Gmet_2087	no ortholog	benzoyl-CoA reductase (electron transfer proteins Gmet_2080, Gmet_2081, Gmet_2083, Gmet_2084, Gmet_2085, Gmet_2086)
Gmet_2134-Gmet_2135-Gmet_2136	no ortholog	4-hydroxybenzoyl-CoA reductase subunits
**Electron transfer flavoprotein beta and alpha subunits and iron-sulfur cluster-binding proteins**		
Gmet_0609-Gmet_0610	GSU2873-GSU2872	with *N*-acetylglutamyl-5-phosphate reductase Gmet_0608? The unpaired beta subunit has four frameshifts in *G. metallireducens*
Gmet_0683-Gmet_0684-Gmet_0685	GSU2797-GSU2796-GSU2795	unknown; related to Gmet_2067-Gmet_2066-Gmet_2065 and Gmet_2258-Gmet_2257-Gmet_2255
Gmet_1525-Gmet_1526-Gmet_1527	no ortholog	with benzylsuccinyl-CoA dehydrogenase Gmet_1523?
Gmet_1714-Gmet_1713-Gmet_1712	no ortholog	unknown; related to Gmet_2070, Gmet_2153-Gmet_2152, Gmet_2264-Gmet_2263-Gmet_2262 and Gmet_3300-Gmet_3301
Gmet_2067-Gmet_2066-Gmet_2065	no ortholog	unknown; adjacent to succinyl-CoA synthetase; related to Gmet_0683-Gmet_0684-Gmet_0685 and Gmet_2258-Gmet_2257-Gmet_2255
Gmet_2070	no ortholog	unknown; adjacent to succinyl-CoA synthetase; iron-sulfur cluster-binding protein related to Gmet_1714 and Gmet_2264
Gmet_2153-	no ortholog	with 6-hydroxycyclohex-1-ene-1-carbonyl-

Gmet_2152		CoA dehydrogenase Gmet_2151? Related to Gmet_1713-Gmet_1712, Gmet_2263-Gmet_2262 and Gmet_3300-Gmet_3301
Gmet_2258-Gmet_2257-Gmet_2255	no ortholog	unknown; adjacent to succinyl-CoA synthetase; related to Gmet_0683-Gmet_0684-Gmet_0685 and Gmet_2067-Gmet_2066-Gmet_2065
Gmet_2264-Gmet_2263-Gmet_2262	no ortholog	unknown; adjacent to succinyl-CoA synthetase; related to Gmet_1714-Gmet_1713-Gmet_1712, Gmet_2070, Gmet_2153-Gmet_2152 and Gmet_3300-Gmet_3301
Gmet_2267-Gmet_2266-Gmet_2265	no ortholog	unknown
Gmet_3300-Gmet_3301	no ortholog	unknown; related to Gmet_1713-Gmet_1712, Gmet_2153-Gmet_2152 and Gmet_2263-Gmet_2262
**Enoyl-CoA hydratases/isomerases**		
no ortholog	GSU1377	unknown
Gmet_1524	no ortholog	phenylitaconyl-CoA hydratase
Gmet_1572	no ortholog	unknown
Gmet_1575	no ortholog	unknown
Gmet_1716	no ortholog	unknown; related to Gmet_2071
Gmet_2057	no ortholog	unknown; related to Gmet_3284
Gmet_2071	no ortholog	unknown; related to Gmet_1716
Gmet_2088	no ortholog	6-oxocyclohex-1-ene-1-carbonyl-CoA hydrolase; related to Gmet_3305
Gmet_2150	no ortholog	cyclohexa-1,5-dienecarbonyl-CoA hydratase
Gmet_2195	no ortholog	unknown
Gmet_2196	no ortholog	unknown
Gmet_2204	no ortholog	unknown
Gmet_2207	no ortholog	unknown; related to Gmet_2212
Gmet_2212	no ortholog	unknown; related to Gmet_2207
Gmet_2224	no ortholog	unknown
Gmet_3284	no ortholog	unknown; related to Gmet_2057
Gmet_3291	no ortholog	unknown
Gmet_3305	no ortholog	unknown; related to Gmet_2088
**3-hydroxyacyl-CoA dehydrogenases**		
Gmet_1530-Gmet_1531	no ortholog	2-[hydroxy(phenyl)methyl]-succinyl-CoA dehydrogenase subunits
Gmet_1717	no ortholog	unknown; related to Gmet_2072
Gmet_2072	no ortholog	unknown; related to Gmet_1717
Gmet_2203	no ortholog	unknown

Gmet_2269	no ortholog	unknown
**Acyl-CoA thioesterases**		
no ortholog	GSU0196	unknown
Gmet_0769	GSU0796	unknown
Gmet_1672	GSU0993	unknown
Gmet_1692	GSU0239	unknown
Gmet_1721	no ortholog	unknown
Gmet_2063	no ortholog	unknown
Gmet_2228	no ortholog	unknown
Gmet_2234	no ortholog	unknown
Gmet_2238	no ortholog	unknown
Gmet_2253	no ortholog	unknown
Gmet_2669	GSU1128	unknown
Gmet_3556	GSU3461	unknown
**Acyl-CoA thiolases**		
Gmet_0144	GSU3313	unknown
Gmet_1528-Gmet_1529	no ortholog	benzoylsuccinyl-CoA thiolase subunits
Gmet_1719	no ortholog	unknown; related to Gmet_2074, Gmet_3302
Gmet_2058	no ortholog	unknown
Gmet_2074	no ortholog	unknown; related to Gmet_1719, Gmet_3302
Gmet_2197	no ortholog	unknown
Gmet_2213	no ortholog	unknown
Gmet_2268	no ortholog	unknown
Gmet_3302	no ortholog	unknown; related to Gmet_1719, Gmet_2074
