# Supplementary material for: The genome sequence of Geobacter metallireducens: features of metabolism, physiology and regulation common and dissimilar to Geobacter sulfurreducens
Source: BMC Microbiol. 2009 May 27;9:109. doi: 10.1186/1471-2180-9-109 (PMC2700814; doi:10.1186/1471-2180-9-109)
Supplement: Additional File 3 — Table S3. Predicted binding sites of the global regulator ModE in the genome of G. sulfurreducens, which are mostly absent from the G. metallireducens genome. This table lists the predicted ModE-binding sites of G. sulfurreducens and compares them to the corresponding sequences in G. metallireducens. [file 1471-2180-9-109-S3.pdf]

Table S3. Predicted binding sites of the global regulator ModE in the genome of *G. sulfurreducens*, which are mostly absent from the *G. metallireducens* genome.

<table>
<tr><th>ModE-binding site in G. sulfurreducens; alignment with G. metallireducens</th><th>Genes predicted to be controlled</th><th>Parallel in G. metallireducens</th></tr>
<tr><td>TATTATAAAGGTAATAAGACATTGACA</td><td>5’ of GSU0759.2, GSU0759.1, GSU0759 (hypothetical proteins)</td><td>Homologous genes absent</td></tr>
<tr><td>AGGTATCCAATCATGAATACTTCGCCA
|| || | || | || || |||||||
AGATAACGAACCGGGAGTATTTCGCCA</td><td>Within GSU3378 (glutamate-ammonia ligase adenylyltransferase), 5’ of GSU3377 (endonuclease III-related)</td><td>Site not detected by ScanAce within Gmet_0071 (GSU3378 homolog), but 70% conserved</td></tr>
<tr><td>TTTTAAAATAAACATAATTTTATAACA
|| ||||||| || ||||||||||||
TTGTAAAATATACTCAATTTTATAACA</td><td>5’ of GSU2490 (membrane protein), GSU2491 (major facilitator family transporter), GSU2492 (sensor histidine kinase)</td><td>Gmet_2465 (GSU2490 homolog) present, homologs of other genes absent; site detected by ScanAce, 85% conserved</td></tr>
<tr><td>CTGCGCTAAATACATACCTCTCTATCG
| |||||| | | ||| |||
CATGGGCTAAAAAGTTGTACTCAATCA</td><td>5’ of GSUR045 (tRNA-Gly)</td><td>Site not detected by ScanAce 5’ of Gmet_R0040 (tRNA-Gly), 56% conserved</td></tr>
<tr><td>AAACGCTATAACCTTCATGACATAACG
|| || | | || |
AATGACTTTTGGTATGCTGCGCAATGC</td><td>5’ of GSU2963, GSU2962, GSU2961, GSU2960 (modDABC); 3’ of GSU2964 (modE)</td><td>modD absent, modABC present; site not detected by ScanAce 3’ of truncated modE homolog (Gmet_0511)</td></tr>
<tr><td>TTTAAAAATATTAATTTATTTATAAAC</td><td>5’ of GSU3415, GSU3416, GSU3417 (oxidoreductase, conserved hypothetical protein, dioxygenase)</td><td>Genes present as Gmet_1705, Gmet_1704, Gmet_2446, but 5’ region is not syntenous</td></tr>
<tr><td>CAGAGATGGATTAATGAGTTTATATTG</td><td>Within GSU1363 (RNA-directed DNA polymerase), 5’ of GSU1364-GSU1365 (restriction system)</td><td>Homologous genes absent</td></tr>
<tr><td>CATTGAAAGATTAATGACTGCATGGCC</td><td>5’ of GSU1715 (hypothetical protein)</td><td>Homologous gene absent</td></tr>
<tr><td>TTTCACCATGAACTATCCTTCATATCG</td><td>5’ of GSU1383 (exonuclease, possibly CRISPR2-associated)</td><td>Homologous gene absent</td></tr>
<tr><td>CTTCAAAATGTTCAAAAATATCAAACC</td><td>5’ of GSU3407, GSU3408 (membrane</td><td>Homologous genes absent</td></tr>
</table>

<table>
<tr><td></td><td>protein, threonine aldolase), possibly also GSU3406, GSU3405, GSU3404 (amino acid transporter)</td><td></td></tr>
<tr><td>CGTTACACAAATACGCATATAGTGATA
| ||| | || ||| ||
CCTTAACAACCCCGTCAAGGGGTGGTA</td><td>5’ of GSU2429 (peptidyl-prolyl cis-trans isomerase)</td><td>Site not detected by ScanAce 5’ of Gmet_0815 (GSU2429 homolog), 44% conserved</td></tr>
<tr><td>TGTCAATGTCAAAATGTATTGCTAATA</td><td>5’ of GSU2150.1 (conserved hypothetical protein)</td><td>Homologous gene absent</td></tr>
<tr><td>CGTCGCTGTAAACAACAAAACAGAACG
  | ||||| || || ||| ||||
TTTGCTTGTAATCACCATCACAAAACG</td><td>5’ of GSU0490 (succinyl:acetate CoA-transferase)</td><td>Site not detected by ScanAce 5’ of Gmet_3044 (GSU0490 homolog), 63% conserved</td></tr>
<tr><td>ATTGGTTATATTCCAGGCTTTATAACC</td><td>Within GSU1945 (fibronectin type III domain protein)</td><td>Homologous gene absent</td></tr>
<tr><td>AAGCCTGGTATTGATATCTCCATAACC
 | | || || |||||| |||| |||
GAAACGGGGATCGATATCGCCATTACC</td><td>Within GSU0660 (4-diphosphocytidyl-2C-methyl-D-erythritol kinase)</td><td>Site not detected by ScanAce within Gmet_2849 (GSU0660 homolog), 70% conserved</td></tr>
<tr><td>AGGCGCAGTAAACATTATTGAATGTCA</td><td>5’ of GSU3406, GSU3405, GSU3404 (amino acid transporter), possibly also GSU3407, GSU3408 (membrane protein, threonine aldolase)</td><td>Homologous genes absent</td></tr>
<tr><td>ATACAAAATAATATTAAAGTGATAGTG</td><td>Within GSU2395 (conserved hypothetical protein)</td><td>Homologous gene absent</td></tr>
<tr><td>ATATATATACAAAATTATATATTGCGT
||||||||| | ||||| || |||
ATATATATAAATTCATATATTTTACGT</td><td>5’ of GSU0391, GSU0392, GSU0393, GSU0394, GSU0395 (efflux pump), maybe more</td><td>Genes present (except GSU0393) as Gmet_3129, Gmet_3128, Gmet_3127, Gmet_3126, maybe more; site not detected by ScanAce, 74% conserved</td></tr>
</table>
