# Supplementary material for: The genome sequence of Geobacter metallireducens: features of metabolism, physiology and regulation common and dissimilar to Geobacter sulfurreducens
Source: BMC Microbiol. 2009 May 27;9:109. doi: 10.1186/1471-2180-9-109 (PMC2700814; doi:10.1186/1471-2180-9-109)
Supplement: Additional File 4 — Figure S1. A family of 24 predicted short RNA elements in the G. metallireducens genome. This is an alignment of 24 DNA sequences that were matched by nucleotide-level BLAST. Each RNA is found in an intergenic region, e.g. the 5' regions of genes affecting lysine/arginine metabolism, and contains a central palindromic structure GRCGTAGCGCTGCTACGCC. Similar sequences were found in the genomes of G. sulfurreducens, G. uraniireducens, and Desulfotalea psychrophila. The sequence strand and start and stop nucleotide positions are indicated. [file 1471-2180-9-109-S4.pdf]

Gmet_R1001	(+)	23218	23332	GTGCTCTTTACGCCATCTTGG-CGC-CGTCCTC-GTCACCCCGTTGTGCGGCGTAGC-TGGGCTACGCCTCCGCCGGGGT-TCCTGCGGG-TGCACCAATC-TGACGTAAATATCACGTTCC
Gmet_R1002	(+)	38738	38849	GCGGCTTTTCCGCAATCTCTG-CGT-TGATCTTCATAATTGC-TTGTGCGGCGTAGC-GCTGCTACGCCTCCGCGCAATC--ATTCGATC--GCCTTGACCTTG--CGAACAATCCACGTTT
Gmet_R1003	(+)	112100	112213	GCCGTATTCAGGCCATCTTTGGCCG--GTTCTCAATCGCACA-ATCCTCGACGTAGC-GCTGCTACGCCTGCGGTTGTACTCATTCGGCCCGACCA-AATC-TGA-CCTA-AATCCATCCGC
Gmet_R1004	(+)	183346	183457	GTGGCTTTTCCGCAATCTCTG-CGTA-AGTCTTCGGGACCGC-TTGTGCGACGTAGC-GCTGCTACGTCTCCGCGCAGTC--CTTCGACA--GCCTTGATCTTG--CGAAAAATCCACGTTT
Gmet_R1005	(+)	246545	246655	GCCGCTTTTTGCCAATCTCGG-CGT--TTTCTGCGTCATCGC-GTGTGCGACGTAGC-GCTGCTACGTCTCCGTGCTCTT--CCTTGAC---GCCTTGACCTTGG-CGAAAAATCGACATCC
Gmet_R1006	(+)	281955	282068	GCGGCTTTTACGCCATCTCGC-CGC-CGTCCTC-GTCGCTCCCTTGTGCGGCGTAGC-GCTGCTACGCCTCCGCGGTCTC-TCCTGCGGG-TGCGACGATC-TGG-TGCAAAATCCGCAGCC
Gmet_R1007	(+)	589201	589314	GCGGCTTTCACACCGGCTCGC-CGC-CGTCCTC-GTCGCTCCTTTGTGCGGCGTAGC-GCTGCTACGCCTCCGCAGTCTC-TCCTGCGGG-TGCGACGATC-CGG-CGCAAAATCCGCAGCC
Gmet_R1008	(+)	725630	725742	AACGCTTTTGGGCCATCTCGG-CGT--TTCCTGCGTCATCGC-TTGTGCGACGTGGC-GCGGCCACGCCTCCGCGCTCTT--CCTTGGAAGC-CGCCGACC-TGACCTCGAAATCGTTCGCC
Gmet_R1009	(-)	972328	972440	GCCGCTTTTCCGCCATCTCGG-CGT-TGTCGTTCGGAATCACCTTGTGCGGCGTAGC-CCTGCTACGCCTCCGCGGGATC--CCTCCTCC--GCCTTGACCTTG--CGAAAAATCGCCATTT
Gmet_R1010	(-)	992050	992163	CGCCATATTCGCGCGCTAACGACGT--CTGCTC-GTCGAGAGGCTCCTCGACGTAGC-GCTGCTACGCCTGCGGGCCTCTCTCCTCTCATCCGCCGTTATCATCA-CGA---ATCTGACGCC
Gmet_R1011	(+)	993381	993494	GCAATCTTCCGACCATCTCCC-CGC-CGTCCTCGGAATGCTCGTTGTGCGGCGTAGC-GCTGCTACGCCTCCGCCGGCAT-CCCTGCGGG-TGCGAGGATC-TGGCCGA--AATCTTGCGCT
Gmet_R1012	(+)	994727	994840	GATATCTTCCGACCATCTCGA-CGC-CGTCCTCGTAAGCCTCGTTGTGCGGCGTAGC-GCTGCTATGCCTCCGCCGGGCT-TCCTGCGGG-TGCGCCGATC-TGGCCGA--AATCTCTCACC
Gmet_R1013	(-)	994880	994993	CGCCATATTTGCGCCCTTCCGCCGT--CGGCTC-GTCGTCAGGCTCCTCTACGTAGC-GCTGCTACGCCTGCGGGCCTTCCTCCTCTCCGCCAACGGAATCATCG-CAA---ATCTGACGCC
Gmet_R1014	(-)	1161076	1161188	GCCGCTTTCGCGCAATCCCGG-CGT-TGCCCTTCGCGATGCTCTTGTGCGGCGTAGC-GCTGCTACGCCTCCGCGGCATC--ACTCGGAC--GCCTTGGCCTTGCACGA--ATTCGACACCC
Gmet_R1015	(+)	1169395	1169509	TCCGCCTTCGGGGCATCTTCGACCC--GGCCTCAATCCCATA-ATCCTCGACGTAGT-GCTGCTACGCCTGCGGTTATGCTCAATCGGCCAGGCCA-AATC-CGCCCCA-AATTCGAACGCG
Gmet_R1016	(-)	1259053	1259167	GCGGCTTTCACGCCATCTCGC-CGC-CGTCCTC-GTCGTTTCCTTGTGCGGCGTAGCTTGGGCTACGCCTCCGCGGTCGC-TCCTGCGGG-TGCGACGATC-TG-CCGCAAATTCCGCAGCC
Gmet_R1017	(-)	1925872	1925983	GCGGCTTTTCCGCAATCTCTG-CGT-TGATCTTCGGGATTGC-TTGTGCGACGTAGC-GCTGCTACGCCTCCGCGCAATC--CCTCGATC--GCCTTGACCTTG--CGAAAAATCCACGTTC
Gmet_R1018	(-)	1936132	1936245	GCGGTCTTCCGACCATTTTGC-CGTCCGTCCTCGGTCCGTTC-TTGTGCGGCGTAGC-GCTGCTACGCCTCCGCGACGGC--CCTGTGGGGGACGACAATC-TGGCCGA--AATCTCACGCC
Gmet_R1019	(-)	2127519	2127632	GCGGATTTCACACCATCTCGC-CGC-CGTCCTC-GTCGCTCCTTTGTGCGGCGTAGC-GCTGCTACGCCTCCGCAGTCCC-TCCTGCGGG-TGCGACGATC-TGG-CACAAACTCCACAGCC
Gmet_R1020	(-)	2557506	2557618	GCCGCTTTCGCGCAATCTCGG-CGTTGCCCTGCGGTCTGCTC-TTGTGCGGCGTAGC-GCTGCTACGCCTCCGCGGCATT--CCTTGGGC--GCCTTGACCTTGCACGA--AATCGACAGCC
Gmet_R1021	(-)	3494616	3494731	TCCGTCTGCACGCCATCTTCGACCG--CTCCTCAATCGCGCA-ATCCTCGACGTAGCTCTGGCTACGCCTGCGGTTGCGCTCAATCGTCGCGGCCA-AATC-TGG-CGCACATACGAACGCG
Gmet_R1022	(-)	3506092	3506204	GCCACTTTTCCGCAATATCTG-CGTA-AGTCTTCGGTATCGC-TTGTGCGGCGTACCAACAGGTACGCCTCCGCGCAATT--CCTCGACA--GCCTTGATCTTG--CGAAAAATTGACGTTC
Gmet_R1023	(+)	3543874	3543987	GCGGCTTTTGCGCCCTGGCGT-TGT-TGCCCTGCGCGAGCAC-TTGTGCGGCGTACCAAGGGGTACGCCTCCGCGTGCTT--GCTCGGGC--GCCTAGCCAGGA--CACAAAATCCACGTTC
Gmet_R1024	(+)	3703651	3703763	GCGGCTTTTCCGCAATCTCTG-CGT-TTATCTTCGGAATTTGCTTGTGCGACGTAGC-GCTGCTACGCCTCCGCGCAATT--CCTAGATC--GCCTTGACCTTG--CGAAAAATCCACGTTC
