# Supplementary material for: The genome sequence of Geobacter metallireducens: features of metabolism, physiology and regulation common and dissimilar to Geobacter sulfurreducens
Source: BMC Microbiol. 2009 May 27;9:109. doi: 10.1186/1471-2180-9-109 (PMC2700814; doi:10.1186/1471-2180-9-109)
Supplement: Additional File 5 — Table S4. Genes found next to multicopy nucleotide sequences of unknown function in G. metallireducens. This table lists the genes adjacent to all of the multicopy nucleotide sequences identified in the G. metallireducens genome. [file 1471-2180-9-109-S5.pdf]

Table S4. Genes found next to multicopy nucleotide sequences of unknown function in *G. metallireducens*.

Locus tag	Nearby Genes	Annotations of nearby gene(s)
**49 RNA elements containing four GGGACCG repeats (Figure S2)**		
Gmet_H401	within Gmet_0369	20 kDa subunit of NADH ubiquinone oxidoreductase or formate dehydrogenase
Gmet_H402	3' of Gmet_0382<br>5' of Gmet_0383	UDP-N-acetylglucosamine 1-carboxyvinyltransferase<br>ATP phosphoribosyltransferase, short form
Gmet_H403	within Gmet_0415 (linker between fused proteins)	fusion protein: D-alanine--D-alanine ligase and cell division protein FtsQ
Gmet_H404	3' of Gmet_0433<br>5' of Gmet_0434	*flgH*; flagellar L-ring protein FlgH<br>*flgI*; flagellar P-ring protein FlgI
Gmet_H405<br>Gmet_H406	3' of Gmet_0434<br>5' of Gmet_0435	*flgI*; flagellar P-ring protein FlgI<br>flagellar protein FlgJ-like protein
Gmet_H407	3' of Gmet_0467<br>5' of Gmet_0468	*cobT*; nicotinate-nucleotide--dimethylbenzimidazole phosphoribosyltransferase<br>*cobS*; cobalamin 5'-phosphate synthase
Gmet_H408	within Gmet_0468	*cobS*; cobalamin 5'-phosphate synthase
Gmet_H409<br>Gmet_H410	within Gmet_0478	*cbiD*; precorrin-5 C1-methyltransferase
Gmet_H411<br>Gmet_H412<br>Gmet_H413	within Gmet_0485	*cobQ*; precorrin-3B C17-methyltransferase/adenosylcobyric acid synthase
Gmet_H414<br>Gmet_H415	3' of Gmet_0544<br>5' of Gmet_0543	(R)-2-hydroxyglutaryl-CoA dehydratase activator, putative<br>cytochrome *c*, 2 heme-binding sites
Gmet_H416	3' of Gmet_0698<br>5' of Gmet_0699	*metC-1*; cystathionine beta-lyase<br>*metC-2*; cystathionine beta-lyase
Gmet_H417	within Gmet_0714<br>5' of Gmet_0715	NUDIX hydrolase<br>membrane protein, putative
Gmet_H418	3' of Gmet_0721<br>5' of Gmet_0722	conserved hypothetical protein<br>SEC-C motif domain protein
Gmet_H419<br>Gmet_H420	3' of Gmet_0905<br>5' of Gmet_0906	ATPase, AAA family<br>conserved hypothetical protein
Gmet_H421	3' of Gmet_1753<br>5' of Gmet_1752	glutamine amidotransferase, class-II<br>archaeal-type glutamate synthase subunit, putative
Gmet_H422	3' of Gmet_2328<br>5' of Gmet_2327	YicC family stress-induced protein TIGR00255<br>*gmk*; guanylate kinase
Gmet_H423	3' of Gmet_2349<br>5' of Gmet_2348	lipid A export ATP-binding/membrane protein, MsbA family<br>*kdtA*; 3-deoxy-D-manno-octulosonic-acid transferase
Gmet_H424	within Gmet_2545	ABC transporter, ATP-binding protein

Gmet_H425<br>Gmet_H426	3’ of Gmet_2562<br>5’ of Gmet_2563	sensor histidine kinase (PAS, HATPase_c)<br>sigma-54-dependent transcriptional response regulator (REC, sigma-54 interaction, HTH8)
Gmet_H427<br>Gmet_H428	3’ of Gmet_2563<br>5’ of Gmet_2564	sigma-54-dependent transcriptional response regulator (REC, sigma-54 interaction, HTH8)<br>YcaC-related hydrolase, putative
Gmet_H429	within Gmet_2764	*lpdA-1*; 2-oxoglutarate dehydrogenase complex, E3 protein, dihydrolipoamide dehydrogenase
Gmet_H430<br>Gmet_H431<br>Gmet_H432	within Gmet_2766	*sucB*; 2-oxoglutarate dehydrogenase, E2 protein, dihydrolipoamide succinyltransferase
Gmet_H433	3’ of Gmet_2776<br>3’ of Gmet_2777	UDP-2,3-diacylglucosamine hydrolase, putative<br>conserved hypothetical protein
Gmet_H434<br>Gmet_H435	5’ of Gmet_2819<br>5’ of Gmet_2820	putative rRNA methylase<br>*shc-1*; squalene cyclase domain protein
Gmet_H436	3’ of Gmet_2891<br>within Gmet_2890	conserved hypothetical protein<br>glycosyl transferase, group 1
Gmet_H437	within Gmet_2892	glycosyl transferase, family 2
Gmet_H438	3’ of Gmet_2898<br>5’ of Gmet_2897	cytochrome *c*, 8 heme-binding sites<br>conserved hypothetical protein
Gmet_H439	within Gmet_2906	conserved hypothetical protein
Gmet_H440	3’ of Gmet_2909<br>5’ of Gmet_2910	*thiE*/*thiD*; 4-amino-5-hydroxymethyl-2-methyl-pyrimidine phosphate kinase and thiamin monophosphate synthase<br>*thiC-1*; 4-amino-5-hydroxymethyl-2-methylpyrimidine synthetase
Gmet_H441	3’ of Gmet_2986<br>3’ of Gmet_2987	heat shock protein, Hsp20 family<br>ATP alpha-hydrolase TIGR00268
Gmet_H442	3’ of Gmet_3096<br>5’ of Gmet_3095	*fliQ*; flagellar biogenesis protein FliQ<br>*fliR*; flagellar biogenesis protein FliR
Gmet_H443	3’ of Gmet_3097<br>5’ of Gmet_3096	*fliP*; flagellar biogenesis protein FliP<br>*fliQ*; flagellar biogenesis protein FliQ
Gmet_H444	3’ of Gmet_3157<br>5’ of Gmet_3158	sensor histidine kinase (HAMP, HisKA, HATPase_c)<br>sigma-54-dependent transcriptional response regulator (REC, sigma-54 interaction, HTH8)
Gmet_H445	3’ of Gmet_3158<br>5’ of Gmet_3159	sigma-54-dependent transcriptional response regulator (REC, sigma-54 interaction, HTH8)<br>alpha-glucan phosphorylase
Gmet_H446	3’ of Gmet_3392<br>5’ of Gmet_3391	3-polyprenyl-4-hydroxybenzoate decarboxylase, putative<br>thiamin biosynthesis enzyme ThiH-related radical SAM domain protein
Gmet_H447<br>Gmet_H448	3’ of Gmet_3430<br>5’ of Gmet_3431	heterodisulfide reductase, iron-sulfur binding subunit, putative

		heterodisulfide reductase, cytochrome reductase subunit
Gmet_H449	3' of Gmet_3549<br>3' of Gmet_3550	PPIC-type PPIASE domain protein, SurA family<br>DnaJ-related molecular chaperone
**24 pairs of predicted global regulator binding sites, class 1 (Figure S3)**		
Gmet_I101A<br>Gmet_I101B	3' of Gmet_0006<br>3' of Gmet_0007	conserved hypothetical protein<br>transposase of IS*Gme9*, IS481 family
Gmet_I102A<br>Gmet_I102B	5' of Gmet_0045<br>3' of Gmet_0046	transposase of IS*Gme8*, IS110 family<br>transposase of IS*Gme8*, IS110 family
Gmet_I103A<br>Gmet_I103B	5' of Gmet_0093<br>5' of Gmet_0094	transposase of IS*Gme9*, IS481 family<br>transposase of IS*Gme8*, IS110 family
Gmet_I104A<br>Gmet_I104B	3' of Gmet_0131<br>5' of Gmet_0132	conserved hypothetical protein<br>transposase of IS*Gme8*, IS110 family
Gmet_I105A<br>Gmet_I105B	3' of Gmet_0179<br>5' of Gmet_0180	DTW domain protein<br>putative dehydratase, NodN/MaoC domain
Gmet_I106A<br>Gmet_I106B	5' of Gmet_0488<br>3' of Gmet_0489	transposase of IS*Gme8*, IS110 family<br>conserved hypothetical protein
Gmet_I107A<br>Gmet_I107B	3' of Gmet_0663<br>5' of Gmet_0664	nitrogenase molybdenum-iron protein, alpha chain<br>nitrogenase molybdenum-iron protein, beta subunit
Gmet_I108A<br>Gmet_I108B	3' of Gmet_0669<br>5' of Gmet_0670	nitrogenase molybdenum-iron cofactor biosynthesis protein NifEN<br>transposase of IS*Gme8*, IS110 family
Gmet_I109A<br>Gmet_I109B	3' of Gmet_0722<br>5' of Gmet_0723	SEC-C motif domain protein<br>transposase of IS*Gme8*, IS110 family
Gmet_I110A<br>Gmet_I110B	3' of Gmet_0811<br>3' of Gmet_0812	efflux pump outer membrane protein<br>sigma-54-dependent transcriptional response regulator (REC, sigma-54 interaction)
Gmet_I111A<br>Gmet_I111B	3' of Gmet_0891<br>5' of Gmet_0892	DNA topoisomerase I<br>conserved hypothetical protein
Gmet_I112A<br>Gmet_I112B	5' of Gmet_0958<br>5' of Gmet_0959	transposase of IS*Gme8*, IS110 family<br>type IV prepilin-like proteins leader peptide processing enzyme
Gmet_I113A<br>Gmet_I113B	3' of Gmet_1142<br>5' of Gmet_1143	conserved hypothetical protein<br>transposase of IS*Gme8*, IS110 family
Gmet_I114A<br>Gmet_I114B	5' of Gmet_1717<br>5' of Gmet_1718	3-hydroxyacyl-CoA dehydrogenase<br>transposase of IS*Gme8*, IS110 family
Gmet_I115A<br>Gmet_I115B	5' of Gmet_2067<br>3' of Gmet_2068	electron transfer flavoprotein, beta subunit<br>succinyl-CoA synthetase, alpha subunit
Gmet_I116A<br>Gmet_I116B	5' of Gmet_2072<br>5' of Gmet_2073	3-hydroxyacyl-CoA dehydrogenase<br>transposase of IS*Gme8*, IS110 family
Gmet_I117A<br>Gmet_I117B	5' of Gmet_2493<br>3' of Gmet_2494	tryptophan synthase, alternate beta subunit, putative<br>indole-3-glycerol phosphate synthase
Gmet_I118A<br>Gmet_I118B	5' of Gmet_2517<br>3' of Gmet_2518	efflux pump outer membrane protein<br>efflux pump inner membrane protein

Gmet_I119A<br>Gmet_I119B	3’ of Gmet_2784<br>3’ of Gmet_2785	cobyrinic acid a,c-diamide synthase family protein<br>conserved hypothetical protein
Gmet_I120A<br>Gmet_I120B	3’ of Gmet_2809<br>5’ of Gmet_2810	protein of unknown function DUF490<br>transposase of IS*Gme8*, IS110 family
Gmet_I121A<br>Gmet_I121B	5’ of Gmet_2975<br>3’ of Gmet_2976	transposase of IS*Gme8*, IS110 family<br>peptidyl-tRNA hydrolase-related protein
Gmet_I122A<br>Gmet_I122B	3’ of Gmet_3244<br>5’ of Gmet_3245	conserved hypothetical protein<br>FAD-dependent dehydrogenase with ferredoxin-like domain
Gmet_I123A<br>Gmet_I123B	5’ of Gmet_3328<br>3’ of Gmet_3329	glyoxalase/bleomycin resistance protein/dioxygenase superfamily protein<br>periplasmically oriented, membrane bound [NiFe]-hydrogenase maturation protease
Gmet_I124A<br>Gmet_I124B	3’ of Gmet_3428<br>5’ of Gmet_3429	heterodisulfide reductase subunit<br>transposase of IS*Gme8*, IS110 family
**21 pairs of predicted global regulator binding sites, class 2 (Figure S4)**		
Gmet_I201A<br>Gmet_I201B	3’ of Gmet_0052<br>3’ of Gmet_0053	peptidase, M16 family<br>transglutaminase domain protein
Gmet_I202A<br>Gmet_I202B	5’ of Gmet_0188<br>3’ of Gmet_0189	metal-dependent hydrolase, beta-lactamase superfamily III<br>delta-aminolevulinic acid dehydratase
Gmet_I203A<br>Gmet_I203B	3’ of Gmet_0471<br>5’ of Gmet_0472	4-amino-5-hydroxymethyl-2-methylpyrimidine synthetase<br>cobalt ABC transporter membrane protein CbiM, N-terminal domain
Gmet_I204A<br>Gmet_I204B	3’ of Gmet_0564<br>5’ of Gmet_0565	conserved hypothetical protein<br>conserved hypothetical protein
Gmet_I205A<br>Gmet_I205B	3’ of Gmet_0568<br>5’ of Gmet_0569	ABC transporter, periplasmic protein<br>conserved hypothetical protein
Gmet_I206A<br>Gmet_I206B	3’ of Gmet_0696<br>5’ of Gmet_0697	sensor histidine kinase (HisKA, HATPase_c)<br>inositol-1-monophosphatase
Gmet_I207A<br>Gmet_I207B	5’ of Gmet_0776<br>3’ of Gmet_0777	sigma-54-dependent nitrogen response regulator NtrX (REC, sigma-54 interaction, HTH8)<br>nitrogen regulation sensor histidine kinase NtrY (HAMP, PAS, HisKA, HATPase_c)
Gmet_I208A<br>Gmet_I208B	3’ of Gmet_0990<br>5’ of Gmet_0991	phosphoglucomutase/phosphomannomutase family protein<br>nitrogen fixation iron-sulfur cluster assembly protein NifU
Gmet_I209A<br>Gmet_I209B	3’ of Gmet_2509<br>5’ of Gmet_2510	pyruvate dehydrogenase complex E1 protein, alpha subunit<br>pyruvate dehydrogenase complex E1 protein, beta subunit
Gmet_I210A<br>Gmet_I210B	3’ of Gmet_2572<br>3’ of Gmet_2573	DUF343-containing conserved hypothetical protein<br>ATP-dependent RNA helicase RhlE

Gmet_I211A<br>Gmet_I211B	5' of Gmet_2674<br>3' of Gmet_2675	predicted zinc-dependent hydrolase<br>metal-dependent phosphohydrolase, HD superfamily
Gmet_I212A<br>Gmet_I212B	3' of Gmet_2723<br>3' of Gmet_2724	peroxiredoxin<br>ATP-dependent helicase, UvrD superfamily
Gmet_I213A<br>Gmet_I213B	3' of Gmet_2737<br>5' of Gmet_2738	sensor histidine kinase (HAMP, HisKA, HATPase_c)<br>molybdopterin-binding iron-sulfur oxidoreductase MopB-3
Gmet_I214A<br>Gmet_I214B	3' of Gmet_2979<br>3' of Gmet_2980	hypothetical protein<br>ErfK/YbiS/YcfS/YnhG family protein
Gmet_I215A<br>Gmet_I215B	3' of Gmet_2993<br>5' of Gmet_2994	histidine triad (HIT) family hydrolase<br>endonuclease IV
Gmet_I216A<br>Gmet_I216B	3' of Gmet_3236<br>5' of Gmet_3237	magnesium-dependent DNase TatD<br>YgdL family protein
Gmet_I217A<br>Gmet_I217B	3' of Gmet_3245<br>5' of Gmet_3246	FAD-dependent dehydrogenase with ferredoxin-like domain<br>iron-sulfur oxidoreductase, putative
Gmet_I218A<br>Gmet_I218B	3' of Gmet_3248<br>5' of Gmet_3249	biotin-dependent acyl-CoA carboxyltransferase<br>conserved hypothetical protein
Gmet_I219A<br>Gmet_I219B	3' of Gmet_3252<br>5' of Gmet_3253	conserved hypothetical protein<br>glyoxalase/bleomycin resistance protein/dioxygenase family protein
Gmet_I220A	5' of Gmet_3273<br>5' of Gmet_3274	conserved hypothetical protein<br>conserved hypothetical protein
Gmet_I221A<br>Gmet_I221B	3' of Gmet_3402<br>3' of Gmet_3403	thiamine biosynthesis protein ThiI-related adenine nucleotide alpha hydrolase superfamily protein<br>transglycosylase-related protein
Gmet_I222A<br>Gmet_I222B	3' of Gmet_3425<br>5' of Gmet_3426	heterodisulfide reductase, subunit B<br>heterodisulfide reductase, subunit A
**8 pairs of predicted global regulator binding sites, class 3 (Figure S5)**		
Gmet_I301A<br>Gmet_I301B	3' of Gmet_0301<br>5' of Gmet_0302	*moaA-1*; molybdopterin precursor Z synthesis protein MoaA<br>*mosC-1*; molybdenum cofactor sulfurase C-terminal domain protein
Gmet_I302A<br>Gmet_I302B	3' of Gmet_0484<br>5' of Gmet_0485	*cbiG*; precorrin-3B C17-methyltransferase<br>*cobQ*; precorrin-3B C17-methyltransferase/adenosylcobyric acid synthase
Gmet_I303A<br>Gmet_I303B	3' of Gmet_1103<br>5' of Gmet_1104	*greB*; transcription elongation factor GreB<br>mannose-1-phosphate guanylyltransferase
Gmet_I304A<br>Gmet_I304B	3' of Gmet_2672<br>3' of Gmet_2673	conserved hypothetical protein<br>*coaBC*; phosphopantothenoylcysteine decarboxylase/phosphopantothenate--cysteine ligase
Gmet_I305A<br>Gmet_I305B	5' of Gmet_3074<br>3' of Gmet_3075	conserved hypothetical protein TIGR00294<br>adenosine nucleotide-binding predicted transcriptional regulator ExsB

Gmet_I306A<br>Gmet_I306B	5' of Gmet_3115<br>3' of Gmet_3116	*flgB*; flagellar basal-body rod protein FlgB<br>conserved hypothetical protein
Gmet_I307A<br>Gmet_I307B	3' of Gmet_3198<br>5' of Gmet_3199	*proB*; glutamate 5-kinase<br>*proA*; glutamyl-5-phosphate reductase
Gmet_I308A<br>Gmet_I308B	3' of Gmet_3432<br>3' of Gmet_3433	sensor histidine kinase (HisKA, HATPase_c)<br>aspartate/tyrosine/aromatic aminotransferase
**19 predicted global regulator binding sites, class 4 (Figure S6)**		
Gmet_I401	3' of Gmet_0306<br>5' of Gmet_0307	conserved hypothetical protein (CoA-binding, CoA ligase, unique)<br>conserved hypothetical protein
Gmet_I402<br>Gmet_I403	5' of Gmet_0770<br>3' of Gmet_0771	*ppsA*; phosphoenolpyruvate synthase<br>*wrbA*; tryptophan repressor TrpR-binding flavodoxin
Gmet_I404<br>Gmet_I405	5' of Gmet_2021<br>3' of Gmet_2022	hypothetical protein<br>hypothetical protein
Gmet_I406	5' of Gmet_2160<br>5' of Gmet_2161	transposase, IS*Gme6*<br>UDP-N-acetylmuramyl pentapeptide phosphotransferase/UDP-N- acetylglucosamine-1-phosphate transferase/peptidyl-prolyl *cis-trans* isomerase
Gmet_I407<br>Gmet_I408<br>Gmet_I409	5' of Gmet_2580<br>3' of Gmet_2581	excinuclease ABC family protein<br>mechanosensitive ion channel family protein
Gmet_I410	5' of Gmet_2715<br>3' of Gmet_2716	hypoxanthine/guanine transport membrane protein<br>hypoxanthine/guanine phosphoribosyltransferase, putative
Gmet_I411	3' of Gmet_2721<br>3' of Gmet_2722	GAF domain/GGDEF domain protein<br>peptidyl-prolyl *cis-trans* isomerase, cyclophilin-type
Gmet_I412	5' of Gmet_2734<br>5' of Gmet_2735	radical SAM domain iron-sulfur oxidoreductase, putative<br>ligand-gated TonB-dependent porin
Gmet_I413<br>Gmet_I414	5' of Gmet_3080<br>3' of Gmet_3081	thioredoxin/NifU-like domain protein<br>integrase core domain protein, IS*Gme9*
Gmet_I415	5' of Gmet_R0051<br>5' of Gmet_3391	proline tRNA<br>hypothetical protein
Gmet_I416	5' of Gmet_3416<br>3' of Gmet_3417	ABC transporter, ATP-binding/membrane protein<br>predicted GTPase MglA
Gmet_I417<br>Gmet_I418	5' of Gmet_3499<br>3' of Gmet_3500	OmpA domain protein<br>conserved hypothetical protein
Gmet_I419<br>Gmet_I420	3' of Gmet_3508<br>5' of Gmet_3509	efflux pump membrane fusion protein<br>efflux pump inner membrane protein
**24 predicted regulatory short RNAs, group 1 (Figure S1)**		
Gmet_R1001	5' of Gmet_0016	*hemE*; uroporphyrinogen decarboxylase
Gmet_R1002	5' of Gmet_0031	conserved hypothetical protein
Gmet_R1003	3' of Gmet_0088	sigma-54-dependent transcriptional regulator
	3' of Gmet_0089	ATP-dependent DNA helicase UvrD/REP

Gmet_R1004	5’ of Gmet_0153	NADH dehydrogenase I, B/C/D subunits
Gmet_R1005	5’ of Gmet_0213	L,L-diaminopimelate aminotransferase
Gmet_R1006	5’ of Gmet_0251	cytochrome *c* oxidase, subunit IV
Gmet_R1007	5’ of Gmet_0552	transketolase, C-terminal domain
Gmet_R1008	5’ of Gmet_0663	nitrogenase molybdenum-iron protein, alpha chain
Gmet_R1009	5’ of Gmet_0882	polysaccharide deacetylase-related
Gmet_R1010	5’ of Gmet_0902	saccharopine dehydrogenase
Gmet_R1011	5’ of Gmet_0903	*nspC*; carboxynorspermidine decarboxylase
Gmet_R1012, Gmet_R1013	5’ of Gmet_0904	*speA*; biosynthetic arginine decarboxylase
Gmet_R1014	5’ of Gmet_1045	aldehyde ferredoxin oxidoreductase, tungsten-containing
Gmet_R1015	5’ of Gmet_1054	sensor histidine kinase (FhlA, HisKA, HATPase_c)
Gmet_R1016	5’ of Gmet_1139	phage baseplate outer wedge protein
Gmet_R1017	5’ of Gmet_1713	electron transfer flavoprotein, beta subunit
Gmet_R1018	5’ of Gmet_1721	thioesterase superfamily protein
Gmet_R1019	5’ of Gmet_1905	*cysT*; sulfate ABC transporter, membrane protein CysT
Gmet_R1020	5’ of Gmet_2257	electron transfer flavoprotein, alpha subunit
Gmet_R1021	3’ of Gmet_3093	conserved hypothetical protein
	3’ of Gmet_3094	*flhB*; flagellar biosynthesis protein FlhB
Gmet_R1022	5’ of Gmet_3106	*fliK*; flagellar hook-length control protein FliK
Gmet_R1023	3’ of Gmet_3145	ferritin-like domain protein
	3’ of Gmet_3146	peptidyl-prolyl *cis-trans* isomerase, FKBP-type
Gmet_R1024	5’ of Gmet_3291	enoyl-CoA hydratase/isomerase
**4 predicted short RNAs of unknown function, group 2 (Figure S8a)**		
Gmet_R2001	5’ of Gmet_0082<br>3’ of Gmet_0083	hypothetical protein<br>hypothetical protein
Gmet_R2002	5’ of Gmet_1003	hypothetical protein
Gmet_R2003	5’ of Gmet_2755	hypothetical protein
Gmet_R2004	5’ of Gmet_2967<br>3’ of Gmet_2968	conserved hypothetical protein<br>conserved hypothetical protein
**16 predicted regulatory short RNAs, group 3 (Figure S7)**		
Gmet_R3001	5’ of Gmet_0131	conserved hypothetical protein
Gmet_R3002	5’ of Gmet_0232	conserved hypothetical protein
Gmet_R3003	5’ of Gmet_0241, Gmet_0240	conserved hypothetical protein, helix-turn-helix and Zn ribbon transcriptional regulator
Gmet_R3004	5’ of Gmet_0292	cytochrome *c*, 2 heme-binding sites
Gmet_R3005	5’ of Gmet_0970-Gmet_0975	transcriptional regulator, XRE family<br>five type IV pilus biogenesis proteins (Figure 8)
Gmet_R3006, Gmet_R3007	5’ of Gmet_1087	cytochrome *c*, 1 heme-binding site
Gmet_R3008, Gmet_R3009	5’ of Gmet_1191, Gmet_1190	cytochrome *c*, 1 heme-binding site<br>efflux pump outer membrane protein
Gmet_R3010	5’ of Gmet_1195	serine protease, subtilase family

Gmet_R3011	5' of Gmet_1703	cytochrome *c*, 7 heme-binding sites
Gmet_R3012	5' of Gmet_1763	conserved hypothetical protein related to Gmet_0131
Gmet_R3013	5' of Gmet_2037	*N*-acylhomoserine lactone synthetase-related protein
Gmet_R3014	5' of Gmet_2168	cytochrome *c*, 2 heme-binding sites
Gmet_R3015,<br>Gmet_R3016	5' of Gmet_3485-<br>Gmet_3487	peptidoglycan-binding ErfK/YbiS/YcfS/YnhG family protein<br>lipoprotein, putative<br>ErfK/YbiS/YcfS/YnhG-related protein
**6 predicted short RNAs, group 4 (Figure S8b)**		
Gmet_R4001	3' of Gmet_0173<br>5' of Gmet_0174	putative lipoprotein<br>conserved hypothetical protein
Gmet_R4002	3' of Gmet_0325<br>5' of Gmet_0326	cytochrome *c*, 4 heme-binding sites<br>oxidoreductase, iron-sulfur cluster-binding subunit
Gmet_R4003	3' of Gmet_2082<br>5' of Gmet_2081	*bamF*; predicted benzoyl-CoA reductase electron transfer protein, selenocysteine-containing<br>*bamG*; predicted benzoyl-CoA reductase electron transfer protein, NADH dehydrogenase 24 kDa subunit-like
Gmet_R4004<br>(fragment)	3' of Gmet_2836<br>5' of Gmet_2835	conserved hypothetical protein<br>nitroreductase 3 family protein
Gmet_R4005	3' of Gmet_3388<br>3' of Gmet_3389	conserved hypothetical protein<br>ribonuclease D, putative
Gmet_R4006	3' of Gmet_3420<br>5' of Gmet_3419	*recR*; recombination protein RecR<br>pyruvate:ferredoxin/flavodoxin oxidoreductase
**8 copies of a palindromic nucleotide sequence, group 5 (Figure S8c)**		
Gmet_R5001	within Gmet_0309	succinate dehydrogenase/fumarate reductase iron-sulfur protein
Gmet_R5002	3' of Gmet_0745<br>5' of Gmet_0746	*ruvA*; Holliday junction resolution DNA helicase RuvA<br>*ruvB*; Holliday junction resolution DNA helicase RuvB
Gmet_R5003	3' of Gmet_1067<br>5' of Gmet_1068	CRISPR-associated TM1801 family protein<br>transposase, IS*Gme16*
Gmet_R5004	within Gmet_1068	CRISPR-associated integrase, IS*Gme16*
Gmet_R5005	5' of Gmet_1069<br>5' of Gmet_1070	transposase, IS*Gme2*<br>RecB family exonuclease Cas4
Gmet_R5006	3' of Gmet_1154<br>3' of Gmet_1155	membrane protein<br>membrane protein, TerC family
Gmet_R5007	3' of Gmet_2410<br>5' of Gmet_2409	superoxide dismutase, iron/manganese-containing<br>intracellular protease, PfpI family, putative
Gmet_R5008	3' of Gmet_3127<br>5' of Gmet_3126	efflux pump inner membrane protein<br>conserved hypothetical protein
**4 RNA elements with complementary ends, group 6 (Figure S8d)**		
Gmet_R6001	3' of Gmet_0475<br>5' of Gmet_0476	*cbiO-1*; cobalt ABC transporter, ATP-binding protein

		cbiX; sirohydrochlorin cobaltochelatase
Gmet_R6002	3' of Gmet_0743<br>5' of Gmet_0744	conserved hypothetical protein TIGR01033<br>*ruvC*; crossover junction endodeoxyribonuclease RuvC
Gmet_R6003	3' of Gmet_1717<br>5' of Gmet_1716	3-hydroxyacyl-CoA dehydrogenase<br>enoyl-CoA hydratase/isomerase
Gmet_R6004	3' of Gmet_3426<br>5' of Gmet_3427	*hdrA*; heterodisulfide reductase, subunit A<br>heterodisulfide reductase subunit
**7 predicted short RNAs, group 7 (Figure S8e)**		
Gmet_R7001	3' of Gmet_0870<br>5' of Gmet_0871	*cysE*; serine *O*-acetyltransferase<br>Rrf2 family transcriptional regulator
Gmet_R7002	3' of Gmet_1210<br>5' of Gmet_1211	*mauG*; methylamine utilization cytochrome *c* peroxidase MauG, 2 heme-binding sites<br>*gap-2*; glyceraldehyde-3-phosphate dehydrogenase, type I
Gmet_R7003	3' of Gmet_2110<br>3' of Gmet_2112	protein of unknown function UPF0261<br>predicted phosphohydrolase
Gmet_R7004	3' of Gmet_2154<br>5' of Gmet_2153	multicopper oxidase, type 2<br>*bamO*; electron transfer flavoprotein, beta subunit
Gmet_R7005	3' of Gmet_2166<br>5' of Gmet_R3014	transposase, IS*Gme5*<br>conserved nucleotide sequence
Gmet_R7006	3' of Gmet_2442<br>5' of Gmet_2441	iron/manganese-dependent transcriptional regulator<br>*cbiM-2*; cobalt ABC transporter membrane protein CbiM
Gmet_R7007	5' of Gmet_2718<br>5' of Gmet_2719	protein of unknown function DUF323<br>conserved hypothetical protein
**4 predicted short RNAs, group 8 (Figure S8f)**		
Gmet_R8001<br>Gmet_R8002	3' of Gmet_1317<br>5' of Gmet_1320	protein of unknown function DUF86<br>nucleotidyltransferase-related protein
Gmet_R8003	3' of Gmet_1320<br>5' of Gmet_1321	nucleotidyltransferase-related protein<br>conserved hypothetical protein
Gmet_R8004	3' of Gmet_1322<br>5' of Gmet_1324	periplasmic or secreted lipoprotein<br>conserved hypothetical protein (LbetaH repeats_WxcM)
**4 predicted short RNAs, group 9 (Figure S8g)**		
Gmet_R9001	3' of Gmet_2486<br>5' of Gmet_2485	hypothetical protein<br>conserved hypothetical protein
Gmet_R9002	3' of Gmet_2490<br>5' of Gmet_R0119	conserved hypothetical protein<br>conserved nucleotide sequence
Gmet_R9003	3' of Gmet_2491<br>5' of Gmet_2490	transcriptional regulator, frameshifted<br>conserved hypothetical protein
Gmet_R9004	3' of Gmet_2534<br>3' of Gmet_2535	transcriptional regulator, putative<br>hypothetical protein
**2 predicted short RNAs (Figure S8h)**		
Gmet_R0064	3' of Gmet_0141	conserved hypothetical protein related to cytochrome

	5' of Gmet_0140	*c* assembly proteins<br>conserved hypothetical protein
Gmet_R0066	3' of Gmet_0257<br>3' of Gmet_0258	membrane protein, putative<br>tRNA dihydrouridine synthase-like flavin mononucleotide-binding domain protein
**2 predicted short RNAs (Figure S8i)**		
Gmet_R0068	near Gmet_R0067<br>3' of Gmet_0312	conserved nucleotide sequence<br>hypothetical protein
Gmet_R0094	3' of Gmet_1608<br>5' of Gmet_1609	*hup-2*; histone-like protein<br>LexA-ImpB/MucB/SamB protein pseudogene fusion
**2 predicted short RNAs (Figure S8j)**		
Gmet_R0074	3' of Gmet_0717<br>5' of Gmet_0718	hypothetical protein<br>hypothetical protein
Gmet_R0075	3' of Gmet_0719<br>5' of Gmet_0720	conserved hypothetical protein<br>conserved hypothetical protein
**2 conserved palindromic sequences (Figure S8k)**		
Gmet_R0091	3' of Gmet_1352<br>5' of Gmet_1353	*glnA*; glutamine synthetase, type I<br>*ispG*; 4-hydroxy-3-methylbut-2-en-1-yl diphosphate synthase
Gmet_R0114	3' of Gmet_2372<br>5' of Gmet_2373	*eno*; enolase<br>nicotinate phosphoribosyltransferase
**2 predicted short RNAs (Figure S8l)**		
Gmet_R0097	5' of Gmet_1702<br>near Gmet_R3011	hypothetical protein<br>conserved nucleotide sequence
Gmet_R0099	3' of Gmet_1703<br>3' of Gmet_1704	cytochrome *c*, 7 heme-binding sites<br>conserved hypothetical protein
**3 predicted short RNAs (Figure S8m)**		
Gmet_R0105	3' of Gmet_2004<br>5' of Gmet_2003	glycosyl transferase<br>membrane protein, putative
Gmet_R0106	3' of Gmet_2006<br>5' of Gmet_2005	*sufS*; selenocysteine lyase<br>polysaccharide deacetylase domain protein
Gmet_R0107	3' of Gmet_2008<br>5' of Gmet_2007	glycosyl transferase<br>coenzyme F390 synthetase superfamily protein
**2 predicted short RNAs (Figure S8n)**		
Gmet_R0110	3' of Gmet_2163<br>5' of Gmet_3609	laccase family protein<br>conserved hypothetical protein
Gmet_R0111	3' of Gmet_2170<br>5' of Gmet_2169	methyltransferase, putative<br>glycosyl transferase, putative
**2 predicted short RNAs (Figure S8o)**		
Gmet_R0126	3' of Gmet_2725<br>5' of Gmet_2724	hypothetical protein<br>ATP-dependent helicase, UvrD superfamily
Gmet_R0127	3' of Gmet_2726<br>5' of Gmet_3623	neutral zinc metallopeptidase, putative<br>hypothetical protein
**2 predicted short RNAs (Figure S8p)**		
Gmet_R0146	3' of Gmet_3471	pirin family protein, C-terminal fragment,

	3’ of Gmet_3472	frameshifted<br>response regulator (REC)
Gmet_R0147	5’ of Gmet_3483<br>5’ of Gmet_R0055	type I restriction-modification enzyme S subunit, fragment<br>Asn tRNA
