# Supplementary material for: The genome sequence of Geobacter metallireducens: features of metabolism, physiology and regulation common and dissimilar to Geobacter sulfurreducens
Source: BMC Microbiol. 2009 May 27;9:109. doi: 10.1186/1471-2180-9-109 (PMC2700814; doi:10.1186/1471-2180-9-109)
Supplement: Additional File 6 — Figure S2. A family of 49 predicted regulatory RNA elements in G. metallireducens, containing four heptanucleotide repeats (consensus GGACCGG). This is an alignment of 49 DNA sequences that were matched by nucleotide-level BLAST. These elements are found within genes, sometimes more than once per gene, as well as between genes. The sequence strand and start and stop nucleotide positions are indicated. [file 1471-2180-9-109-S6.pdf]

Gmet_H401	(-)	412977	413021	TAACGGCCGGGACCGGGGACCAGGGACCGGGGACCGGGAAAACCC
Gmet_H402	(-)	427750	427794	AAAACTAAGGGACCGGGGACCAGGGACCGGGGACCAACAATGGCA
Gmet_H403	(-)	460055	460099	TAAAATCGGTGATCGGGGACCAGGGACCAGGGACCGGCAAAAACA
Gmet_H404	(+)	480226	480270	TAACGGCCGGGACTAGGGACCGGGGACCAGGGACCAGGAAAACCA
Gmet_H405	(+)	481398	481442	TAGAGGCCGGAACCTGGGATCGGGGACCAGGGACCAGGAAAGGCA
Gmet_H406	(-)	481439	481483	AACAGACCGGGACCAGGGACCGGGGATCCGGGACCGGTAAATGCC
Gmet_H407	(+)	515413	515457	AAAAGGCCGGGACCAGTAACCGGGGACCAGGGACTGGGAAAACCT
Gmet_H408	(-)	515519	515563	AAAAGGCGGGGACTGGGGACCGGGGACCGGGGACCGGTAAAAAAC
Gmet_H409	(+)	524206	524250	TACATGCCGGGATCGGGGACCGGGGACCGGGGACCGGGAAAACCT
Gmet_H410	(-)	524445	524489	GTTGTACCGGGACCAGGGGCCGGGGACCGGGGACCGGGAACCCAA
Gmet_H411	(+)	530025	530069	AAAAGGCCGGGACCGGGGACCAGGGACCAGGGACCAGGGAAAAAC
Gmet_H412	(-)	530117	530161	CAAAGACCGGGACCGGGGACCAGGGACCGGGGACCGGGAATTCAA
Gmet_H413	(+)	530867	530911	GAAAAACGGGGATCGGGGACCGGGGACCGGGGACCGGGGGAAAAG
Gmet_H414	(+)	581560	581604	GAAAGGCCGGGACCAGGGACCGGGGACCGGGGACCAGGAAAAACA
Gmet_H415	(-)	581664	581708	AAAATTCGGGGACCCGGGACCGGGGACCGGGGACCGGTAAAAACA
Gmet_H416	(+)	766602	766646	TGAAGGCGGGGACCAGGGATCGGGAACCGGGGACCGGTAAAGGCT
Gmet_H417	(+)	787460	787504	CGCGATCGGGGACCGGGGACCGGGGACCAGGGGCCGGGAAAACCC
Gmet_H418	(+)	795833	795877	TAAAATCCGGGACCGGGGATCGGGGACCAGGGACCGGCAAAAAAG
Gmet_H419	(+)	998562	998606	TAAAGACAGGGACCAGGGACCAGGAACCGGGGACCGGCAAAACCC
Gmet_H420	(-)	998630	998674	GAAAAGCCGGGACCGGGGATCGGGGACCAGGGACCAGGAAAGGCG
Gmet_H421	(-)	1968053	1968097	TGACATCCGGGACCAGGGACCGGGGAGCGGGGACCTGTTACCCCC
Gmet_H422	(+)	2635345	2635389	TAACCCCGGGGACCAGGGACCGGGGACCGGGGACCAGTGAAATCC
Gmet_H423	(-)	2655831	2655875	TAGAGGCCGGGACCGGGGACCAGGGACCAGGGACCGGCGGCAGCG
Gmet_H424	(+)	2888448	2888492	CGGAATCCGGGACCGGGGACCGGGGACCGGGGACCGGGGAAATCT
Gmet_H425	(+)	2909777	2909821	TTAATTCAGTAACCAGGGACCAGGAACCGGGGACCAGTGGAATCA
Gmet_H426	(-)	2909837	2909881	AAACCTCGGAAATCCGGGACCGGGGACCGGGGACCGGTAAAACCG
Gmet_H427	(+)	2911326	2911370	TGAAAGCCGGGACCGGGGATCAGGGACCGGGGATCAGGAAAAATC
Gmet_H428	(-)	2911421	2911465	CGGAATTCGGGACCGGGGACCGGGGACCAGGGACCGGGAAAACCC
Gmet_H429	(-)	3124287	3124331	TGAAGGCGGGGACCAGGGACCGGGGACCGGGGACCGGGAAAAGCA
Gmet_H430	(+)	3126262	3126306	CTGCCGCGGGGACCGGGGACCGGGGACCGGGGACCGGCTCAAAAG
Gmet_H431	(-)	3126379	3126423	TGACAGCCGGGACGGGGGACCGGGGACCGGGGACCGGGAAAGGCG
Gmet_H432	(-)	3126559	3126603	TAGAAGCCGGGACCGGGGACCGGGGACCGGGGACCGGGAAAGGCA
Gmet_H433	(+)	3141389	3141433	GCGATACCGGTACCGGGGACCGGGGACCGGGGACCAGTAAAAACC
Gmet_H434	(+)	3188778	3188822	AAGTCCCCGGGACCGGGGACCAGGGACCGGGGACCGGGCAAAGGC
Gmet_H435	(-)	3188884	3188928	CAAGGGCCGGGACCGGGGACCGGGGACCAGGTTTAGAAGCCAACC
Gmet_H436	(+)	3272024	3272068	GCGTCCTCGGGACCGGGGACCGGGGACCGGGGACCGGGGAAAAAC
Gmet_H437	(-)	3272462	3272506	CAACGTAGGGGACCGGGGACCGGGGACCGGGGACCGGGAAAATCG
Gmet_H438	(-)	3280694	3280738	GTGAGGCCGGGACTGGGGATTGGGGACCAGGGACCGGGAAAGCCA
Gmet_H439	(+)	3289968	3290012	TAAAGACGGGGACCAGGGACCGGGGACCGGGGACCGGGAACGCCA
Gmet_H440	(+)	3294421	3294465	TGAAGTCGGGGACCAGGGACCGGGGACCGGGGACCGGTAATACCG
Gmet_H441	(-)	3373657	3373701	TGAATCTGGGGACCGGGGACCAGGGACCAGGGACCGGGAAAACCG
Gmet_H442	(+)	3496734	3496778	ATAAGTCAGGGACCGGGGACCGGGGACCGGGGACCAGTAACAACT
Gmet_H443	(+)	3497091	3497135	AAACCTCCGGGATCGGGGACCGGGGATCGGGGACCGGGAAAGGCT
Gmet_H444	(+)	3553644	3553688	GAGAAGCCGGGACCGGGGACCAGGGACCGGGGACCAGGAAAAGAC
Gmet_H445	(-)	3555274	3555318	TCCATTGAAACCTCCGGGACCGGGGACCGGGGACCGGGAAAACCC
Gmet_H446	(-)	3806153	3806197	TAAAGGCCGGGACCGGGGATTAGGGACCGGTGACCAGCAACTGAT
Gmet_H447	(+)	3850973	3851017	CCGCGATCGGGACCAGGGACCAGGGACCAGGGACCGGCAAAGACT
Gmet_H448	(-)	3851057	3851101	TAAAATCGGTGATTGGGGACCGGGGACCGGGGACCAGTAAAAATC
Gmet_H449	(+)	3984830	3984874	ATTAGACAGGAAACGGGGACCGGGGACCGGGGACCAGCAAAACCG
