# Supplementary material for: The genome sequence of Geobacter metallireducens: features of metabolism, physiology and regulation common and dissimilar to Geobacter sulfurreducens
Source: BMC Microbiol. 2009 May 27;9:109. doi: 10.1186/1471-2180-9-109 (PMC2700814; doi:10.1186/1471-2180-9-109)
Supplement: Additional File 9 — Figure S5. Predicted global regulator binding sites (class 3). This is an alignment of 16 DNA sequences that were matched by nucleotide-level BLAST. Fifteen of the sites consist of five tandem heptanucleotide repeats (consensus MTYCTGA). Each sequence begins at the right extremity of the top line (the 3' side of the "-" strand of the chromosome), loops on the left side (switching strands), and continues to the right extremity of the bottom line (the 3' side of the "+" strand of the chromosome); start and stop nucleotide positions are indicated. [file 1471-2180-9-109-S9.pdf]

Gmet_I301A	(-)	338111	---------AATGGAGCCTGCCTTTCTCCTGAATTCTGAATTCTGACTCCTGAATTCTGT
Gmet_I301B	(+)	338212	---------CCATGCTGACTACTATATTCCGACTTCTGACTCCTTCAAGGAGAGGTTCAT
Gmet_I302A	(-)	529164	------------------TGCTTTTCTTCTGAATTCTGGCTTCTGAATTCTGGATTCTTG
Gmet_I302B	(+)	529246	------------------CGGTCTTTTCATGA-TGTCTACTTCTGTATTCTGACTCCTGA
Gmet_I303A	(-)	1227156	------------------TTTACCCATTCTGAATTCTGACTCCTGGTTTCTGAATTCTTC
Gmet_I303B	(+)	1227240	-----------------CCGCCTGGCTTCTGAATCCTGAATTCTGACTCCTGACTCCTGG
Gmet_I304A	(-)	3020822	CTGAACTTGTGTTCGAGTGGACTTTCTCCTGAATTCTGACTCCTGAATTCTGTATTCTGA
Gmet_I304B	(+)	3020936	-----AATGCTGTTGCGCGAATTTCATTCCGACTTCTGCATTCTGACTCCTGACTCCCGA
Gmet_I305A	(-)	3473501	-----------------CCTGGTTTTTTCTGGCTCCTGAATTCTGACTCCTGAATACCCA
Gmet_I305B	(+)	3473586	-----------------CATCTTCTATTCTGACTCCTGACTCCTAACTTCTGGATTCTTG
Gmet_I306A	(-)	3513611	------------------ATTGAAAATTCTGGATTCTGAATTCTGACTCCTGACTTCTTT
Gmet_I306B	(+)	3513694	------------------GTCTTTCATTCTGACTGCTGACTCCTGACTTCTGAATTCCAA
Gmet_I307A	(-)	3599790	----------------------AACTTACTGGATTCTGAATTCTGACTCCTGAATTCTTT
Gmet_I307B	(+)	3599865	----------------------TTTCTCCTGAATTCTGACTCCTGTCTCCTGACTACTCA
Gmet_I308A	(-)	3854576	-----------------------AGAACGTGTTGCCTTTCTCCTGAATTCTGACTCCTTG
Gmet_I308B	(+)	3854647	------------------------CCCTCTGC-TTCGCCATCAAACGGCCAGCCCCCTGA
