# Supplementary material for: The genome sequence of Geobacter metallireducens: features of metabolism, physiology and regulation common and dissimilar to Geobacter sulfurreducens
Source: BMC Microbiol. 2009 May 27;9:109. doi: 10.1186/1471-2180-9-109 (PMC2700814; doi:10.1186/1471-2180-9-109)
Supplement: Additional File 10 — Table S5. Cytochrome c biogenesis gene clusters of G. sulfurreducens and G. metallireducens, and associated c-type cytochromes. This table compares the clusters of genes predicted to be involved in biogenesis of c-type cytochromes in G. sulfurreducens and G. metallireducens. [file 1471-2180-9-109-S10.pdf]

Table S5. Cytochrome *c* biogenesis gene clusters of *G. sulfurreducens* and *G. metallireducens*, and associated *c*-type cytochromes.

***G. metallireducens* gene/feature**	***G. sulfurreducens* gene/feature**	**Functional annotation**
**Gene cluster #1**		
Gmet_3231	GSU3282	siroheme synthase N-terminal domain protein
Gmet_3232	GSU3283	ResC/HemX-like cytochrome *c* biogenesis membrane protein
Gmet_3233	GSU3284	*hemA*; glutamyl-tRNA reductase
Gmet_3234	GSU3285	*hemC*; porphobilinogen deaminase
Gmet_3235	GSU3286	uroporphyrinogen III synthase/methyltransferase
Gmet_3236	GSU3287	*tatD*; magnesium-dependent DNase TatD
Gmet_I216A	none	integration host factor binding site, putative
Gmet_I216B	none	integration host factor binding site, putative
Gmet_3237	GSU3288	YgdL family protein
Gmet_3238	GSU3289	ferritin-like domain protein
**Gene cluster #2**		
Gmet_2902	GSU0612	*ppcA*; cytochrome *c*, 3 heme-binding sites
Gmet_2901	GSU0613	ResB-like family protein (cytochrome *c* biogenesis)
Gmet_2900	GSU0614	ResC/HemX-like cytochrome *c* biogenesis membrane protein
Gmet_2899	GSU0615	cytochrome *c*, 10-11 heme-binding sites
Gmet_2898	GSU0616	cytochrome *c*, 8 heme-binding sites
Gmet_H438	none	four heptanucleotide repeats
Gmet_2897	GSU0617	NHL repeat domain protein
none	GSU0617.3	conserved nucleotide sequence
none	GSU0617.1	hypothetical protein
Gmet_3625	none	hypothetical protein
none	GSU0617.2	hypothetical protein
Gmet_3624	none	hypothetical protein
Gmet_2896	GSU0618	*omcE*; cytochrome *c*, 4 heme-binding sites
**Gene cluster #3**[1]		
Gmet_0571	GSU2899?<br>GSU0702?	cytochrome *c*, 26 heme-binding sites
Gmet_0572	GSU2891?<br>GSU0704?<br>GSU2881.1?	ResB-like family protein (cytochrome *c* biogenesis)
Gmet_0573	GSU2890?<br>GSU0705?<br>GSU2880?	ResC/HemX-like cytochrome *c* biogenesis membrane protein
Gmet_0574	GSU0703	conserved hypothetical protein

Gmet_0575	none	cytochrome *c*, 18 heme-binding sites
Gmet_0576	none	cytochrome *c,* 13 heme-binding sites
Gmet_0577	none	conserved hypothetical protein
Gmet_0578	none	ResB-like family protein (cytochrome *c* biogenesis)
Gmet_0579	none	ResC/HemX-like cytochrome *c* biogenesis membrane protein
Gmet_0580	none	cytochrome *c*, 14 heme-binding sites
Gmet_0581	GSU2898	*omcN*; cytochrome *c*, 27-34 heme-binding sites
Gmet_3649	GSU2897	conserved hypothetical protein
Gmet_0582	GSU2896	ankyrin-related protein
none	GSU2895	hypothetical protein
Gmet_0583	none	spermine/spermidine synthase-related protein
Gmet_0584	none	conserved hypothetical protein
Gmet_0585	none	glycosyl transferase, family 9 (heptosyltransferase)
Gmet_0586	none	iron-sulfur oxidoreductase
Gmet_0587	none	radical SAM iron-sulfur oxidoreductase
Gmet_0588	none	conserved hypothetical protein
Gmet_0589	none	glycosyl transferase, family 2
Gmet_0590	none	conserved hypothetical protein
Gmet_0591	none	conserved hypothetical protein
none	GSU2893.1	hypothetical protein
Gmet_0595 (rearranged)	GSU2893	acyl-protein synthetase
none	GSU2892	hypothetical protein
Gmet_0592	GSU2891	ResB-like family protein (cytochrome *c* biogenesis)
Gmet_0593	GSU0703	conserved hypothetical protein
Gmet_0594	GSU2890	ResC/HemX-like cytochrome *c* biogenesis membrane protein
Gmet_0595	GSU2893 (rearranged)	acyl-protein synthetase
Gmet_0596	GSU2889	conserved hypothetical protein
Gmet_0597	GSU2888	radical SAM iron-sulfur oxidoreductase
none	GSU2887.1	hypothetical protein
none	GSU2887	cytochrome *c*, 27 heme-binding sites
Gmet_0598	none	cytochrome *c*, 23 heme-binding sites
Gmet_3587	GSU2886.1	cytochrome *c*, 7 heme-binding sites
none	GSU2885.2	conserved nucleotide sequence (Gmet_R3### family)
none	GSU2885.1	conserved nucleotide sequence (Gmet_R3### family)
none	GSU2885	NHL repeat domain protein

none	GSU2884	*omcA*; cytochrome *c*, 24-27 heme-binding sites
Gmet_0600	none	cytochrome *c*, 19 heme-binding sites
none	GSU2883	*omcH*; cytochrome *c*, 19-24 heme-binding sites
Gmet_0601	none	cytochrome *c*, 7 heme-binding sites
none	GSU2882	*omcG*; cytochrome *c*, 14-18 heme-binding sites
Gmet_0592?<br>Gmet_0572?	GSU2881.1	ResB-like family protein (cytochrome *c* biogenesis)
Gmet_0594?<br>Gmet_0573?	GSU2880	ResC/HemX-like cytochrome *c* biogenesis membrane protein
**Gene cluster 4**		
none	GSU0701	*omcJ*; cytochrome *c*, 6 heme-binding sites
Gmet_0571?<br>Gmet_0575?<br>Gmet_0580?	GSU0702	cytochrome *c*, 26-35 heme-binding sites
Gmet_0574?<br>Gmet_0593?	GSU0703	conserved hypothetical protein
Gmet_0573?<br>Gmet_0594?	GSU0704	ResB-like family protein (cytochrome *c* biogenesis)
Gmet_0572?<br>Gmet_0592?	GSU0705	ResC/HemX-like cytochrome *c* biogenesis membrane protein

[1]Gene cluster 3 in *G. metallireducens* (Gmet_0571 to Gmet_0601) roughly corresponds to two clusters in *G. sulfurreducens* (GSU2899 to GSU2895 and GSU2893.1 to GSU2880), interrupted by GSU2894 with the opposite coding strand. The correspondence is complicated by the rearrangement of GSU2893/Gmet_0595, acquisition/loss of the GSU0703 homologs Gmet_0574 and Gmet_0593, and presence of extra pairs of *c*-type cytochrome biogenesis genes (GSU2881.1-GSU2880; Gmet_0572-Gmet_0573 and Gmet_0578-Gmet_0579) and several other genes in one cluster or the other.
