# Supplementary material for: The genome sequence of Geobacter metallireducens: features of metabolism, physiology and regulation common and dissimilar to Geobacter sulfurreducens
Source: BMC Microbiol. 2009 May 27;9:109. doi: 10.1186/1471-2180-9-109 (PMC2700814; doi:10.1186/1471-2180-9-109)
Supplement: Additional File 11 — Table S6. Transport systems of G. sulfurreducens and G. metallireducens. This table compares the genes predicted to be involved in transport of solutes across the cell membrane and cell wall of G. sulfurreducens and G. metallireducens. [file 1471-2180-9-109-S11.pdf]

Table S6. Transport systems of *G. sulfurreducens* and *G. metallireducens*.

**Transporter substrate**	***G. metallireducens* gene(s)**	***G. sulfurreducens* gene(s)**
Ammonium	Gmet_0695	GSU0940
Ammonium?[1]	Gmet_1738	GSU1221
Quaternary ammonium compounds (efflux protein SugE)	Gmet_2960[2]	GSU0707<br>GSU0708
Potassium (Kup)	Gmet_0038	GSU3346
	Gmet_0039	GSU2485
	none	GSU2350
Potassium (ATPase KdpABCDE)	Gmet_2433-Gmet_2437	GSU2480-GSU2484
Potassium (antiport with proton)	none	GSU1203
Potassium (antiport with proton; KefB)	none	GSU2759
Potassium (antiport with proton; RosB)	Gmet_1105	GSU1204
Potassium? (antiport with proton)	none	GSU0261
Potassium? (TrkA domain)	Gmet_0063	none
Sodium (antiport with proton; MrpABCDEFG complex)	none	GSU2344-GSU2338
Sodium? (antiport with proton)	Gmet_1089	GSU2303
Magnesium/cobalt (CorA-1)[3]	Gmet_1189	GSU1399
Magnesium/cobalt/nickel (CorA-2)[4]	Gmet_0134	GSU3322
Cobalt? (CbiMQO and porin)	Gmet_2441-Gmet_2438	GSU1279-GSU1282
Cobalt? (CbiMQO)	Gmet_0472-Gmet_0475	GSU3004[5];<br>GSU3002-GSU3001
Cobalt/zinc/cadmium? (efflux pump)	Gmet_3071	GSU0487
Cobalt/zinc/cadmium? (efflux pump)	Gmet_0858	GSU2613
Cobalt/zinc/cadmium? (efflux pump)	Gmet_0515	GSU2959
Zinc? (ZIP family)	none	GSU0206
Ferrous iron (FeoB-2)	none	GSU3268
Ferrous iron? (FeoB-1)	Gmet_2444	GSU1380
Manganese? Zinc? Iron? Nickel? (ABC-type)	Gmet_0491-Gmet_0493	GSU2986-GSU2984
Copper (P-type ATPase and membrane proteins)	Gmet_2774-Gmet_2772	GSU2454-GSU2452
Heavy metal? (efflux pump)	Gmet_3507-Gmet_3509	GSU0828-GSU0830
	none	GSU2137-GSU2135
	none	GSU3398-GSU3400
Heavy metal? (ABC-type and protein of unknown function)	Gmet_1556-Gmet_1552	GSU1338[6]-GSU1341
Heavy metal? (ABC-type)	none	GSU0677-GSU0678
Heavy metal efflux? (P-type ATPase)	none	GSU2147
Metal ion (efflux pump and protein of unknown function)	Gmet_1549-Gmet_1546	GSU1330-GSU1333
Cations (E1-E2 family efflux ATPase	Gmet_1614	GSU1678

MgtA)		
Cations (E1-E2 family efflux ATPase)	none	GSU2325
	Gmet_3497	GSU2351
Cations (CorB-type and membrane protein)	Gmet_1153-Gmet_1154	GSU0853-GSU0854
Ion (stomatin-like channel)	Gmet_0813-Gmet_0814	GSU2431-GSU2430
Ions (mechanosensitive channels)	none	GSU1557
	Gmet_1942	GSU1633
	none	GSU1723
	Gmet_2581	GSU2316
	Gmet_2522	GSU2794
Chloride (voltage-gated channel)	Gmet_3470	GSU2363
Phosphate (ABC-type PstSCAB-PhoU)	Gmet_2701-Gmet_2705	GSU1099-GSU1095
Phosphate? (NptA-related sodium/anion symporter)	Gmet_1829	GSU1742
Phosphate? (orthophosphate/polyphosphate-selective porin family)	Gmet_1042	none
Phosphate/sulfate? (PitA and regulator)	Gmet_3132-Gmet_3131	GSU0388-GSU0389
Sulfate (ABC-type CysPTWA)	Gmet_1906-Gmet_1903	GSU1346-GSU1349
Sulfate? (proton/anion symporter)	none	GSU2312
Molybdate (ABC-type ModABC)	Gmet_0512-Gmet_0514	GSU2962-GSU2960
Tungstate (ABC-type TupBA)	Gmet_1041-Gmet_1039	GSU2700-GSU2702
Arsenite (ABC-type, tyrosine phosphatase, ArsR regulator, efflux pump and membrane protein)	Gmet_0524-Gmet_0519	GSU2950-GSU2955
Nitrate (symport with proton; NarK-1)	Gmet_0333	none
Nitrate/nitrite (antiport; NarK-2)	Gmet_0334	none
Chromate	Gmet_2732-Gmet_2731	none
Dicarboxylic acids (*dcuB*)	none	GSU2751
Anion	Gmet_2857	none
L-lactate/glycolate?	none	GSU0226
	none	GSU1622
Formate? (FNT family)	none	GSU0234
Hexuronate?	none	GSU0249
Amino acid? (ABC-type and MarC-related membrane protein)	none	GSU0800-GSU0797
Amino acid? (ABC-type)	none	GSU2649-GSU2651
	none	GSU3406-GSU3404
Branched-chain amino acids? (ABC-type)	Gmet_1824-Gmet_1819	GSU1736[7]-GSU1730
	none	GSU2005-GSU2009
	none	GSU3391-GSU3394
Homoserine/homoserine lactone/threonine? (efflux protein)	none	GSU1194
	Gmet_0798	none
	none	GSU1545

Arginine? (efflux protein)	none	GSU2777
Guanine/hypoxanthine	Gmet_2715	GSU1019
Uracil	none	GSU0932
Aromatic hydrocarbons? (inner and outer membrane proteins and ATPase)	Gmet_1537-Gmet_1534	none
Polyisoprenoids (periplasmic protein YceI)	Gmet_3449	none
Lipid A (ABC-type, MsbA family)	Gmet_2349	GSU2260
3-deoxy-D-manno-octulosonate? (ABC-type YhbNG)	Gmet_1281-Gmet_1282	GSU1889-GSU1888
O-antigen (ABC-2 family)	Gmet_1406-Gmet_1407	GSU1504-GSU1505
O-antigen/teichoic acid? (membrane protein)	Gmet_2012	none
Oligopeptides? (ABC-type)	Gmet_0848-Gmet_0847, Gmet_1234-Gmet_1236	GSU2623-GSU2624, GSU1433-GSU1435
Lipoprotein (ABC-type LolDE)	Gmet_2359-Gmet_2358	GSU2270-GSU2269
	none	GSU2188-GSU2187
	Gmet_1518-Gmet_1517	none
Protease (ABC-type and efflux pump outer membrane and membrane fusion proteins)	none	GSU0278-GSU0276
Exopolysaccharide? (membrane protein)	none	GSU0629
	Gmet_2888	none
Exopolysaccharide? (outer membrane protein)	Gmet_2030	GSU1985
Biopolymer? (TolQRB-TonB-type)	Gmet_3537-Gmet_3540	GSU0028-GSU0025
Biopolymer? (TolQR-type)	Gmet_1459-Gmet_1460	none
Nucleic acids? (uptake proteins ComEA and ComEC)	Gmet_1878, Gmet_1915	GSU1797, GSU1657
Cell division-related (FtsEX, ABC-type)	Gmet_1855-Gmet_1854	GSU1775-GSU1774
Unknown	none	GSU2317
	Gmet_0568-Gmet_0570	GSU2903-GSU2901
Unknown (membrane protein)	none	GSU0265
	none	GSU0268
	none	GSU0752
	none	GSU0758
	Gmet_2550	GSU1016
	Gmet_2050	GSU1998
	none	GSU2677
	Gmet_0563	GSU2907
	none	GSU3407
	Gmet_1490	none
	Gmet_1663	none
Unknown (TerC-related)	Gmet_1155	GSU1570
	Gmet_1782	GSU1336

Unknown (YjgPQ-type)	Gmet_1249-Gmet_1248	GSU1922-GSU1923
Unknown (sodium/solute symporter)	Gmet_2061	none
Unknown (sodium/solute symporter)	Gmet_3031	GSU0518
Unknown[8] (sodium/solute symporters)	Gmet_0739, Gmet_3288	GSU1068, GSU1070, GSU2352
Unknown (membrane importer Tim44 subunit)	Gmet_1927	GSU1091
Unknown (exporter)	Gmet_2183	GSU1855
Unknown (exporter, RND superfamily)	Gmet_2244	none
Unknown (exporter, outer membrane lipoprotein LolB, and carrier/sorter protein LolA)	Gmet_1698, Gmet_3378, Gmet_1699	GSU0457.1-GSU0456
Unknown (drug resistance?)	Gmet_1682	GSU0264
Unknown (DUF6 domain membrane protein)	Gmet_2977	GSU0545
	none	GSU3388
	none	GSU3390
	Gmet_3456	none
Unknown (sodium-driven?)	none	GSU0844
Unknown (ACR3 family)	none	GSU1411
Unknown (TRAP-type)	none	GSU2055-GSU2057
Unknown (TRAP-type solute receptor)	none	GSU2692
Unknown (OstA-type)	Gmet_2474	GSU2367
Unknown (major facilitator superfamily)	none	GSU2491
	none	GSU3264
	Gmet_2251	none
	Gmet_3221	none
Unknown (DUF81 domain membrane protein)	Gmet_1097	none
Unknown (ABC-2 family membrane protein)	Gmet_2545	GSU0922
	Gmet_0014	GSU3455
Unknown (membrane protein and TonB-type)	Gmet_1157-Gmet_1158	GSU0857-GSU0858
Unknown (periplasmic protein)	Gmet_0781	none
	Gmet_0986	GSU2017
Unknown (ABC-type periplasmic protein)	none	GSU1397
	Gmet_1244	GSU1444
	none	GSU1528
	none	GSU2389
	none	GSU3401
	Gmet_2771	none
Unknown (ABC-type periplasmic protein DUF534)	Gmet_1746	GSU1230
Unknown (ligand-gated porin and ABC-type periplasmic protein DUF534)	Gmet_2735-Gmet_2736	GSU0883-GSU0882
Unknown (ABC-type ATP-binding	Gmet_0013	GSU0011

protein)	Gmet_2630	GSU0169
	Gmet_1743	GSU1227
	Gmet_1926	GSU1644
	Gmet_2524	GSU2093
	Gmet_2479	none
Unknown (ABC-type)	Gmet_3423	GSU0093
	Gmet_3416	GSU0101
	none	GSU0211-GSU0212
	Gmet_2543-Gmet_2541	GSU0924-GSU0926
	Gmet_1401-Gmet_1403	GSU1499-GSU1501
	none	GSU2413-GSU2414
	Gmet_0996-Gmet_0998	none
	Gmet_1899-Gmet_1901	none
Unknown (DUF140 ABC-type)	Gmet_2543-Gmet_2541	GSU0924-GSU0926
	Gmet_1271-Gmet_1272	GSU1900-GSU1899
Unknown (DUF140 ABC-type and efflux pump outer membrane protein)	Gmet_0788-Gmet_0784	GSU0817-GSU0813
Unknown (efflux pump YbhG and ABC-type YbhFSR)	Gmet_0659-Gmet_0655	GSU2823-GSU2826
Unknown (ABC-type and membrane protein)	none	GSU1260-GSU1262
Unknown (ABC-type and efflux pump membrane fusion protein)	Gmet_3038-Gmet_3036	GSU0496-GSU0496.2
	Gmet_2408-Gmet_2405	GSU1161-GSU1164
	none	GSU2687-GSU2685
Unknown (ABC-type and efflux pump outer membrane and membrane fusion proteins)	none	GSU0950-GSU0947
	Gmet_3465-Gmet_3460	none
Unknown (outer membrane receptor)	Gmet_1245	GSU1445
Unknown (ligand-gated porin)	Gmet_1669	none
Unknown (TonB-dependent ligand-gated receptor)	none	GSU2982-GSU2981
Unknown (efflux pump outer membrane protein)	Gmet_1190	none
Unknown (efflux pump EmrAB and outer membrane protein)	Gmet_1376-Gmet_1374	GSU1482-GSU1480
Unknown (efflux pump)	Gmet_3129-Gmet_3127	GSU0391-GSU0392, GSU0394
	Gmet_1650-Gmet_1652	GSU1609-GSU1611
	Gmet_2519-Gmet_2517	GSU2665-GSU2664[9]
	Gmet_0809-Gmet_0811	GSU2697-GSU2695
	none	GSU2781-GSU2782
	Gmet_1666-Gmet_1664	none
Unknown (sodium-driven efflux pump)	Gmet_2508	GSU2653
	Gmet_0358	GSU3129
Unknown (porin)	Gmet_0802	GSU0767

	Gmet_0532	GSU2939
	none	GSU3271
	Gmet_0783	none
	Gmet_1101	none
	Gmet_1194-Gmet_1193	none
	Gmet_1200	none
	Gmet_1649	none
	Gmet_1995	none
	Gmet_1998	none
	Gmet_2192	none
	Gmet_2624	none
	Gmet_2831	none
Unknown (porin OmpJ and paralog)	Gmet_3254	GSU3304, GSU3403

[1]The alignment with known ammonium transporters is unsatisfactory.
[2]Duplicate transporter genes GSU0707 and GSU0708 correspond to a single gene, Gmet_2960, inactivated by an internal stop codon.
[3]*Thermotoga*-type CorA protein.
[4]Enteric-type CorA protein.
[5]The two domains of CbiM represented by Gmet_0472 and Gmet_0473 are fused in GSU3004.
[6]Heavy metal-binding domain protein GSU1338 is duplicated as Gmet_1556 and Gmet_1555.
[7]*G. sulfurreducens* and other *Geobacteraceae* have duplicate periplasmic amino acid-binding protein genes (GSU1734-GSU1735); *G. metallireducens* has only Gmet_1823, a GSU1734 ortholog.
[8]These symporters are very closely related to each other, and co-transcribed with members of an uncharacterized protein family.
[9]The inner membrane protein and outer membrane protein represented by Gmet_2518 and Gmet_2517 are fused in GSU2664, suggesting that they must be proteolytically separated to accommodate the membrane fusion protein GSU2665 (Gmet_2519).
