# Supplementary material for: The genome sequence of Geobacter metallireducens: features of metabolism, physiology and regulation common and dissimilar to Geobacter sulfurreducens
Source: BMC Microbiol. 2009 May 27;9:109. doi: 10.1186/1471-2180-9-109 (PMC2700814; doi:10.1186/1471-2180-9-109)
Supplement: Additional File 12 — Table S7. Sensor histidine kinases (HATPase_c domain proteins), REC domain-containing proteins, and transcriptional regulators of G. metallireducens. This table compares the genes predicted to be involved in two-component signalling and transcriptional regulation in G. sulfurreducens and G. metallireducens. [file 1471-2180-9-109-S12.pdf]

Table S7. Sensor histidine kinases (HATPase_c domain proteins), response receivers (REC domain proteins), and transcriptional regulators of *G. metallireducens*.

**Gene**	**GSU match**	**Specific Annotation and Domain Architecture**
	**Sensor histidine kinases**	
Gmet_0009	GSU0007	HAMP, PAS, HisKA, HATPase_c
Gmet_0010	GSU0008	REC, PAS, HisKA, HATPase_c
Gmet_0011	GSU0009	PAS, HisKA, HATPase_c
Gmet_0012	GSU0010	REC, unknown, HisKA, HATPase_c
Gmet_0043	GSU3350	HisKA, HATPase_c, REC, GGDEF
Gmet_0077	none	HAMP, HisKA, HATPase_c, REC
Gmet_0197	GSU0144	PAS, GAF, HisKA, HATPase_c
Gmet_0202	GSU0149	REC, HisKA, HATPase_c
Gmet_0350	GSU3138	REC, HisKA, HATPase_c
Gmet_0500	none	PAS, HisKA, HATPase_c
Gmet_0527	GSU2947	HAMP, HisKA, HATPase_c
Gmet_0554	GSU2916	HAMP, HisKA, HATPase_c (nonconserved heme-binding site)
Gmet_0666	GSU2815	PAS, PAS, HisKA, HATPase_c
Gmet_0687	none	HisKA, HATPase_c
Gmet_0696	GSU0941	HisKA, HATPase_c
Gmet_0701	GSU0962	HAMP, HisKA, HATPase_c
Gmet_0713	GSU0475	PAS, PAC, HisKA, HATPase_c
Gmet_0731	none	PAS, PAC, HisKA, HATPase_c
Gmet_0777	GSU0812	nitrogen regulation sensor histidine kinase NtrY (HAMP, PAS, HisKA, HATPase_c)
Gmet_0875	none	REC, HisKA, HATPase_c
Gmet_0960	GSU2042	HAMP, HisKA, HATPase_c
Gmet_1005	none	FhlA, PAS, PAC, HisKA, HATPase_c
Gmet_1027	none	HAMP, HisKA, HATPase_c
Gmet_1054	none	FhlA, HisKA, HATPase_c
Gmet_1057	GSU0775	HAMP, HisKA, HATPase_c
Gmet_1080	none	CheA
Gmet_1083	none	PAS, PAS, PAC, HisKA, HATPase_c
Gmet_1212	none	PAS, HisKA, HATPase_c
Gmet_1218	none	PAS, PAC, PAS, PAC, AtoS, HisKA, HATPase_c
Gmet_1219	none	REC, HisKA, HATPase_c
Gmet_1243	GSU1443	HAMP, HisKA, HATPase_c, REC
Gmet_1292	GSU1878	HisKA, HATPase_c
Gmet_1379	none	HATPase_c
Gmet_1396	GSU1494	HisKA, HATPase_c
Gmet_1667	none	HAMP, HATPase_c
Gmet_1670	none	REC, HisKA, HATPase_c
Gmet_1707	none	PAS, PAS, PAC, HisKA, HATPase_c, REC
Gmet_1765	GSU1265	incomplete HisKA, HATPase_c, REC

Gmet_1918	GSU1655	REC, PAS, PAC, HisKA, HATPase_c, REC
Gmet_1945	GSU1630	HAMP, PAS, HisKA, HATPase_c
Gmet_1991	GSU1939	GAF, HisKA, HATPase_c, GAF, HD
Gmet_1993	GSU1941	GAF, HATPase_c
Gmet_2035	GSU1990	NtrB-related (HisKA, HATPase_c)
Gmet_2146	none	HisKA, HATPase_c
Gmet_2159	none	PAS, HATPase_c
Gmet_2186	none	REC, HisKA, HATPase_c
Gmet_2287	GSU2189	HATPase_c (nonconserved heme-binding site)
Gmet_2311	GSU2222	CheA
Gmet_2426	GSU1302	HAMP, HisKA, HATPase_c, REC (1 heme-binding site)
Gmet_2427	GSU1290	CheA
Gmet_2436	GSU2483	osmosensitive potassium channel sensor histidine kinase KdpD (HisKA, HATPase_c)
Gmet_2454	GSU1319	HisKA, HATPase_c
Gmet_2460	none	HATPase_c
Gmet_2540	none	HATPase_c
Gmet_2562	GSU1004	PAS, HATPase_c
Gmet_2639	none	REC, HisKA, HATPase_c
Gmet_2642	none	GAF, HATPase_c, REC, REC, REC
Gmet_2677	GSU1119	REC, HisKA, HATPase_c
Gmet_2694	GSU1101	HAMP, PAS, HisKA, HATPase_c
Gmet_2698	none	GAF, HisKA, HATPase_c
Gmet_2710	none	HPT, HATPase_c, CheAreg, REC
Gmet_2712	none	REC, HisKA, HATPase_c
Gmet_2737	GSU0881	HAMP, HisKA, HATPase_c
Gmet_2748	GSU2384	GAF, HisKA, HATPase_c
Gmet_2762	GSU1036	Chase, HisKA, ATPase
Gmet_2770	none	HisKA, HATPase_c
Gmet_2788	none	PBPb, HisKA, HATPase_c
Gmet_2790	none	REC, HisKA, HATPase_c
Gmet_2811	GSU2297	Cache, HAMP, PAS, PAC, HisKA, HATPase_c
Gmet_2824	none	PAS, GAF, HATPase_c, REC
Gmet_2923	GSU0599	HAMP, HisKA, HATPase_c (2 heme-binding sites)
Gmet_2956	none	PAS, FhlA, FhlA, HisKA, HATPase_c
Gmet_2998	none	HisKA, HATPase_c, REC
Gmet_3157	GSU0373	HAMP, HisKA, HATPase_c
Gmet_3180	GSU3252	GAF, HisKA, HATPase_c
Gmet_3266	GSU0296	CheA
Gmet_3279	GSU0283	GAF, GAF, HisKA, HATPase_c
Gmet_3382	GSU0452	HAMP, HisKA, HATPase_c
Gmet_3432	none	HisKA, HATPase_c
Gmet_3455	none	PAS, HisKA, HATPase_c
Gmet_3474	none	HATPase_c
Gmet_3477	none	HAMP, PAS, PAS, PAS, PAS, HATPase_c, REC, PAS

Gmet_3478	none	HAMP, PAS, PAC, PAS, HATPase_c
**REC domain-containing proteins**		
Gmet_0010	GSU0008	REC, PAS, HisKA, HATPase_c
Gmet_0012	GSU0010	REC, unknown, HisKA, HATPase_c
Gmet_0043	GSU3350	HisKA, HATPase_c, REC, GGDEF
Gmet_0069	GSU3376	REC, GGDEF
Gmet_0077	none	HAMP, HisKA, HATPase_c, REC
Gmet_0202	GSU0149	REC, HisKA, HATPase_c
Gmet_0350	GSU3138	REC, HisKA, HATPase_c
Gmet_0501	GSU2535	REC
Gmet_0555	GSU2524	REC, sigma-54 interaction, HTH8
Gmet_0660	GSU2822	REC, ANTAR
Gmet_0688	none	REC, transregC
Gmet_0702	GSU0963	REC, sigma-54 interaction, HTH8
Gmet_0762	GSU1090	REC
Gmet_0776	GSU0811	nitrogen response regulator NtrX (REC, sigma-54 interaction, HTH8)
Gmet_0778	none	CheY-11
Gmet_0812	GSU1129	REC, sigma-54 interaction
Gmet_0875	none	REC, HisKA, HATPase_c
Gmet_0945	GSU2062	REC, incomplete GGDEF
Gmet_0955	GSU2046	REC
Gmet_0961	GSU2041	REC, sigma-54 interaction, HTH8
Gmet_1026	none	REC, transregC
Gmet_1055	none	REC, sigma-54 interaction, HTH8
Gmet_1058	GSU0776	REC, sigma-54 interaction, HTH8
Gmet_1075	none	chemotaxis protein-glutamate methylesterase CheB-6 (REC, CheB_methylest)
Gmet_1082	GSU1250	REC, sigma-54 interaction, HTH8
Gmet_1219	none	REC, HisKA, HATPase_c
Gmet_1220	GSU1417	REC
Gmet_1243	GSU1443	HAMP, HisKA, HATPase_c, REC
Gmet_1291	GSU1879	REC, transregC
Gmet_1378	none	REC
Gmet_1397	GSU1495	REC, sigma-54 interaction, HTH8
Gmet_1551	none	REC
Gmet_1607	GSU1619	CheY-4
Gmet_1670	none	REC, HisKA, HATPase_c
Gmet_1706	GSU2313	REC, GGDEF
Gmet_1707	none	PAS, PAS, PAC, HisKA, HATPase_c, REC
Gmet_1737	GSU1220	GspIIEN, REC
Gmet_1747	GSU1231	REC-related
Gmet_1765	GSU1265	incomplete HisKA, HATPase_c, REC
Gmet_1914	GSU1658	REC, GGDEF
Gmet_1917	GSU1656	REC, PAS, GGDEF

Gmet_1918	GSU1655	REC, PAS, PAC, HisKA, HATPase_c, REC
Gmet_1919	GSU1654	REC, HD
Gmet_1992	GSU1940	REC, sigma-54 interaction, HTH8
Gmet_2034	GSU1989	REC, sigma-54 interaction
Gmet_2145	none	REC, sigma-54 interaction, HTH8
Gmet_2157	none	REC, HTHaraC
Gmet_2158	GSU3229	REC, HTHluxR
Gmet_2186	none	REC, HisKA, HATPase_c
Gmet_2302	GSU2212	CheY-5
Gmet_2304	GSU2214	chemotaxis protein-glutamate methylesterase CheB-3 (REC, CheB_methylest)
Gmet_2308	GSU2219	CheY-10
Gmet_2312	GSU2223	CheY-6
Gmet_2418	GSU1145	chemotaxis protein-glutamate methylesterase CheB-2 (REC, CheB_methylest)
Gmet_2426	GSU1302	HAMP, HisKA, HATPase_c, REC (1 heme-binding site)
Gmet_2428	GSU1289	CheY-3
Gmet_2431	GSU1286	CheY-2
Gmet_2437	GSU2484	osmosensitive potassium channel response regulator KdpE (REC, transregC)
Gmet_2453	GSU1320	REC, sigma-54 interaction, HTH8
Gmet_2563	GSU1003	REC, sigma-54 interaction, HTH8
Gmet_2639	none	REC, HisKA, HATPase_c
Gmet_2642	none	GAF, HATPase_c, REC, REC, REC
Gmet_2677	GSU1119	REC, HisKA, HATPase_c
Gmet_2679	GSU1117	REC
Gmet_2693	GSU1102	REC, transregC
Gmet_2710	none	HPT, HATPase_c, CheAreg, REC
Gmet_2711	none	chemotaxis protein-glutamate methylesterase CheB-8 (REC, CheB_methylest)
Gmet_2712	none	REC, HisKA, HATPase_c
Gmet_2739	GSU0879	CheV (CheW, REC)
Gmet_2789	none	REC
Gmet_2790	none	REC, HisKA, HATPase_c
Gmet_2818	GSU0700	REC, PAS, PAC, PAS, PAC, PP2C
Gmet_2824	none	PAS, GAF, HATPase_c, REC
Gmet_2827	GSU2756.2	CheY-8
Gmet_2914	GSU0596	REC
Gmet_2924	GSU0598	REC, sigma-54 interaction, HTH8
Gmet_2926	GSU0596	REC
Gmet_2998	none	HisKA, HATPase_c, REC
Gmet_2999	GSU1891	REC, HDc
Gmet_3117	GSU0405	REC
Gmet_3119	GSU0403	CheY-1
Gmet_3158	GSU0372	REC, sigma-54 interaction, HTH8

Gmet_3179	GSU3253	REC
Gmet_3211	GSU3198	CheY-7
Gmet_3218	GSU3261	REC
Gmet_3220	GSU0877	REC, PilZ
Gmet_3263	GSU0298, GSU300 (fragments)	REC, sigma-54 interaction
Gmet_3269	GSU2093	chemotaxis protein-glutamate methylesterase CheB-1 (REC, CheB_methylest)
Gmet_3383	GSU0451	REC, transregC
Gmet_3414	GSU0104	REC
Gmet_3472	none	REC
Gmet_3473	none	REC, PulE
Gmet_3476	none	REC, HDc
Gmet_3477	none	HAMP, PAS, PAS, PAS, PAS, HATPase_c, REC, PAS
**Transcriptional Regulators lacking REC domains**		
Gmet_0055	GSU3363	iron-sulfur cluster-binding transcriptional regulator (FehydlgC, FeS, sigma-54 interaction, HTH8)
Gmet_0056	GSU3364	transcriptional regulator, CopG family
Gmet_0062	GSU3370	transcriptional regulator, GntR family
Gmet_0088	GSU3031	sigma-54 dependent transcriptional regulator
Gmet_0092	GSU3421	transcriptional regulator, Crp/Fnr family
Gmet_0124	GSU3329	predicted DNA-binding protein with helix-hairpin-helix motif
Gmet_0130	GSU3324	helix-turn-helix transcriptional regulator, LexA-related
Gmet_0186	GSU2817	transcriptional regulator, LysR family
Gmet_0200	GSU0147	regulatory protein RecX
Gmet_0215	GSU0164	conserved hypothetical protein
Gmet_0237	GSU0191	cold shock DNA/RNA-binding domain protein
Gmet_0240	GSU0187	helix-turn-helix and Zn ribbon transcriptional regulator
Gmet_0315	none	transcriptional regulator, XRE family
Gmet_0317	none	transcriptional regulator
Gmet_0339	GSU0359	sigma-54-dependent sensor DNA-binding regulator (PAS, sigma-54 interaction, HTH8)
Gmet_0340	none	cold shock DNA/RNA-binding domain protein
Gmet_0359	none	transcriptional regulator, MarR family
Gmet_0370	GSU3109	transcriptional regulator, IclR family
Gmet_0420	GSU3060	transcriptional regulator, TetR family
Gmet_0436	GSU3045	negative regulator of flagellin synthesis FlgM
Gmet_0490	GSU2987.1	predicted DNA-binding protein with PD1-like DNA-binding motif
Gmet_0494	GSU2980	metal-binding domain transcriptional regulator
Gmet_0522	GSU2952	transcriptional regulator, ArsR family
Gmet_0528	GSU2946	DNA-binding heavy metal response regulator
Gmet_0530	GSU2941	transcriptional regulator, LysR family

Gmet_0741	GSU1072	transcriptional regulator, IclR family
Gmet_0750	GSU1992	transcriptional regulator, Crp/Fnr family
Gmet_0763	GSU2475	GAF sensor sigma-54-dependent transcriptional regulator (GAF, GAF, sigma-54 interaction)
Gmet_0803	GSU0770	transcriptional regulator, TetR family
Gmet_0808	GSU2698	transcriptional regulator, TetR family
Gmet_0846	GSU0399	transcriptional regulator, ArsR family
Gmet_0871	GSU2571	Rrf2 family transcriptional regulator
Gmet_0901	GSU2540	transcriptional regulator, XRE family
Gmet_0970	GSU2033	transcriptional regulator, XRE family
Gmet_1007	GSU1727	zinc finger transcriptional regulator, TraR/DksA family
Gmet_1023	none	conserved hypothetical protein
Gmet_1051	none	predicted transcriptional regulator
Gmet_1084	GSU2523	transcriptional regulator, LysR family
Gmet_1121	none	transcriptional regulator, GntR family
Gmet_1127	GSU2716	sugar fermentation stimulation protein
Gmet_1161	GSU0861	conserved hypothetical protein
Gmet_1221	GSU1419	helix-turn-helix DNA-binding protein, putative
Gmet_1305	GSU1863	transcriptional regulator, Ros/MucR family
Gmet_1339	none	transcriptional regulator, putative
Gmet_1418	GSU1522	transcriptional regulator, MerR family
Gmet_1439	GSU0041<br>GSU1617	LexA repressor
Gmet_1513	none	transcriptional regulator, LuxR family
Gmet_1520	none	transcriptional regulator, TetR family
Gmet_1542	none	sigma-54-dependent transcriptional activator of aromatic compound catabolism (XylRN, V4R, sigma-54 interaction, HTH8)
Gmet_1609	none	LexA-ImpB/MucB/SamB protein pseudogene fusion
Gmet_1639	GSU1702	ROK domain transcriptional regulator/sugar kinase
Gmet_1684	GSU0266	transcriptional regulator, LysR family
Gmet_1724	GSU2779	transcriptional regulator, MerR family
Gmet_1731	GSU0175	transcriptional regulator, TetR family
Gmet_1768	GSU1270	pyrimidine operon regulatory protein PyrR; uracil phosphoribosyltransferase
Gmet_1784	none	helix-turn-helix DNA-binding protein, putative
Gmet_1791	none	helix-turn-helix DNA-binding protein, putative
Gmet_1895	GSU2587	transcriptional regulator, MarR family
Gmet_1907	GSU1345	transcriptional regulator, Rrf2 family
Gmet_1931	GSU1639	transcriptional regulator, putative
Gmet_1986	GSU1934	Baf-related transcriptional regulator, putative
Gmet_1987	GSU1935	biotin operon repressor and biotin--acetyl-CoA carboxylase ligase
Gmet_2055	none	sigma-54-dependent sensor DNA-binding regulator (PAS, sigma-54 interaction, HTH8)

Gmet_2064	none	transcriptional regulator, IclR family
Gmet_2097	none	transcriptional regulator, putative
Gmet_2099	none	aromatic catabolism-like transcriptional regulator (XylR, V4R, sigma-54 interaction, HTH8)
Gmet_2109	none	sigma-54-dependent sensor DNA-binding regulator (PAS, sigma-54 interaction, HTH8)
Gmet_2114	none	aromatic catabolism-like sigma-54-dependent transcriptional regulator (XylR, V4R, sigma-54 interaction, HTH8)
Gmet_2116	none	aromatic catabolism-like sigma-54-dependent transcriptional regulator (XylR, V4R, sigma-54 interaction, HTH8)
Gmet_2133	none	transcriptional regulator, MarR family
Gmet_2216	none	transcriptional regulator, TetR family
Gmet_2223	none	sigma 54-dependent transcriptional regulator FhlA (GAF, sigma-54 interaction, HTH8)
Gmet_2241	none	transcriptional regulator, TetR family
Gmet_2259	none	transcriptional regulator, IclR family
Gmet_2277	none	helix-turn-helix DNA-binding protein, putative
Gmet_2442	GSU1382	iron/manganese-dependent transcriptional regulator
Gmet_2445	GSU1379	ferric uptake regulation protein Fur
Gmet_2491	none	transcriptional regulator, frameshifted
Gmet_2534	GSU1542	transcriptional regulator, putative
Gmet_2645	none	transcriptional regulator, TetR family
Gmet_2681	GSU1115	transcriptional regulator, putative
Gmet_2697	none	transcriptional regulator, LytR/AlgR family
Gmet_2779	GSU2460	membrane-bound transcriptional regulator (ribonuclease BN-related)
Gmet_2805	none	ROK domain transcriptional regulator/sugar kinase
Gmet_2889	GSU0625	helix-turn-helix transcriptional regulator, putative
Gmet_2950	none	helix-turn-helix transcriptional regulator fragment
Gmet_2989	GSU0534	transcriptional regulator, BadM/Rrf2 family
Gmet_3027	GSU0514	transcriptional regulator, IclR family
Gmet_3075	GSU0483	adenosine nucleotide-binding predicted transcriptional regulator ExsB
Gmet_3086	GSU0473	helix-turn-helix transcriptional regulator, putative
Gmet_3164	GSU0366	transcriptional regulator, XRE family
Gmet_3240	GSU3292	transcriptional regulator, Fur family
Gmet_3247	GSU3298	transcriptional regulator with cupin-like beta-barrel domain, putative
Gmet_3278	GSU0284	zinc finger transcriptional regulator, TraR/DksA family
Gmet_3285	none	sigma-54 dependent sensor DNA-binding regulator (PAS-like, sigma-54 interaction, HTH8)
Gmet_3286	none	transcriptional regulator, IclR family
Gmet_3308	none	sigma-54 dependent DNA-binding transcriptional

		regulator (sigma-54 interaction, HTH8)
Gmet_3412	GSU0107	ParB-like nuclease domain protein, possible transcriptional regulator
Gmet_3438	GSU0079	helix-turn-helix transcriptional regulator with cupin domain
Gmet_3450	none	winged-helix transcriptional regulator, MarR family
Gmet_3466	GSU0951	transcriptional regulator, TetR family
Gmet_3534	GSU0031	heat-inducible transcription repressor HrcA
Gmet_3554	GSU3457	amino acid-binding ACT domain regulatory protein

Note: proteins with both HATPase_c domains and REC domains appear in both lists.
