# Supplementary material for: The genome sequence of Geobacter metallireducens: features of metabolism, physiology and regulation common and dissimilar to Geobacter sulfurreducens
Source: BMC Microbiol. 2009 May 27;9:109. doi: 10.1186/1471-2180-9-109 (PMC2700814; doi:10.1186/1471-2180-9-109)
Supplement: Additional File 13 — Table S8. Diguanylate cyclases (GGDEF domain proteins) of G. sulfurreducens and G. metallireducens. This table compares the genes predicted to produce the intracellular messenger cyclic diguanylate in G. sulfurreducens and G. metallireducens. [file 1471-2180-9-109-S13.pdf]

Table S8. Diguanylate cyclases (GGDEF domain proteins) of *G. sulfurreducens* and *G. metallireducens*.

Gene	GSU match	Specific Annotation and Domain Architecture
Gmet_0043	GSU3350	sensor histidine kinase response receiver diguanylate cyclase (HisKA, HATPase_c, REC, GGDEF)
Gmet_0049	none	diguanylate cyclase (GGDEF)
Gmet_0069	GSU3376	response receiver-modulated diguanylate cyclase (REC, GGDEF)
Gmet_0700	GSU0946	sensor diguanylate cyclase/phosphoesterase (HAMP, PAS, GGDEF, EAL)
Gmet_0773	GSU0808	diguanylate cyclase (CAP_ED, GGDEF)
Gmet_0782	none	diguanylate cyclase/phosphoesterase (GGDEF, EAL)
Gmet_0840	GSU2632	diguanylate cyclase (GGDEF)
Gmet_0945	GSU2062	putative response receiver-modulated nucleotide cyclase (REC, GGDEF-related)
Gmet_0957	GSU2044	sensor diguanylate cyclase/phosphoesterase (GAF, GGDEF, EAL)
Gmet_0987	GSU2016	sensor diguanylate cyclase/phosphoesterase (PAS, GGDEF, EAL)
Gmet_1298	GSU1870	sensor diguanylate cyclase (GAF, GGDEF)
Gmet_1706	GSU2313	response receiver-modulated diguanylate cyclase (REC, GGDEF)
Gmet_1914	GSU1658	response receiver-modulated diguanylate cyclase (REC, GGDEF)
Gmet_1917	GSU1656	response receiver sensor diguanylate cyclase (REC, PAS, GGDEF)
Gmet_1989	GSU1937	putative nucleotide cyclase (HAMP, GGDEF-related)
Gmet_2425	none	diguanylate cyclase (GGDEF)
Gmet_2629	none	sensor diguanylate cyclase/phosphoesterase (GGDEF, EAL)
Gmet_2721	GSU0895	sensor diguanylate cyclase (GAF, GGDEF)
Gmet_2982	GSU0542	diguanylate cyclase (GGDEF)
Gmet_3085	GSU0474	sensor diguanylate cyclase/phosphoesterase (PAS, PAS, PAC, PAS, PAC, GGDEF, EAL)
none	GSU0537	sensor diguanylate cyclase/phosphoesterase (PAS, GGDEF, EAL)
none	GSU0952	diguanylate cyclase (GGDEF)
none	GSU1037	response receiver-modulated diguanylate cyclase/phosphoesterase (REC, GGDEF, EAL)
none	GSU1399.1	sensor diguanylate cyclase (PAS, GGDEF)
none	GSU1554	diguanylate cyclase (GGDEF)
none	GSU1643	response receiver-modulated diguanylate cyclase (REC, GGDEF)
none	GSU1671	response receiver-modulated diguanylate cyclase (REC,

		GGDEF)
none	GSU1927	response receiver sensor diguanylate cyclase/phosphoesterase (REC, PAS, GGDEF, EAL)
none	GSU2511	sensor diguanylate cyclase/phosphoesterase (CHASE4, GGDEF, EAL)
none	GSU2534	response receiver sensor diguanylate cyclase (REC, PAS, GGDEF)
none	GSU2828	diguanylate cyclase (GGDEF)
none	GSU2969	sensor diguanylate cyclase (PAS, GGDEF)
none	GSU3356	diguanylate cyclase (HAMP, GGDEF)
