# Supplementary material for: The genome sequence of Geobacter metallireducens: features of metabolism, physiology and regulation common and dissimilar to Geobacter sulfurreducens
Source: BMC Microbiol. 2009 May 27;9:109. doi: 10.1186/1471-2180-9-109 (PMC2700814; doi:10.1186/1471-2180-9-109)
Supplement: Additional File 14 — Table S9. Chemotaxis-type signalling proteins of G. sulfurreducens and G. metallireducens. This table compares the genes predicted to participate in chemotaxis-type signalling in G. sulfurreducens and G. metallireducens. [file 1471-2180-9-109-S14.pdf]

Table S9. Chemotaxis-type signalling proteins of *G. sulfurreducens* and *G. metallireducens*.

**Locus Tag**	***G. sulfurreducens* gene**	***G. metallireducens* gene**
**Methyl-accepting chemotaxis proteins**		
mcp001	GSU0400	Gmet_2825[1]
mcp002	GSU0401	none
mcp003	GSU0582	Gmet_2939
mcp004	GSU0583	Gmet_2938
mcp005	GSU0683	Gmet_2424[1]
mcp006	GSU0750	none
mcp007	GSU0756	none
mcp008	GSU0766	none
mcp009	GSU0916	none
mcp010	GSU0935	none
mcp011	GSU1029	Gmet_0712
mcp012	GSU1030	Gmet_2825[1]
mcp013	GSU1032	none
mcp014	GSU1033	Gmet_0821[1]
mcp015	GSU1033.1	Gmet_0821[1]
mcp016	GSU1035	none
mcp017	GSU1041	none
mcp018	GSU1140	Gmet_2423
mcp019	GSU1141	none
mcp020	GSU1287	Gmet_2430
mcp021	GSU1294	none
mcp022	GSU1298	Gmet_2424[1]
mcp023	GSU1300	Gmet_2424[1]
mcp024	GSU1303	none
mcp025	GSU1304	none
mcp026	GSU1374	none
mcp027	GSU1704	Gmet_1641
mcp028	GSU2372	Gmet_2478
mcp029	GSU2423	Gmet_3324
mcp030	GSU2579	none
mcp031	GSU2652	Gmet_0799
mcp032	GSU2942	Gmet_0529
mcp033	GSU3156	none
mcp034	GSU3196	none
mcp035	none	Gmet_1078
mcp036	none	Gmet_2422
mcp037	none	Gmet_2709
mcp038	none	Gmet_2828
mcp039	none	Gmet_3087
**Chemotaxis sensor histidine kinases**		

cheA-1	GSU1290	Gmet_2427
cheA-2	GSU2222	Gmet_2311
cheA-3	GSU3199	none
cheA-4	GSU0296	Gmet_3266
cheA-5	none	Gmet_1080
cheA-6	none	Gmet_2710[2]
**Chemotaxis methylesterases**		
cheB-1	GSU0293	Gmet_3269
cheB-2	GSU1145	Gmet_2418
cheB-3	GSU2214	Gmet_2304
cheB-4	GSU2756.1	Gmet_2826
cheB-5, cheR-5	none	Gmet_0780
cheB-6	none	Gmet_1075
cheB-7	none	Gmet_2640
cheB-8	none	Gmet_2711
**Chemotaxis response regulator phosphatases**		
cheC-1	GSU3200	Gmet_3210
cheC-2	GSU0404	Gmet_3118
cheC-3	GSU1618	Gmet_1606
**Chemotaxis complex proteins CheD**		
cheD-1	GSU0726	none
cheD-2	GSU1144	Gmet_2419
cheD-3	GSU3201	Gmet_3209
cheD-4	none	Gmet_1076
**Chemotaxis methyltransferases**		
cheR-1	GSU0295	Gmet_3267
cheR-2	GSU1143	Gmet_2420
cheR-3	GSU2215	Gmet_2305
cheR-4	GSU3195	Gmet_3212
cheR-5, cheB-5	none	Gmet_0780
cheR-6	none	Gmet_1077
cheR-7	none	Gmet_2641
cheR-8	none	Gmet_2707
cheR-9	GSU0291	Gmet_3271
**Chemotaxis complex proteins CheV/CheW**		
cheV	GSU0879	Gmet_2739
cheW-1	GSU0297	Gmet_3265
cheW-2	GSU0684	none
cheW-3	GSU1142	Gmet_2421
cheW-4	GSU1299	none
cheW-5	GSU1301	none
cheW-6	GSU2218	Gmet_2307
cheW-7	GSU2220	Gmet_2309
cheW-8 (mvhQ)	GSU2416	Gmet_3317
cheW-9	GSU2578	none

cheW-10	GSU3197	none
cheW-11	none	Gmet_1079
cheW-12	none	Gmet_2706
cheW-13	none	Gmet_2708
**Chemotaxis response regulators**		
cheY-1	GSU0403	Gmet_3119
cheY-2	GSU1286	Gmet_2431
cheY-3	GSU1289	Gmet_2428
cheY-4	GSU1619	Gmet_1607
cheY-5	GSU2212	Gmet_2302
cheY-6	GSU2223	Gmet_2312
cheY-7	GSU3198	Gmet_3211
cheY-8	GSU2756.2	Gmet_2827
cheY-9	GSU2217	none
cheY-10	GSU2219	Gmet_2308
cheY-11	none	Gmet_0778

[1]Gene duplication in *G. sulfurreducens* makes it impossible to assign an ortholog of the *G. metallireducens* gene.
[2]Gmet_2710 encodes a CheA protein with an internal REC domain.
