# Supplementary material for: The genome sequence of Geobacter metallireducens: features of metabolism, physiology and regulation common and dissimilar to Geobacter sulfurreducens
Source: BMC Microbiol. 2009 May 27;9:109. doi: 10.1186/1471-2180-9-109 (PMC2700814; doi:10.1186/1471-2180-9-109)
Supplement: Additional File 15 — Figure S6. Predicted global regulator binding sites (class 4). This is an alignment of 20 DNA sequences that were matched by nucleotide-level BLAST. Each site appears to be based on a pentanucleotide repeat (consensus CCYTC) that occurs four times on one strand and twice on the other. The sequence strand and start and stop nucleotide positions are indicated. [file 1471-2180-9-109-S15.pdf]

Gmet_I401	(+)	346972	347013	C C C T C A C C C G C C C T T - C G G G C A C C C T C T C C C C G G G G A G A G G G
Gmet_I402	(+)	847962	847996	C C C T C G C C C C C A G - - - - - - - - C C C C T C T C A C C G A G G G C G A G G G
Gmet_I403	(-)	848007	848048	C C C T C A C C C G G C C T A - C G G C C A C C C T C T C C C C G A G G G A G G G G T
Gmet_I404	(-)	2261974	2262015	C C C T C A C C C G C C C T T - C G G G C A C C C T C T C C C T G G G G G A G A G G G
Gmet_I405	(+)	2262033	2262068	C C C C C T C A C - - C C C T - A - - - - C C C C T C T C C C T C A G G G A G A G G G
Gmet_I406	(-)	2434919	2434960	C G T T C A C C C G G C C T T - C G G C C A C C C T C T C C C A C G G C G G G G A G A
Gmet_I407	(+)	2930517	2930549	C C C T C A C C C - - - - - - - - - - C A A C C C T C T C C C G G A G G G A G A G G G
Gmet_I408	(-)	2930561	2930602	C C C T C A C C T G G C C T T - C G G C C A C C C T C T C C C G G A G G G C G A G G G
Gmet_I409	(-)	2931995	2932036	C C C T C A C C C G G C C T C - C G G C C A C C C T C T C C C C A A G G G C G A G G G
Gmet_I410	(+)	3068138	3068180	C C C T C A C C C G G C C T T G C G G C C A C C C T C T C C C C C G G G G A G A G G G
Gmet_I411	(+)	3075684	3075725	C C C T C A C C C G A C C T T - C G G C C A C C C T C T C C C C A A G G G A G A G G G
Gmet_I412	(-)	3089494	3089535	C C C T C A C C C G C C C T C - C G G G C A C C C T C T C C C A C G G G G A G A G G G
Gmet_I413	(+)	3476916	3476957	C C C T C A C C C G G C C T T - C G G C C A C C C T C T C C C C C A A G G A G A G G G
Gmet_I414	(-)	3476969	3477004	C C C C T T C A C - - C C C C - - - - - G C C C C T C T C C C T C A G G G C G A G G G
Gmet_I415	(+)	3811572	3811613	C C C T C A C C C G G C C T T - C G G C C A C C C T C T C C C T G A G G G A G A G G G
Gmet_I416	(-)	3831391	3831432	C C C T C A C C C G G C C T T - C G G C C A C C C T C T C C C T C C A G G C G G G G G
Gmet_I417	(-)	3930148	3930182	C C C C C T C A C - - - - - - - - C C T G G C C C T C T C C C T C A G G G A G A G G G
Gmet_I418	(+)	3930206	3930247	C C C T C A T C C G G C C T T - C G G C C A C C T T C T T C C C T A G A G A G A A G G
Gmet_I419	(-)	3939459	3939494	C C C C C T C A C - - - - - - - - C C T A G C C C T C T C C C A T A G G G A G A G G G
Gmet_I420	(+)	3939517	3939559	C C C T C A T C C G G C C T T - C G G C C A C C T T C T C C C A T G G G G A G A A G G
