# Supplementary material for: The genome sequence of Geobacter metallireducens: features of metabolism, physiology and regulation common and dissimilar to Geobacter sulfurreducens
Source: BMC Microbiol. 2009 May 27;9:109. doi: 10.1186/1471-2180-9-109 (PMC2700814; doi:10.1186/1471-2180-9-109)
Supplement: Additional File 16 — Figure S7. A predicted regulatory short RNA found in the 5' regions of c-type cytochromes and other proteins. This is an alignment of 16 DNA sequences that were matched by nucleotide-level BLAST. The location of Gmet_R3013 suggests that N-acylhomoserine lactone signalling may be under control of this RNA element. Similar sequences were found in the genomes of G. sulfurreducens, G. uraniireducens, and P. propionicus. The sequence strand and start and stop nucleotide positions are indicated. [file 1471-2180-9-109-S16.pdf]

Gmet_R3001	(+)	158167	158284	GATAGACGATAATACTAAACCATCCGCGAGGGTGGGACGGAAAGCCTACAGGGTCTCCCC-GAGACAGCCGGGTCGCC-AAATATCTCAACAG--GATATGC-CGGC--CCCGGCTTTTTTCATG
Gmet_R3002	(-)	264958	265068	AATAGACGACAATACTAAACCATCCGCGAGGATGGGGCGGAAAGCCCATAGGGTCTCACC-GAGACAGCCGGGTTGCCGAAATATTTTTT-------------CGGCGACCCGGCTTTTTCGTTT
Gmet_R3003	(-)	271381	271497	GATACACGACAATACTAAACCATCCGCGAGGATGGGGCGGAAAGCCCACAGGGTCTCACC-GAGACAGCCGGGTTGCCGAAATGTCACCA-----CATTTT--CGACAGCCCGGTTTTTTTGTGC
Gmet_R3004	(+)	325752	325867	GATAGACGATAATACTCAACCATCCGCGAGGATGGGGCGGAAAGCCTACAGGGTCTCTTC-GAGACAGCCGGGTTGCCGAAATATCTCACA----GATATG--CGGC--CCCGGCTTTTTTGTGT
Gmet_R3005	(+)	1079463	1079581	GATAT-CGACAATACTAAACCATCCGCGAGGGTGGGACGGAAAGCCTACAGGGTCTCTCT-GAGACAGCCGGGATGCCGAAATATCACAATTCG-TGATGCT-CGGT--CCCGGCATTTCTTTTT
Gmet_R3006	(-)	1211932	1212051	GATAG-CGATACTACTAAACCATCCGCGAGGATGGGGCGGAAAGCCTACAGGGTCTCATC-GAGACAGCCGGGTTGCCGAACTATCACACCACA-GATAGG--CGGCGACCCGGCTTTTTATTTT
Gmet_R3007	(-)	1212114	1212231	GATAG-CGATACTACTAAACCATCCGCGAGGATGGGGCGGAAAGCCTACAGGGTCTCATC-GAGACAGCCGGGTTGCCGAACTATCACAATA---GATAGG--CGGCGACCCGGCTTTTTTGTTT
Gmet_R3008	(-)	1324366	1324481	GATAGTCGATAATACTAAACCATTCGTGAGAATGGGGCGGAAAGCCTACAGGGTCTTACT-GAGACAGCCGGGTTGCCGAAATATCACCA-----GATATT--CG-CAACCCGGCTTTTTTGTTG
Gmet_R3009	(-)	1324505	1324620	GATAGACGACAATACTAAACCATTCGCGAGAATGGGGCGGAAAGCCTACAGGGTCTTATT-GAGACAGCCGGGTTGCCGAAATATCATTAT----GATAA-T-CGGC--CCCGGCTGTTCTGTTT
Gmet_R3010	(+)	1334236	1334351	GATAGACGACAATACTAAACCATCCGCGAGGGTGGGACGGAAAGCCTACAGGGTCTCCCC-GAGACAGCCGGGTCGCCGAAATATCGCAT----CGATATTC-CGA---CCCGGCTTTTTTTGTG
Gmet_R3011	(+)	1911269	1911387	GATAGACGACAATACTAAACCATCCGCGAGGATGGGGCGGAAAACCCACAGGGTCTCCCT-GAGACAGCCGGGTTGCCGAAATATCAATT-----GATATATATGGCAACCCGGCTGTTTTATTA
Gmet_R3012	(+)	1977790	1977908	GATAGACGATAATACTCAACCATTCGCGAGAATGGGGCGGAAAGCCTATAGGGTCTCATG-CAGACAGCCGGGTTGCCGAAATATCATTTT---AGAATGATGCGGC--CCCGGCTTTTTTGCGT
Gmet_R3013	(+)	2280689	2280806	GATAGACGACAATACTCAACCATCCGTGAGGATGGGGCGGAAAGCCTATTGGGTCTCACC-GAGACAGCCGGGTTGCCGAAATATCTGACCAA-AGATATA---GGC--CCCGGCTTTTTTTTGT
Gmet_R3014	(+)	2447616	2447732	GATACACGACAATACTCAACCATCCGCGAGGATGGGGCGGAAAGCCTACAGGGTCTCCAG-CAGACAGCCGGGTTGCCGAAATATCACT-----CGATGATAGCGGT--CCCGGCTTTTTTGTGC
Gmet_R3015	(+)	3913061	3913175	GATAGACGACAATACTTAACCATTCGCGAGAATGGGGCGGAAAGCCTATAGGGTCTCATG-GAGACAGCCGGGTTGCCGAAATATCACA-----CGATATT--TGGT--CCCGGCTTTTTTATTG
Gmet_R3016	(+)	3913269	3913383	GGTAGACGATAATACTAAACCATTCGCGAGAATGGGGCGGAAAGCCTATAGGGTCTCCCT-GAGACAGCCGGGTTGCCGAAATATCACG-----CGATATT--TGGT--CCCGGCTTTTTTGTGT
