# Supplementary material for: The genome sequence of Geobacter metallireducens: features of metabolism, physiology and regulation common and dissimilar to Geobacter sulfurreducens
Source: BMC Microbiol. 2009 May 27;9:109. doi: 10.1186/1471-2180-9-109 (PMC2700814; doi:10.1186/1471-2180-9-109)
Supplement: Additional File 17 — Table S10. Toxin/antitoxin pairs of G. metallireducens and G. sulfurreducens. This table compares the genes predicted to encode toxin/antitoxin pairs in G. sulfurreducens and G. metallireducens. [file 1471-2180-9-109-S17.pdf]

Table S10. Toxin/antitoxin pairs of *G. metallireducens* and *G. sulfurreducens*.

| ***G. metallireducens* genes** | ***G. sulfurreducens* genes** | **Annotation** |
|---|---|---|
| Gmet_0678, Gmet_0677 | absent | Phd-like antitoxin, ParE-like toxin |
| Gmet_3616, Gmet_3001 | absent | HigB-like toxin C-terminal fragment, VapI-like antitoxin |
| Gmet_A3570, Gmet_A3569 | absent | CopG-like antitoxin, RelE-like toxin |
| Gmet_3077, Gmet_3076 | absent | Phd-like antitoxin, hypothetical nucleic acid-binding toxin |
| Gmet_0220, Gmet_0221 | absent | Phd-like antitoxin, RelE-like toxin |
| Gmet_2502, Gmet_2503 | absent | MazE-like antitoxin, MazF-like toxin |
| Gmet_2602, Gmet_2603 | GSU0737, GSU0736 | hypothetical antitoxin, MazF-like toxin |
| Gmet_2972, Gmet_2973 | absent | VapI-like antitoxin, HigB-like toxin |
| Gmet_2592, Gmet_2591 | absent | HicA-like (antitoxin?), HicB-like (toxin?) |
| absent | GSU0056, GSU0055 | hypothetical antitoxin, ParE-like toxin |
| absent | GSU1353, GSU1354 | Phd-like antitoxin, ParE-like toxin |
| absent | GSU2438, GSU2439 | Phd-like antitoxin, RelE-like toxin |
| absent | GSU2473, GSU2472 | VapB-like antitoxin, VapC-like toxin |
