# Supplementary material for: The genome sequence of Geobacter metallireducens: features of metabolism, physiology and regulation common and dissimilar to Geobacter sulfurreducens
Source: BMC Microbiol. 2009 May 27;9:109. doi: 10.1186/1471-2180-9-109 (PMC2700814; doi:10.1186/1471-2180-9-109)
Supplement: Additional File 18 — Table S11. The CRISPR3 locus of G. metallireducens contains spacers of variable length. The thirteen clustered regularly interspaced short palindromic repeats (CRISPR) of G. metallireducens (consensus sequence GTAGCGCCCGCCTACATAGGCGGGCGAGGATTGAAAC) are far fewer than the thirty-eight of CRISPR1 and one hundred and forty-three of CRISPR2 in G. sulfurreducens. [file 1471-2180-9-109-S18.pdf]

Table S11. The CRISPR3 locus of *G. metallireducens* contains spacers of variable length.

**Spacer**	**Sequence**	**Length (bp)**
Gmet_C001	ACTATGCGCTCCACTTTGGCCCTGATGTTGGCCGT	35
Gmet_C002	CGCCAAATCCGCCGCGCTCCACTCCCGGCAGAAGT TGTC	39
Gmet_C003	CAAGTCCGACTTGAAGATTTGTACTGTCGGCGCC	34
Gmet_C004	ACCATCACCCCAGGGATCGGCACGGCCCGCATCG	34
Gmet_C005	TTGGTGCCCCAGGAAGAGTGGAAACCGCTGATCA GCGTCCG	41
Gmet_C006	TCGACCAACACCCCCGGCACGGTCATGTCGAAGG	34
Gmet_C007	GACACCGGCCTCGACGGGTGGGACGCCGCCGCCT	34
Gmet_C008	GGGAGGGACGGTGATATCGTGCAGGGAATACCAA	34
Gmet_C009	ACCTGATGCTGCCCCTGCGGTCTGCCGTGCGCGG	34
Gmet_C0010	AGGTCATTGGACCGGGCGAAGATGCGGCCCTGA	33
Gmet_C0011	GGAATCAGTTCGCCGCGTTTTGTCCGAAGGTTA	33
Gmet_C0012	ACACAGCCTCTCAATATACCGATATCTCCTATTT	34
