# Supplementary material for: The genome sequence of Geobacter metallireducens: features of metabolism, physiology and regulation common and dissimilar to Geobacter sulfurreducens
Source: BMC Microbiol. 2009 May 27;9:109. doi: 10.1186/1471-2180-9-109 (PMC2700814; doi:10.1186/1471-2180-9-109)
Supplement: Additional File 19 — Figure S8. Miscellaneous multicopy nucleotide sequences found in the G. metallireducens genome. These are alignments of 16 sets of miscellaneous DNA sequences in G. metallireducens that were matched by nucleotide-level BLAST. The sequence strand and start and stop nucleotide positions are indicated. (a) A palindromic sequence-containing family also found in the genomes of G. sulfurreducens, G. uraniireducens, and P. propionicus. (b) Sequences of this type were also found in the genomes of G. sulfurreducens and G. uraniireducens. (c) These sequences are unique to G. metallireducens. (d) The ends of these sequences form inverted repeats. Each sequence begins at the left extremity of the top line (the 5' side of the "+" strand of the chromosome), loops on the right side (switching strands), and continues to the left extremity of the bottom line (the 5' side of the "-" strand of the chromosome). A fragment related to Gmet_R6002 was found in the G. sulfurreducens genome. (e) These sequences are unique to G. metallireducens. (f) Sequences of this type were also found in the genomes of G. uraniireducens and G. bemidjiensis. (g) These sequences contain four octanucleotide repeats (consensus TWGTTGAY), two in tandem on each strand. (h) Sequences of this type were also found in the genome of G. sulfurreducens. (i) These sequences are unique to G. metallireducens. (j) These elements are located near each other. (k) These sequences are unique to G. metallireducens. (l-p) These elements are located near each other. Gmet_R0147 continues as Gmet_R0055, a tRNA-Asn gene (underlined). [file 1471-2180-9-109-S19.pdf]

(a)	Gmet_R2001	(+)	101016	101086	ACATAATTATTGAGCGGTTCACGCGCACGCGCGCATGAACCGCTCATCCCCCCAAACAGAACATTTCTCCC
	Gmet_R2002	(+)	1118182	1118252	AACTTCTTATTCACCGGTTCACGCGCGTGCGCGCGTGAACCGGTGATCTACCCAAATGGAACATTCCTGCC
	Gmet_R2003	(+)	3114734	3114794	AACTCGTGATTGAGCGGTTCACGCGCGCACGCGCGTGAACCGCTCATCCTCCCGAATGGAACATTTCTACC
	Gmet_R2004	(+)	3356561	3356617	AACTCGATATTGAGCGGTTCACGTGC----GCGCGTGAACCGCTCATCTACCCAAATAGAACATTTCGCTG
(b)	Gmet_R4001	(+)	203228	203367	GGCACTTACAAAACTATTGCGGGACCGGTCTGCGTTGTTGCCGCTCGCTCGAAAGCTCAACGTACCAACGGGTACGCGTCGCTTCCTCGTTCGCTTGCGCCTAGCATCCCGGCCTCCTCATCACGTTTTGCAAATGCCTC
	Gmet_R4002	(-)	362288	362427	GGCTCATGCAAAACTACTGCGGGGCCGGTCTGCGTTGTTGCCGCTCACTCGAAAACTCAACGTACCAACGGGTACGCCTCGCTTCCTCGCTCGCTTGCGCCTAGCATCCCAGCCTCCTCATCACGTTTTGCATGAGCCTC
	Gmet_R4003	(+)	2341623	2341762	GGCACTTGCAAAACTATTGCGGGTCCGGTCTGCGTTGTTGCCGCTTGCTCGAAAGCTCAACGTACCAACGGGTACGCTTCACTTCCTCGCTCGCTTGCGCCTAGCATCCCGGCCTCCTCATCACGTTTTGCACGTGCCTC
	Gmet_R4004	(+)	3215640	3215685	GGCTCTTGCAAAACTATTGCGGAGCCGGCCTGCGTTGTTGCCGCTC
	Gmet_R4005	(-)	3802652	3802791	GGCTGTTGAAAAACTATTGCGGGGCCGGTCTGCGTTGTTTCCGCTCGCTCAAAAGCTCAACGTACCAACGGGTACGCCTCGCTTTTTCGCTTGCTTGCGCCTAGCATCCCGGCCTCCTCACGACGTTTTTCAACAGCCTG
	Gmet_R4006	(+)	3837608	3837746	GGCTCATGCAAAACTATTGCGTGGCCGGTTTGCGTTGTTGCCGTTCACTCGAAAGCTCAACGTACCGAGGGGTACGCCTCACTTCCTCGTTCTCTTGCGCC-AGCATCCCGGCCTCCCAATCACGTTTTGCACGTGCCTC
(c)	Gmet_R5001	(-)	350186	350227	CGTAGGGGCAATTCATGAATTGCCCCTACA
	Gmet_R5002	(-)	819590	819619	CGTAAGGGCAATTCATGAATTGCCCCTACA
	Gmet_R5003	(+)	1189899	1189928	CGTAGGGGCAATTCATGAATTGCCCCTACA
	Gmet_R5004	(-)	1190313	1190342	TGTAGGGGCAATTCATGAATTGCCCCTACA
	Gmet_R5005	(+)	1191945	1191974	CGATTGGGCAATTCATGAATTGCCCCTACA
	Gmet_R5006	(-)	1281305	1281334	CGTAGGGGCAATTCATGAATTGCCCCTACC
	Gmet_R5007	(-)	2724815	2724844	CGTAGGGGCAATTCGTGAATTGCCCCTACG
	Gmet_R5008	(-)	3521292	3521321	CGTAGGGGCAATTCATGAATTGCCCCTACA
(d)	Gmet_R6001	(+)	522363	522470	-TAGCCACAGAGGACACAGAGCGCACAGAGAATGGCTTTTGGCCCATGAGTCTT-
		(-)			TTTGCCACAGAGATCACAGAGGACACAGAGAAAAAGGAAACAAACTCCGTTTTCC
	Gmet_R6002	(+)	818411	818485	
		(-)			TCTGCCACAGAGGGCACAGAGAACACAGAGAAAAAACGCGGAAGGAAAGGCTGGCTTCCGGCTTCCGCGCTTGAA
	Gmet_R6003	(+)	1929983	1930129	-TAGCCACAGAGGGCACAGAGATCACAGAGAGAGACAAAACCGTAAGATCCTTTCAAGTCTAATAACCAAAATC
		(-)			TTTGCCACAGAGGTCACAGAGAAAACCAGTAAACCTTTAAGGCCCGGCTCGCATAACACCGCAGTACGTATAGG
	Gmet_R6004	(+)	3847095	3847234	TTTGCCACAGAGGTCACAGAGGACTCAGAGAAAACCAGCATCAAAAAAAATACGCCCGAAGTTACCCTCT-
		(-)			-TAGCCACAGAGGACACAGAGAGCACAGAGAAAAGTCGAAAACAACGCCCACTTACCCTGTACTCTCGGCA
(e)	Gmet_R7001	(+)	960022	960198	GAAAGGAGCGGAGCTCTCGCTCCGAACCGATCCAGCGTCACTAGCCGGTCTTCGTGGCATGTGAACAGCTATTCCCGAGGCTTTCCCTCCGCTGATACCGGAGGTGGTTTCTTGCGGGGATCGGTTCTCCGCGAGAGCTCCGCCCCTTCCGGGAACCAGTGGTGGGAAAGGTGACAA
	Gmet_R7002	(-)	1357623	1357799	GGGAGGGGCGGAGCTCTCGCTCCGAAGCGATCCAGCGTCACCAGCCGGTCTTCGTGGCACGTGAACAGCTGATCCCGGGGCTTTCCCTCCGCTCATGCCTGAGGTGGTTTTTTGCGGGGATCGCTTCTCCGCGAGAGCTCCGCCCCTCCCGGGAGCCAGTGGTAAAAAATGCGCCGA
	Gmet_R7003	(-)	2377928	2378104	GGAAGGGGCGGATCTCTCGCTCCGAAGCGATCCAGCGTCACCAGCCGGTCTTCGTGGCACGTGAACAGCTATTCCCGGGGCTTTCCCTCCGCAAACGCCGTAGGTGGTTTTTTGCGGGGATCGCTTCTCCGCGAGAGCTCCGCCCCTTCCGGGAACCAGTGGTGGAAAAATGTGACAT
	Gmet_R7004	(-)	2424560	2424736	GGAAGGGGCGAAACTCTCGCTCCGAAGCGATCCAGCGTCACTGGTCGGTCTTCGTGGCACGTGAGCCGCTATTCCCGAGGCCTTCCCTCCGTTCATGCCGGCGGTGGTTTTTTGCGGGGATCGCTTCTCCGCGAGAGCCCCGCCCCTTCCGGGAGCCAGTGGTGGATAATGTGACAA
	Gmet_R7005	(-)	2447348	2447524	GGCAGGGGCGGAGCTCTTGCTCCGAAGCGATCCAGCGTCACCAGCCGGTCTTCGTGGCACGTGAACAGCTGTTTCCGAGGCTTTCCCTCCGCCAATACCGAAGGTGGTTTTTTGCGGGGATCGCTTCTCCGCAAGAGCTCCGCCCCTCCCGGGAACCGGCGGTGGAAGATGGGGCAA
	Gmet_R7006	(-)	2766304	2766480	GGGAGGGGCGGGACTCCCGCTCCGAACCGATCCAGCGTCACCAGCCGGTCTTCGTGGCACCTGAGCAGCGGCTTCCGAGGCTTTCCCTCCGCTAATTCCATAGGTGGTTTTTTGCGGGGATCGCTTCTCCGCGAGAGCCCCGCCCCTCCCGGGAACCAGTGGTGGCAAATGCGATAA
	Gmet_R7007	(-)	3071530	3071706	GGAAGGGGTGGAACTCTCGCTCCGAACCGATCCAGCGTCACCAGCCGGTCTTCGTGGCACGTAAACAGCTATTCCCGAGGCTTTCCCTCCGCTAGTGCCTAAGGTGGTTTTTTGCGGGGATCGCTTCTCCGCGAGAGCTCCACCCCTTCCGGGGACTAGCGGTGGAAAATGTGACAT
(f)	Gmet_R8001	(+)	1478405	1478486	TAGATGTTGTATTTGCCGATCTTTGCGTCCACCGGGCCGACAC--TGCGGTCCATCACGGGGTGTATCGGGTGAAGTAAGGGTA
	Gmet_R8002	(+)	1478724	1478805	TCGATGCTGTTGCTACCGACCTTGCCGCCCACCGCGCCGACAC--CGAGGCGCATCATGGGGTGTATCGGGTGAAGGAAGGGTA
	Gmet_R8003	(+)	1479205	1479288	TCGACGCCGTTGCGGCCTACCTTTCCGCCCACAGGGCCGACACACCGAGGCGCATCATGGAGTGTATCGGGTGAAGGAAGGGTA
	Gmet_R8004	(+)	1479993	1480074	TCAGCGCCGTTGCAGCTGATCTTTCCGCCCACCGGTGCGACGC--CGAGGCGCATCATGGGGTGTGTTGGGTGAAGGAAGGATA
(g)	Gmet_R9001	(+)	2828934	2829018	TGAGTACCGGGAAGGGGACGTAGTTGATTTGTTGATTTAAGCGTTGAGTTAGTCAACTAATCAACTACGTCCCCCGATAGACCCC
	Gmet_R9002	(+)	2832835	2832919	TGGGGGCTGGGAAGGGGACGTAGTTGATTTGTTGATTTAAGCGTTGAGTTAGTCAACTAATCAACTACGTCCCCCGATAGACCCC
	Gmet_R9003	(+)	2833766	2833850	TGGGGGCTGGGAAGGGGACGTAGTTGATTTGTTGATTTAAGCGTTGAGTTAGTCAACTAATCAACTACATCCCCCGATAGACCCC
	Gmet_R9004	(-)	2876374	2876397	-----------AAGGGGACGTAGTTGATTTGTTGA--
(h)	Gmet_R0064	(+)	169943	170100	TGACGGATAGCGCGCCGACGGAGCGACTGTGCCCGAGCGCCAGCCGCTGTATGCGAAATGGAGCGTCCAGTGGCCTGTTTGTTTGAACCCAACAGGGTGAGTTACAGGCCGCTAGCGAAATGAGCTGTACAGCGGCGGTGCGAGCGGCACTCGGAGCG
	Gmet_R0066	(-)	289163	289313	TGAGCGCCGTGGGCCATGCGAAGCGAATGTGCCCGAGCGCCAGCCGCTGTAGGCGAAATGGAGCAAAGCCGACCCATCGTGTCTGAGCCCGCAGGGCGAGTTGATGGGGCGGCGCGGAA-TGAGCCCTACAGCGGCGGTGCGAGCGGCACTTTGAGCG
(i)	Gmet_R0068	(+)	352455	352481	GACTTATCCACACCCGCCGTGGAAAGC
	Gmet_R0094	(-)	1808389	1808415	GACTTATCCACACCTGCCGTGGAAAGC
(j)	Gmet_R0074	(+)	790994	791042	CAGCGTCTCCAACGGCAAAATCAGGGTCGGGGTCGGTCCGATCTGATTT
	Gmet_R0075	(+)	793740	793788	CAGCGTCTCCAACGGCAATATCCGGGTCGGGGTCGGCCCGAGCTGATTT
(k)	Gmet_R0091	(+)	1514311	1514335	GAAAGGCGGGAGCGATCCCGCCTTT
	Gmet_R0114	(+)	2682404	2682428	GAAAGGCGGGAGCGATCCCGCCTTT
(l)	Gmet_R0097	(+)	1911093	1911148	ATTTCA-AAGAA-CACGACAGCCCCACTAAAAACTTGCTGGAGTTGAGCGGTTAGTTG
	Gmet_R0099	(+)	1912998	1913055	ATTTCAGAAAAACCACGACACACCCCACCAGAAACTTGCTGGAGTTGAGCGGTTCGTTG
(m)	Gmet_R0105	(-)	2241986	2242101	AACTAATCACGAAAGCACGAAAGGTCGAAAGCACGAAAAAAGGCACAAA-CTCTCCGAACCTTA----CTTTG-----TTTTTCGGGTTT---CAATTTCGTGCTTTCGTGATCAAAG
	Gmet_R0106	(-)	2244162	2244279	AACATCTCACGAAAGCACGAAAGGTTGAAAGTACGAAAAAGAGGCATAAAACACAAAGAGGCTAAAGGTCTTGGGGTTTATTTTTCGTGTTTTCTCAATTTCGTGTTTTCGTGGTGAAAG
	Gmet_R0107	(-)	2246998	2247102	AACTAATCACGAAAGCACGAAAGGTCGAAAGCACGAAAAAAGGCACAAA-CTCTCCGAACCTTA----CTTTG-----TTTTTCGGGTTT---CAATTTCGTGCTTTCGTGATCAAAG
(n)	Gmet_R0110	(+)	2441121	2441147	AAGGCCGGCAGCAATGCCGGCCTTTTT
	Gmet_R0111	(+)	2449471	2449497	AAGGCCGGCAGAAATGCCGGCCTTTTT
(o)	Gmet_R0126	(-)	3079293	3079414	CCCTTGGCACGGTAATCGCTCCCTTGCATTTCTCCCGACACCTCTGCTATCAATGATGACAGCCGGGCCGACCAGCCCGGCTGTCGTGCGTCTGGAGCACCGATTCAACCGTTTCGAGGATT
	Gmet_R0127	(-)	3080770	3080889	CCCTTGGCACGGTAATCGCCTTCTCGTATTTCGCCTGATACCTCTGCTATCAATGGTGACAGCCGGGCCGACCAGCCCGGCTGTTGTGCGTTTTGAGCACCGATTT-GCCGTTTCGA-GATT
(p)	Gmet_R0146	(+)	3893190	3893258	CCAGCAGAAAGCGCCGCAGGGTTAACAACCTGCGGGAACAATTCATTCTCCTGTAGCTCAGTCGGTAGA
	Gmet_R0147	(+)	3910724	3910769	CCAGCAAAAAGCACCGTAGGGTTAACAACCTGCGGAAACAACTCATTCCCCAGTAGCTCAGTCGGTAGA
